# Prevalence and risk factors of hyperuricemia and gout in patients with type 2 diabetes mellitus: a systematic review and meta-analysis

**DOI:** 10.3389/fendo.2026.1857217

**Published:** 2026-06-24

**Authors:** Luyuan Gao, Guancheng Ye, Chunping Liu, Yingkai Gao, Yidi Huang, Qiong Shu, Hao Wang, Hailong Wang

**Affiliations:** Department of Rheumatology, Dongzhimen Hospital, Beijing University of Chinese Medicine, Beijing, China

**Keywords:** gout, hyperuricemia, meta-analysis, prevalence, risk factors, type 2 diabetes mellitus

## Abstract

**Background:**

Type 2 diabetes mellitus (T2DM) is one of the most common metabolic diseases worldwide. hyperuricemia (HUA) and gout are common comorbidities in T2DM patients. This systematic review and meta-analysis aimed to estimate the global prevalence of HUA and gout in patients with T2DM and to identify associated risk factors.

**Methods:**

A systematic search was conducted in PubMed, Embase, Web of Science, and the Cochrane Library to identify observational studies on HUA/gout in patients with T2DM. Two researchers independently performed literature screening, data extraction, and quality assessment. The quality of included studies was assessed using the Newcastle-Ottawa Scale and the AHRQ Scale. Statistical analyses were performed using Stata 12.0 software. A random-effects model was used to pool prevalence estimates, with subgroup and sensitivity analyses to explore heterogeneity. Publication bias was assessed using Egger’s test.

**Results:**

Eighty-seven studies comprising 977,573 T2DM patients were included. The pooled prevalence of HUA was 22.0% (95% CI: 20.1-24.0%, 95% PI: 4.9-39.2%), and that of gout was 6.0% (95% CI: 4.4-7.5%). Meta-regression identified geographic region as a significant source of heterogeneity, with the highest HUA prevalence in Africa and North America, and lowest in South America. Risk factors for HUA included impaired renal function, obesity, dyslipidemia, hypertension, metabolic syndrome, and alcohol consumption, while elevated HbA1c was inversely associated with HUA. Male sex was a significant risk factor for gout.

**Conclusion:**

The prevalence of HUA and gout is significantly higher in patients with T2DM than in the general population. Impaired renal function, obesity, dyslipidemia, hypertension, MetS, and alcohol consumption are the primary risk factors. Routine serum uric acid monitoring and early screening for high-risk individuals should be incorporated into the clinical management of T2DM.

**Systematic Review Registration:**

https://www.crd.york.ac.uk/PROSPERO/view/CRD420261359583, identifier CRD420261359583.

## 1 Introduction

Type 2 diabetes (T2DM) is one of the most common metabolic diseases worldwide. According to the latest data from the International Diabetes Federation (IDF), approximately 589 million people worldwide had diabetes in 2024 (a prevalence of 11.1%), with T2DM accounting for over 90% of cases. The number of people with diabetes is projected to rise to 783 million by 2045. The high prevalence is accompanied by a substantial economic burden, with global annual health expenditures related to diabetes have exceeded $1.05 trillion, placing a heavy burden on national healthcare systems and socioeconomic conditions (1).

Hyperuricemia (HUA) and gout are common complications in patients with T2DM (2). Uric acid is the end product of purine metabolism. When serum uric acid (SUA) levels exceed its physiological solubility, urate crystals can form and deposit in joints and soft tissues, triggering gouty arthritis (3). In addition to gout, elevated SUA levels are closely associated with kidney damage (4), cardiovascular disease (5), and metabolic syndrome (MetS) (6). T2DM and HUA/gout share common pathogenic mechanisms, such as insulin resistance, oxidative stress, and activation of the renin-angiotensin-aldosterone system (RAAS), as well as overlapping complications (7, 8). Furthermore, the presence of HUA and gout can further increase the risk of vascular complications in patients with T2DM, exacerbate kidney damage, and adversely affect patients’ quality of life and prognosis (9, 10).

Several previous meta-analyses have confirmed the close association between T2DM and HUA/gout from various perspectives. One meta-analysis found that for every 1 mg/dL increase in SUA, the risk of T2DM increased by 6% (11). Another meta-analysis found that patients with HUA/gout had a higher prevalence of diabetes (12). Although existing meta-analyses have explored the relationship between diabetes and HUA/gout, they have failed to comprehensively integrate global data, thoroughly investigate sources of heterogeneity, or systematically analyze risk factors for the condition (13). In this study, we conducted a systematic review and meta-analysis to integrate the global evidence on the prevalence of HUA and gout in patients with T2DM. Through subgroup analysis and meta-regression, we explore sources of heterogeneity such as geographic regions and diagnostic criteria. Quantitative analysis is used to assess the strength of association for each risk factor, with the aim of providing evidence-based medical evidence to help clinicians identify high-risk populations early and formulate individualized prevention and treatment strategies.

## 2 Materials and methods

### 2.1 Search strategy

Two researchers (Gao and Ye) independently conducted a systematic search across four electronic databases (PubMed, Embase, Web of Science, and the Cochrane Library) to identify eligible studies published from the inception of each database through February 27, 2026. Key search terms included “Type 2 Diabetes Mellitus,” “Hyperuricemia”, “Gout”, and their synonyms. In addition, the reference lists of included studies and relevant reviews were manually screened to identify potentially eligible studies that might have been missed in the database search. The detailed search strategy is shown in **Supplementary Table 1**. The study selection process followed the PRISMA guidelines, and the meta-analysis protocol has been registered with PROSPERO (CRD420261359583).

### 2.2 Study selection

Two researchers (Gao and Ye) independently screened the titles and abstracts of all studies, any disagreements were resolved through consensus with the involvement of a third researcher (Wang). The inclusion criteria were as follows (1): studies enrolling adult patients (aged ≥18 years) with a confirmed diagnosis of T2DM; (2) observational study designs, including cross-sectional, cohort, and case-control studies; (3) studies reporting at least one of the following primary outcomes: the prevalence of HUA or gout in T2DM patients, or risk factors associated with HUA/gout in T2DM patients with corresponding odds ratios (ORs) and 95% confidence intervals (CIs).

Exclusion criteria are as follows: (1) Studies involving patients with type 1 diabetes, gestational diabetes, or other specific types of diabetes; (2) Reviews, meta-analyses, conference abstracts, or animal studies; (3) Studies from which specific case numbers or prevalence data for HUA/gout cannot be extracted; (4) Studies published in languages other than English; (5) studies restricted to highly selected high-risk subpopulations (for example, end-stage renal disease or dialysis patients), which were excluded to minimize selection bias; (6) If two or more studies were conducted on the same population, data most relevant to the objectives of this study were selected for analysis.

### 2.3 Data extraction

Two researchers (Gao and Ye) independently extracted data using a pre-designed standardized Excel spreadsheet. The extracted information included the first author, year of publication, country of study, study design, demographic characteristics, total sample size, number of cases, diagnostic criteria for HUA/gout, and effect sizes of relevant risk factors. After extraction, the two researchers cross-checked the data, and any discrepancies were resolved through consensus.

### 2.4 Quality assessment

Two independent researchers assessed the methodological quality of the included studies using bias risk assessment tools tailored to each study design. Cross-sectional studies were evaluated using the AHRQ scale (0–11 points), while cohort studies were assessed using NOS (0–9 points). AHRQ scores ≥8 or NOS scores ≥7 were considered to indicate high-quality articles with a low risk of bias.

### 2.5 Statistical methods

All statistical analyses and graphing were performed using Stata 12.0 software. Pooled prevalence estimates and their corresponding 95% CIs were used as summary measures for HUA and gout in patients with T2DM. Given the inherently high heterogeneity of prevalence meta-analyses, the following multi-layered strategies were employed to characterize and contextualize between-study variation: (1) a random-effects model was used to pool prevalence estimates across all studies; (2) for subgroups with at least 10 studies, 95% prediction intervals (PIs) were calculated to estimate the range within which the true effect size of a future comparable study is likely to fall after accounting for heterogeneity; (3) pre-specified subgroup analyses were conducted across geographic region, study quality, sample size, diagnostic criteria, and patient clinical characteristics (including sex, age, and glycemic control status); (4) random-effects meta-regression was performed, with covariates including geographic region, diagnostic criteria, sample size, study quality, and exclusion of populations receiving urate-lowering therapy; and (5) leave-one-out sensitivity analyses were conducted to assess the robustness of the findings. All subgroup analyses and meta-regression were pre-specified in the study protocol.

For the analysis of risk factors, ORs and 95% CIs were pooled for binary variables; for continuous variables such as age and waist circumference, the summary OR per one-unit increase and its 95% CI were extracted and pooled. Heterogeneity across studies was assessed using the Q-test and I² statistic. If P < 0.1 or I² > 50%, significant heterogeneity was considered present, and a random-effects model was used for pooling. Leave-one-out sensitivity analyses were performed to verify the robustness of the conclusions. When the number of included studies was ≥10, publication bias was assessed using contour-enhanced funnel plots and Egger’s test, with P > 0.05 indicating no significant bias.

## 3 Results

### 3.1 Literature search and study selection

**Figure 1:**
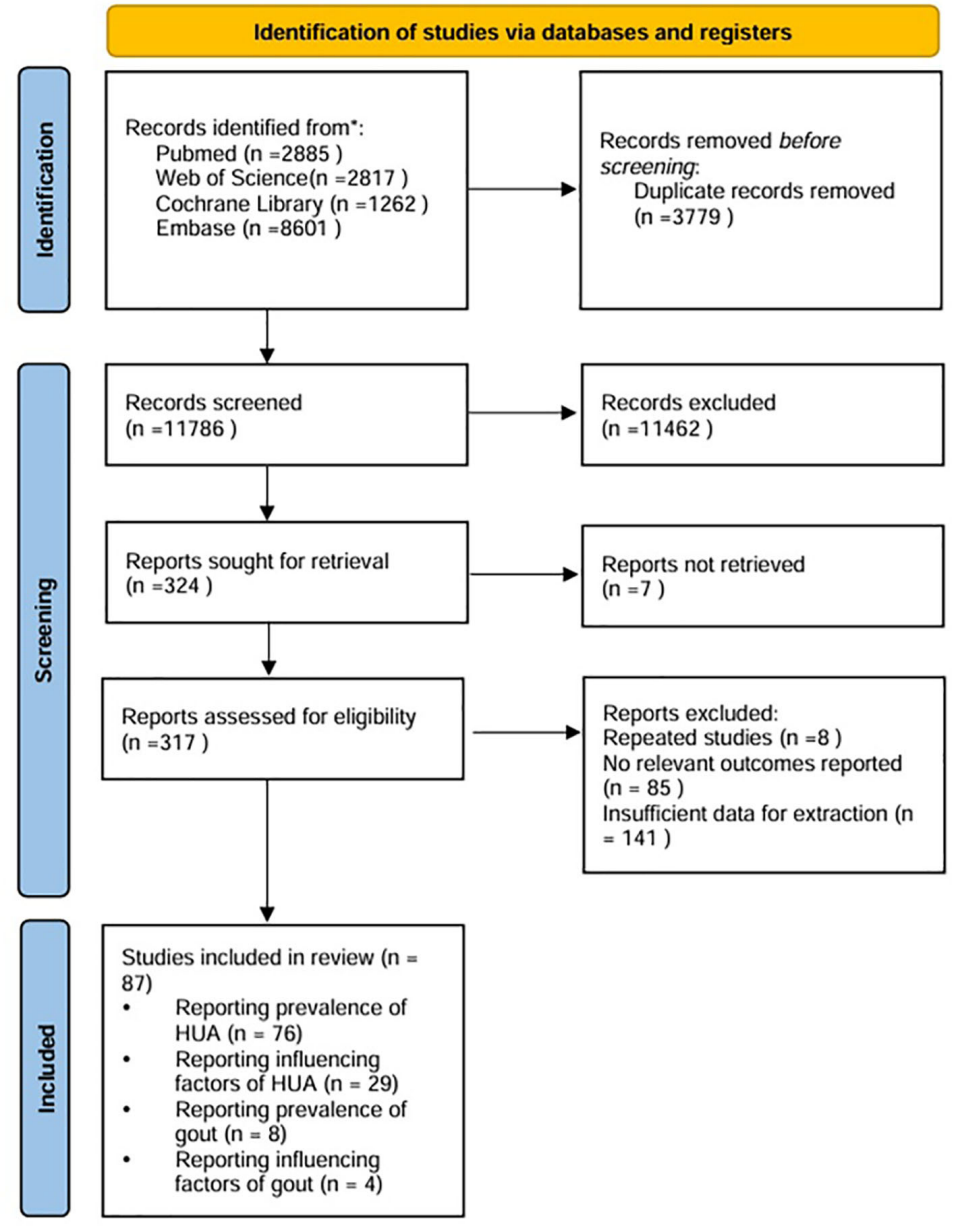
PRISMA flow diagram of study selection process.

**Figure 1** illustrates the literature screening process. A total of 15,565 studies were retrieved from the databases. Duplicate articles and studies that did not meet the inclusion criteria were subsequently excluded. After a comprehensive review of the full texts, a total of 87 eligible observational studies were included in the analysis (14–100). To ensure the clinical comparability of the pooled effect sizes, this study excluded studies that used non-consensus HUA diagnostic cutoffs or those inconsistent with mainstream guidelines. For example, two studies that used 5.5 mg/dL and 8.0 mg/dL as diagnostic thresholds, respectively, were excluded (101, 102). Although diagnostic criteria varied somewhat in studies from the African region, these criteria were locally adopted. To maintain regional representativeness, all such studies were included. Additionally, six studies were excluded due to potential overlap in study populations with those in the included literature (103–108). Among these, 80 were cross-sectional studies (14–25, 27–55, 57–72, 74–85, 87–89, 91–94, 96, 98–100), and 7 were cohort studies (26, 56, 73, 86, 90, 95, 97). Of these, 76 studies reported the prevalence of HUA in patients with T2DM (14–67, 69, 70, 72–91), 29 included meta-analyses of risk factors for HUA in T2DM patients (14–16, 20, 23, 25, 28, 38–41, 43, 45, 47, 48, 58, 60, 64, 68, 70, 71, 75–80, 82, 85), 8 studies reported the prevalence of gout in T2DM patients (93–100), and 4 studies reported risk factors for gout in T2DM patients (92–94, 98). The studies encompassed 977,573 T2DM patients, with publication years ranging from 2001 to 2026. The included studies covered multiple regions, including Asia (n=57) (15–17, 23, 24, 26, 28, 29, 32, 34, 35, 37–45, 47, 48, 52, 57, 58, 60–71, 75–78, 81–87, 89, 91–93, 95–97, 99, 100), Africa (n=15) (14, 18, 20, 21, 25, 27, 30, 31, 33, 51, 53, 54, 72, 79, 80), Europe (n=10) (22, 36, 46, 49, 50, 55, 56, 73, 90, 94), South America (n=2) (19, 59), North America (n=2) (15, 74), Oceania (n=1) (98). Regarding the assessment of study quality, based on the AHRQ and NOS quality assessment criteria, 48 studies were rated as high quality, and 39 were rated as moderate/low quality. Detailed study characteristics and quality assessment results are presented in **Supplementary Tables 1–4**.

### 3.2 Prevalence and heterogeneity analysis of T2DM with HUA

#### 3.2.1 Overall prevalence, publication bias, and sensitivity analysis

A total of 76 studies reported the prevalence of HUA, involving 136,985 patients with T2DM, of whom 29,590 had HUA. Substantial between-study heterogeneity was observed (p < 0.001, I² = 98.8%), and a random-effects model was applied for pooling. The pooled prevalence of HUA in patients with T2DM was 22.0% (95% CI: 20.1–24.0%, 95% PI: 4.9–39.2%). The wide prediction interval underscores the limitation of relying solely on the pooled point estimate. To explore sources of heterogeneity, subgroup analyses and meta-regression were conducted. Publication bias was assessed using a funnel plot and Egger’s test. Visual inspection of the funnel plot revealed some asymmetry, but Egger’s test yielded a P value of 0.094 (P > 0.05), indicating no significant publication bias (**Supplementary Figure 1**). Leave-one-out sensitivity analysis showed that the re-pooled prevalence ranged from 21.6% to 22.2%, confirming the robustness of the result (**Supplementary Figure 2**).

#### 3.2.2 Subgroup analysis of study characteristics

We conducted subgroup analyses based on the methodological characteristics of the included studies (**Table 1**). HUA prevalence varied substantially across geographic regions: Africa (29.0%, 95% CI: 24.7–33.3%, 95% PI: 11.1–46.8%), Asia (20.2%, 95% CI: 17.7–22.7%, 95% PI: 2.7–37.8%), Europe (20.6%, 95% CI: 16.3–24.9%), North America (28.6%, 95% CI: 23.8–33.3%), and South America (17.3%, 95% CI: 6.2–28.4%). Overall, HUA prevalence was higher in Africa and North America, whereas the estimate for South America was slightly below the average. However, given the small number of studies from North and South America, these regional findings should be interpreted with caution. Furthermore, the wide 95% PIs for Africa and Asia reflect substantial residual heterogeneity within these regions.

**Table 1:** Overall and subgroup analysis results of HUA prevalence.

Subgroup	no of Cohort	HUA Prevalence (95%CI) %	P for heterogeneity	I2%	P for overall effect	95%PI (%)
Overall Prevalence	76	22.0 (20.1 , 24.0)	<0.001	98.8	<0.001	4.9-39.2
Diagnostic Criteria						
M>7.0, F>6.0 mg/dL	43	22.5 (20.0 , 25.0)	<0.001	98.7	<0.001	5.8-39.1
>7.0 mg/dL	20	19.3 (16.9 , 21.6)	<0.001	97.6	<0.001	8.2-30.9
>6.8 mg/dL	3	28.8 (25.8 , 31.8)	0.336	8.4	<0.001	/
Other Criteria	6	20.7 (14.8 , 26.5)	<0.001	96.3	<0.001	/
Clinical Diagnosis	4	26.2 (4.8 , 47.6)	<0.001	99.5	0.016	/
Region						
Africa	15	29.0 (24.7 , 33.3)	<0.001	90.6	<0.001	11.1-46.8
Asia	48	20.2 (17.7 , 22.7)	<0.001	99.2	<0.001	2.7-37.8
Europe	9	20.6 (16.3 , 24.9)	<0.001	95.4	<0.001	/
South America	2	17.3 (6.2 , 28.4)	<0.001	91.9	0.002	/
North America	2	28.6 (23.8 , 33.3)	0.002	89.9	<0.001	/
ULT treatment						
Excluded	52	21.3 (18.6 , 24.1)	<0.001	97.2	<0.001	7.2-35.5
Not excluded	24	22.3 (19.8 , 24.8)	<0.001	99.1	<0.001	4.0-40.6
Study Quality						
High	42	21.0 (18.3 , 23.6)	<0.001	99.1	<0.001	3.5-38.5
Moderate / Low	34	23.4 (20.4 , 26.4)	<0.001	98.1	<0.001	5.7-41.2
Sample Size						
<1000	43	23.3 (20.7 , 25.9)	<0.001	95.0	<0.001	6.3-40.4
≥1000	33	20.4 (17.5 , 23.4)	<0.001	99.4	<0.001	2.7-38.2
Sex						
Male	48	22.2 (20.4 , 23.9)	<0.001	95.4	<0.001	10.4-34.0
Female	45	23.1 (19.0 , 27.3)	<0.001	99.2	<0.001	0-51.5
Age						
≥60	15	29.8 (24.5 , 35.0)	<0.001	99.3	<0.001	8.0-51.6
<60	8	22.3 (19.3 , 25.2)	0.185	29.3	<0.001	/
Duration of T2DM						
≥10	8	31.4 (16.6 , 46.1)	<0.001	99.4	<0.001	/
<10	12	24.9 (14.4 , 35.4)	<0.001	99.3	<0.001	0-67.5
BMI						
Overweight	8	35.8 (22.7 , 49.0)	<0.001	99.5	<0.001	/
Obesity	9	34.6 (25.9 , 43.2)	<0.001	96.8	<0.001	/
Lipid Profile						
Dyslipidemia	6	28.0 (20.7 , 35.3)	<0.001	94.9	<0.001	/
Hyperlipidemia	4	25.3 (16.8 , 33.8)	<0.001	96.8	<0.001	/
Hypertriglyceridemia	10	37.6 (28.4 , 46.8)	<0.001	96.9	<0.001	2.9-72.4
Hypercholesterolemia	6	37.7 (20.2 , 55.3)	<0.001	98.6	<0.001	/
High LDL-C	5	37.3 (17.6 , 57.1)	<0.001	98.6	<0.001	/
Low HDL-C	10	39.6 (25.0 , 54.3)	<0.001	98.9	<0.001	0-96.1
HbA1c						
≥7	8	19.0 (13.6 , 24.4)	<0.001	98.9	<0.001	/
<7	6	29.1 (19.1 , 39.1)	<0.001	96.6	<0.001	/
Use of Hypoglycemic drugs						
Oral or insulin	2	46.4 (45.2 , 47.5)	0.543	0.0	<0.001	/
Oral Only	5	21.2 (13.0 , 29.3)	<0.001	93.5	<0.001	/
Insulin	7	22.1 (13.5 , 30.7)	<0.001	98.8	<0.001	/
Smoke	22	23.7 (20.4 , 27.0)	<0.001	92.3	<0.001	9.3-38.2
Alcohol	19	26.5 (21.9 , 31.2)	<0.001	94.8	<0.001	5.9-47.2
Family History of DM	5	28.3 (20.0 , 36.6)	<0.001	95.5	<0.001	/
Central Obesity	9	33.7 (22.6 , 44.7)	<0.001	98.3	<0.001	/
Hypertension	28	30.1 (26.7 , 33.5)	<0.001	98	<0.001	11.6-48.6
MetS	8	32.1 (26.1 , 38.1)	<0.001	86	<0.001	/
eGFR < 60	15	38.7 (32.0 , 45.5)	<0.001	98.6	<0.001	10-67.3
IHD	7	24.8 (18.1 , 31.6)	<0.001	87.5	<0.001	/
DR	9	31.4 (24.2 , 38.6)	<0.001	93.4	<0.001	/
Use of Diuretic drugs	6	48.1 (41.5 , 54.7)	0.008	68.3	<0.001	/
Use of Lipid-lowering drugs	5	28.9 (17.9 , 39.8)	<0.001	97.9	<0.001	/

HUA, hyperuricemia; CI, confidence interval; PI, prediction interval; ULT, urate-lowering therapy; T2DM, type 2 diabetes mellitus; BMI, body mass index; WC, waist circumference; eGFR, estimated glomerular filtration rate; MetS, metabolic syndrome; LDL-C, low-density lipoprotein cholesterol; HDL-C, high-density lipoprotein cholesterol; IHD, ischemic heart disease; DR, diabetic retinopathy; SD, standard deviation.

Furthermore, no significant difference in the pooled prevalence was observed between high-quality studies (21.0%) and low/moderate-quality studies (23.4%), between studies with large sample sizes ≥1000 (20.4%) and small sample sizes <1000 (23.3%), or between studies that excluded (21.3%) and those that did not exclude (22.3%) participants receiving urate-lowering therapy (**Table 1**). This suggests that the aforementioned methodological differences did not significantly interfere with the overall pooled effect of this study, and the results are reasonably reliable.

#### 3.2.3 Subgroup analysis by diagnostic criteria

Stratified analysis by HUA diagnostic criteria was performed, and substantial heterogeneity was observed across all subgroups. Forty-three studies used sex-specific criteria (M >7.0, F >6.0 mg/dL), yielding a pooled prevalence (22.5%, 95% CI: 20.0–25.0%, 95% PI: 5.8–39.1%) that was nearly identical to the overall estimate (22.0%, 95% CI: 20.1–24.0%, 95% PI: 4.9–39.2%). Twenty studies applied a uniform threshold of >7.0 mg/dL irrespective of sex, producing a slightly lower pooled prevalence (19.3%, 95% CI: 16.9–21.6%, 95% PI: 8.2–30.9%), which nonetheless fell entirely within the overall PI, supporting the robustness of pooling across diagnostic criteria. Three studies used a uniform threshold of >6.8 mg/dL, with a pooled prevalence of 28.8% (95% CI: 25.8–31.8%). Six studies adopted other criteria, yielding a pooled prevalence of 20.7% (95% CI: 14.8–26.5%). And four studies did not explicitly report the diagnostic criteria, mentioning only “Clinical Diagnosis”, with a pooled prevalence of 26.2% (95% CI: 4.8–47.6%) (**Table 1**).

#### 3.2.4 Subgroup analysis by sex

Given that sex is an important determinant of uric acid metabolism, we conducted sex-stratified subgroup analyses (**Supplementary Tables 5**, **6**; **Supplementary Figures 4**, **5**). Among 48 studies reporting prevalence in males (47,966 patients), the pooled HUA prevalence was 22.2% (95% CI: 20.4-23.9%, 95% PI: 10.4-34.0%), and among 45 studies in females (44,714 patients), the pooled prevalence was 23.1% (95% CI: 19.0-27.3%, 95% PI: 0-51.5%). Further stratification by region showed that Africa had the highest HUA prevalence in both sexes (M: 30.4%, F: 34.9%), followed by Asia (M: 20.2%, F: 20.3%) and Europe (M: 22.4%, F: 22.6%), consistent with the overall regional pattern. Notably, the diagnostic threshold had a marked influence on female prevalence: when the >6.0 mg/dL cutoff was applied, the pooled prevalence in females was 25.6% (95% CI: 20.5-30.7%), but only 14.3% (95% CI: 11.0-17.6%) under the >7.0 mg/dL threshold. In contrast, the most commonly used threshold in males was >7.0 mg/dL (pooled prevalence: 21.2%, 95% CI: 19.3-23.0%). This finding highlights the considerable influence of diagnostic criteria on prevalence estimates in females.

#### 3.2.5 Subgroup analysis based on clinical characteristics and comorbidities

To identify high-risk subgroups of T2DM patients with HUA, we extracted the prevalence of HUA across various clinical phenotypes from the literature and conducted subgroup analyses (**Table 1**).

Among 15 studies of patients aged ≥60 years, the pooled HUA prevalence was 29.8% (95% CI: 24.5-35.0%, 95%PI: 8.0-51.6%). While 8 studies reported a prevalence of 22.3% (95% CI: 19.3-25.2%) among patients <60 years of age (**Supplementary Figure 6**). Regarding patients with different durations of T2DM, 8 studies reported the prevalence of HUA in patients with a T2DM duration of ≥10 years, showing a prevalence of 31.4% (95% CI: 16.6-46.1%), 12 studies reported a prevalence of HUA of 24.9% (95% CI: 14.4-35.4%, 95%PI: 0-67.5%) among patients with a T2DM duration <10 years (**Supplementary Figure 9**). Five studies reported on a subgroup of patients with a family history of T2DM, showing a prevalence of HUA of 28.3% (95% CI: 20.0-36.6%) (**Supplementary Figure 10**). It is evident that the prevalence of HUA increases with advancing age and longer duration of T2DM, and that individuals with a family history of T2DM also have a higher prevalence of HUA.

Regarding lifestyle factors, 22 studies reported on a subgroup of T2DM patients who have the habit of smoking, with a prevalence of HUA of 23.7% (95% CI: 20.4-27.0%, 95%PI: 9.3-38.2%) (**Supplementary Figure 7**), which was not significantly different from the overall prevalence. Nineteen studies reported on a subgroup of T2DM patients with alcohol consumption, with a prevalence of HUA of 26.5% (95% CI: 21.9-31.2%, 95%PI: 5.9-47.2%) (**Supplementary Figure 8**), which was slightly higher than the overall prevalence of HUA.

Regarding abnormal body weight and metabolic disorders, T2DM patients with overweight (35.8%, 95% CI: 22.7-49.0%, 8 studies), obesity (34.6%, 95% CI: 25.9-43.2%, 9 studies), and central obesity (33.7%, 95% CI: 22.6-44.7%, 9 studies) showed substantially higher HUA prevalence than the overall estimate (**Supplementary Figures 11–1**, **11-2**). Similarly, the prevalence of HUA was also elevated among patients with MetS (32.1%, 95% CI: 26.1-38.1%, 8 studies) and hypertension (30.1%, 95% CI: 26.7-33.5%, 95%PI: 11.6-48.6%, 28 studies) (**Supplementary Figures 13**, **14**). In the subgroup with concomitant lipid metabolism abnormalities, the prevalence of HUA was also high among patients with dyslipidemia (28.0%, 95% CI: 20.7-35.3%, 6 studies), hyperlipidemia (25.3%, 95% CI: 16.8-33.8%, 4 studies), and hypercholesterolemia (37.7%, 95% CI: 20.2-55.3%, 6 studies), hypertriglyceridemia (37.6%, 95% CI: 28.4-46.8%, 10 studies), and elevated LDL-C (37.3%, 95% CI: 17.6-57.1%, 5 studies), and low HDL-C (39.6%, 95% CI: 32.0-54.3%, 10 studies) (**Supplementary Figure 12**). It is evident that T2DM patients with other components of MetS, such as obesity, hypertension, and dyslipidemia, have a higher prevalence of HUA.

In addition, the prevalence of HUA was elevated in T2DM patients with ischemic heart disease (24.8%, 95% CI: 18.1-31.6%, n=7 studies) and diabetic retinopathy (31.4%, 95% CI: 24.2-38.6%, n=9 studies) (**Supplementary Figures 16**, **17**). Furthermore, T2DM patients with impaired renal function (eGFR < 60 mL/min/1.73 m²) had the highest HUA prevalence (38.7%, 95% CI: 32.0-45.5%, 95% PI: 10.0-67.3%, n=15 studies) (**Supplementary Figure 15**).

Regarding glycemic control, the prevalence of HUA in the HbA1c ≥ 7% subgroup was 19.0% (95% CI: 13.6-24.4%, 8 studies), which was lower than the 29.1% (95% CI: 19.1-39.1%, 6 studies) observed in the HbA1c < 7% subgroup (**Supplementary Figure 18**). Regarding medication history, the prevalence of HUA was 28.9% (95% CI: 17.9-39.8%, 5 studies) and 22.1% (95% CI: 13.5-30.7%, 7 studies) among patients using lipid-lowering agents and insulin, respectively (**Supplementary Figures 20**, **21**). Whereas in the subgroup of patients using diuretics, the prevalence of HUA was 48.1% (95% CI: 41.5%-54.7%, 6 studies) (**Supplementary Figure 19**), which was significantly higher than the overall prevalence.

#### 3.2.6 Meta-regression analysis of HUA prevalence

To explore the potential sources of high heterogeneity, we performed a meta-regression based on study characteristics. The results showed that, among the included variables, the geographic region where the study was conducted was the only statistically significant variable (p=0.010), explaining 13.05% of the heterogeneity. Specifically, the prevalence in Africa was significantly higher than that in Asia (p=0.001), indicating that geographic differences are a key factor leading to the extreme variability in prevalence estimates within this subgroup. In contrast, the mean age of the study population (p = 0.778), the sex composition of the study population (p = 0.556), the year of publication (p = 0.677), diagnostic criteria (p = 0.364), sample size (p=0.690), study quality (p=0.193), and whether participants receiving ULT were excluded (p=0.794) did not show significant associations (see **Supplementary Table 7**; **Supplementary Figure 3**).

### 3.3 Analysis of the prevalence of gout in patients with T2DM

A total of 8 studies reported the prevalence of gout, involving 827,819 patients with T2DM. Due to significant heterogeneity among the studies (p < 0.001, I² = 99.5%), a random-effects model was used for data pooling. The results showed that the overall prevalence of gout in patients with T2DM was 6.0% (95% CI: 4.4%-7.5%) (**Supplementary Figure 22**). Sensitivity analyses conducted by sequentially excluding individual studies indicated that the pooled prevalence ranged between 4.60% and 6.76%, confirming that the meta-analysis results regarding gout prevalence were robust and not significantly influenced by any single study (**Supplementary Figure 25**).

To further explore the sources of heterogeneity and the prevalence of the condition among different subgroups, subgroup analyses were conducted (**Table 2**). With regard to geographic region, the pooled prevalence of gout in Asian studies was 4.0% (95% CI: 3.1-4.9, 6 studies), while the prevalence in non-Asian studies was 15.9% (95% CI: 4.0-27.8%, 2 studies) (**Supplementary Figure 26**). Regarding diagnostic criteria, the prevalence in studies using self-reported data was 4.2% (95% CI: 3.6-4.7%, 2 studies), while the prevalence based on medical records was 5.1% (95% CI: 3.1-7.0%,5 studies), and the prevalence in studies using ACR/EULAR criteria was 5.7% (95% CI: 5.3-6.0%, 1 study) (**Supplementary Figure 27**). Additionally, high study quality (4.1%), moderate-to-low study quality (9.7%), large sample size (4.9%), and small sample size (8.5%) may be sources of heterogeneity. However, due to the small number of studies, these findings should be interpreted with caution (**Supplementary Figures 28**, **29**).

**Table 2:** Subgroup analysis results of gout prevalence.

Subgroup	No. of cohort	Gout prevalence (95%CI) %	P for heterogeneity	I2%	P for overall effect
Overall Prevalence	8	6.0 (4.4 - 7.5)	<0.001	99.5	<0.001
Diagnostic criteria					
ACR/EULAR	1	5.7 (5.3 - 6.0)	/	/	<0.001
Medical records	5	5.1 (3.1 -7.0)	<0.001	99.6	<0.001
Self-Reported	2	4.2 (3.6 – 4.7)	/	/	0.16
Sample size					
>= 1000	6	4.9 (3.3 - 6.6)	<0.001	99.6	<0.001
< 1000	2	8.5 (6.5 – 10.6)	/	/	<0.001
Study quality					
High	4	4.1 (3.0 - 5.2)	<0.001	99	<0.001
Moderate/Low	4	9.7 (4.5 – 14.8)	<0.001	99.4	<0.001
Region					
Asia	6	4.0 (3.1 - 4.9)	<0.001	98.3	<0.001
Non-Asia	2	15.9 (4.0 - 27.8)	<0.001	95.5	0.009
Sex					
Male	3	6.3 (5.9 -6.8)	<0.001	99.7	<0.001
Female	3	13.6 (6.4 - 20.9)	<0.001	99.5	0.009
Kidney injury					
eGFR < 60	3	6.9 (1.7 - 12.2)	0.091	58.3	<0.001

### 3.4 Analysis of risk factors for HUA in patients with T2DM

To identify factors associated with HUA in patients with T2DM, we conducted a meta-analysis of 28 studies (**Table 3**). Due to significant heterogeneity among most variables across studies, a random-effects model was used for the meta-analysis of all variables except hip circumference, duration of diabetes, TyG, and HbA1c. Overall, impaired renal function (eGFR < 60, elevated creatinine, elevated BUN), obesity and central obesity, hypertriglyceridemia and low HDL-C, hypertension, high systolic blood pressure (SBP), MetS, elevated TyG, and alcohol consumption were identified as risk factors for HUA, while elevated HbA1c was inversely associated with HUA.

**Table 3:** Summary of pooled risk factors of HUA.

Risk factor	No. of cohort	OR (95%CI)	P for heterogeneity	I2%	P for overall effect	Model
**Sex (Female)**	9	1.63 (0.87 - 3.04)	<0.001	97.5	0.124	Random
**Age**						Random
Per 1 year increase in age	5	0.98 (0.96 - 0.99)	<0.001	86.8	0.013	
40–49 vs <40	1	0.63 (0.15 - 2.61)	/	/	0.521	
50–59 vs <40	1	1.26 (0.45 - 3.60)	/	/	0.659	
≥60 vs <40	1	2.07 (0.74 - 5.82)	/	/	0.166	
≥45 vs <45	1	1.90 (1.16 - 3.10)	/	/	0.01	
**eGFR**						Random
<60 vs ≥60	5	3.75 (2.36 - 5.95)	0.001	79.7	<0.001	
Per 1 mL/min/1.73m2 increase in eGFR	3	0.98 (0.97 - 0.99)	<0.001	87.2	<0.001	
**BMI**						Random
Overweight	5	1.75 (1.08 - 2.86)	0.003	75.1	0.024	
Obesity	3	2.23 (1.34 - 3.70)	0.118	53.3	0.002	
Per 1kg/m^2^ increase in BMI	8	1.08 (1.05 - 1.11)	0.062	47.9	<0.001	
**TG**						Random
Hypertriglyceridemia	3	1.38 (1.00 - 1.92)	<0.001	88.1	0.053	
Per 1 mmol/L increase in TG	6	1.16 (1.06 - 1.26)	0.015	67.5	0.001	
**HDL-C**						Random
Low HDL-C	4	1.27 (1.16 - 1.38)	0.621	0	<0.001	
Per 1 mmol/L decrease in HDL-C	4	1.97 (1.11 - 3.50)	0.007	75.5	0.02	
**TC**						Random
Hypercholesterolemia	2	1.18 (0.83 - 1.68)	<0.001	92.4	0.351	
Per 1 mmol/L increase in TC	2	0.97 (0.70 - 1.36)	0.013	83.6	0.865	
**LDL-C**						Random
High LDL-C	2	1.10 (1.00 - 1.20)	0.48	0	0.044	
Per 1 mmol/L increase in LDL-C	2	1.18 (0.87 - 1.60)	0.067	70.3	0.297	
**Waist Circumference**						Random
High WC	4	2.25 (1.33 - 3.81)	0.006	75.7	0.002	
Per 1 cm increase in WC	3	1.03 (1.01 - 1.04)	0.462	0	<0.001	
**Hip Circumference**						Fixed
Per 1 cm increase in HC	3	1.03 (1.02 - 1.05)	0.517	0	<0.001	
**Hypertension**	6	1.84 (1.08 - 3.12)	<0.001	93.2	0.025	Random
**SBP**						Random
High SBP	2	2.79 (1.09 - 7.16)	0.098	63.5	0.033	
Per 1 mmHg increase in SBP	1	1.01 (0.99 - 1.03)	/	/	0.325	
**DBP**						Random
High DBP	3	2.09 (0.97 - 4.48)	0.018	75.1	0.058	
Per 1 mmHg increase in DBP	1	0.99 (0.96 - 1.03)	/	/	0.576	
**Duration of DM**	2	1.00 (0.99 - 1.01)	0.229	30.9	0.805	Fixed
**MetS**	2	2.29 (1.55 - 3.38)	0.545	0	<0.001	
**TyG**						Fixed
High TyG	2	1.69 (1.49 – 1.92)	0.076	68.2	<0.001	
Per 1 unit increase in TyG	3	1.47 (1.21 – 1.78)	0.228	32.4	0.003	
**BUN**						Random
High BUN	1	1.42 (1.16 - 1.74)	/	/	0.001	
Per 1 mmol/L increase in BUN	2	1.20 (1.00 - 1.46)	0.018	82.2	0.056	
**Smoke**	6	0.95 (0.67 - 1.34)	<0.001	81	0.778	Random
**Alcohol**	6	1.47 (1.06 - 2.03)	0.001	76.1	0.02	Random
**HbA1c**						Fixed
HbA1c ≥7	1	0.94 (0.89 - 0.99)	/	/	0.014	
Per 1% increase in HbA1c	4	0.92 (0.89 - 0.96)	0.517	0	<0.001	
**Crea**						Random
Per 1mg/dL increase in Crea	2	1.91 (0.91 - 4.03)	<0.001	92.6	0.088	

OR, odds ratio; CI, confidence interval; eGFR, estimated glomerular filtration rate; HC, hip circumference; SBP, systolic blood pressure; DBP, diastolic blood pressure; DM, diabetes mellitus; MetS, metabolic syndrome; TyG, triglyceride-glucose index; BUN, blood urea nitrogen; HbA1c, glycated hemoglobin; Crea, creatinine; mg/dL.Bold values indicate the overarching risk factor category. Beneath each bolded category, the indented rows present specific effect estimates from different measurement dimensions (categorical, continuous, or different threshold definitions). Bold formatting does not indicate statistical significance.

#### 3.4.1 Sex

With regard to sex, a meta-analysis of 9 studies showed that females had a slightly higher risk of HUA than males, but the difference was not statistically significant (OR = 1.63, 95% CI: 0.87-3.04, P = 0.124) (**Supplementary Figure 33**). Due to high heterogeneity (I² = 97.5), we performed a subgroup analysis based on diagnostic criteria. The results showed that in the subgroup using sex-specific criteria (M > 7.0mg/dL, F > 6.0 mg/dL), female was a significant risk factor (OR = 2.26, 95% CI: 1.44-3.53, P < 0.001), whereas in the subgroup using a unified criterion (>7.0 mg/dL), females were found to be a protective factor (OR = 0.55, 95% CI: 0.26-1.14, P = 0.106). This indicates that diagnostic criteria are the primary driver of sex-related heterogeneity, as the more stringent cutoff for females (>6.0 mg/dL) increases the likelihood of HUA classification despite the urate-lowering effect of estrogen.

#### 3.4.2 Age

Regarding age, a meta-analysis of 5 studies that treated age as a continuous variable showed that the risk of HUA decreases with each additional year of age (OR = 0.98, 95% CI: 0.96-0.99, P = 0.013) (**Supplementary Figure 34**). However, due to high heterogeneity (I² = 86.8%), this suggests that the relationship between age and HUA may not follow a simple linear pattern. We further listed the comparative analyses of different age groups from some studies, for example, one study showed that the risk of HUA in patients >45 years old was 1.90 times that of patients <45 years old (OR = 1.9, 95% CI: 1.16-3.10, P = 0.01).

#### 3.4.3 Renal function

With regard to renal function, compared with patients with normal renal function, those with an eGFR < 60 mL/min/1.73 m² had a 2.75-fold increased risk of developing HUA (OR = 3.75, 95% CI: 2.36-5.95, P < 0.001) (**Supplementary Figure 53**). Analysis of continuous variables showed that for every 1 mL/min/1.73 m² increase in eGFR, the risk of HUA decreased by 2%. Elevated blood urea nitrogen (BUN) was significantly positively associated with the risk of HUA (OR = 1.42, P = 0.001) (**Supplementary Figure 49**). For every 1 mg/dL increase in serum creatinine (Crea), the risk of HUA showed an upward trend (OR = 1.91, P = 0.088) (**Supplementary Figure 54**). Thus, impaired renal function is a risk factor for HUA.

#### 3.4.4 Obesity

Regarding BMI, five studies reported the risk of HUA in overweight patients. The results showed that, compared with the normal BMI group, overweight patients had a 75% increased risk of developing HUA (OR = 1.75, 95% CI: 1.08-2.86, P < 0.001) (**Supplementary Figure 35**). Although moderate-to-high statistical heterogeneity was present in this subgroup (I² = 75.1%, P = 0.003), the direction of effect was consistent across all studies, with all reporting a positive association. The pooled results of three studies indicated that the risk of HUA in obese patients was 2.23 times higher than in those with normal BMI (OR = 2.23, 95% CI: 1.34-3.70, P = 0.002), and the heterogeneity in the obesity subgroup was relatively low (I² = 53.3%, P = 0.118), suggesting that the pathogenic effect of obesity on HUA is consistent across different study populations. Eight studies examined the association between BMI and HUA using BMI as a continuous variable. The results showed that for every 1 kg/m² increase in BMI, the risk of HUA in patients with T2DM increased significantly by 8% (OR = 1.08, 95% CI: 1.05-1.11, P < 0.001), which was statistically significant, and heterogeneity was moderate (I² = 47.9%, P = 0.062). Thus, elevated BMI is a risk factor for HUA.

In addition to the general obesity indicator (BMI), indicators of central obesity--waist circumference (WC) and hip circumference (HC), also showed a strong association with the risk of HUA. A meta-analysis of four studies showed that patients with High WC had a 2.25-fold higher risk of developing HUA compared to those with low WC (OR = 2.25, 95% CI: 1.33-3.81, P = 0.002) (**Supplementary Figure 40**). Four studies reported the risk of HUA with WC as a continuous variable, the results showed that for every 1 cm increase in WC, the risk of HUA increased by 3% (OR = 1.03, 95% CI: 1.01-1.04, P < 0.001), and there was very low heterogeneity among the studies (I² = 0%). Similarly, for every 1 cm increase in HC, the risk of HUA increased by 3% (OR = 1.03, 95% CI: 1.02-1.05, P < 0.001, I² = 0%) (**Supplementary Figure 42**). These findings indicate that increases in both WC and HC are significant independent risk factors for HUA in patients with T2DM.

#### 3.4.5 Lipid profile

Regarding dyslipidemia, three studies reported the association between hypertriglyceridemia and the risk of HUA. The results showed an OR of 1.38 (95% CI: 1.00-1.92, P = 0.053), and for every 1 mmol/L increase in TG, the risk increased significantly by 16% (OR = 1.16, 95% CI: 1.06-1.26, P = 0.001, 6 studies) (**Supplementary Figure 36**). Similarly, patients in the low HDL-C group had a 27% increased risk of HUA (OR = 1.27, 95% CI: 1.16-1.38, I² = 0%, P < 0.001, 4 studies), and for every 1 mmol/L decrease in HDL-C levels, the risk of HUA increased by 97% (OR = 1.97, 95% CI: 1.11-3.50, I² = 75.5%, P = 0.020, 4 studies) (**Supplementary Figure 37**). In contrast, high LDL-C was only weakly associated with HUA risk (OR = 1.10, 95% CI: 1.00-1.20, P = 0.044, 2 studies), and for every 1 mmol/L increase in LDL-C (OR = 1.18, 95% CI: 0.87-1.60, P = 0.297, 2 studies) (**Supplementary Figure 39**). High TC (OR = 1.18, 95% CI: 0.83-1.68, P = 0.351, 2 studies) and a 1 mmol/L increase in TC (OR = 0.97, 95% CI: 0.70-1.36, P = 0.865, 2 studies) were not statistically significant (**Supplementary Figure 38**). These findings suggest that, in patients with T2DM, elevated TG and reduced HDL-C are the lipid metabolism abnormalities most closely associated with HUA.

#### 3.4.6 Other clinical characteristics

Concomitant hypertension significantly increased the risk of HUA in patients with T2DM (OR = 1.84, 95% CI: 1.08-3.12, P = 0.025, 6 studies) (**Supplementary Figure 43**), with a more pronounced risk effect observed in the high systolic blood pressure (High SBP) group (OR = 2.79, 95% CI: 1.09-7.16, P = 0.033, 2 studies) (**Supplementary Figure 44**).

Regarding metabolic disorders, patients with MetS had a 2.29-fold higher risk of developing HUA compared to those without (95% CI: 1.55-3.38, P < 0.001, 2 studies) (**Supplementary Figure 47**). For every one-unit increase in the TyG index, the risk of HUA increased by 59% (OR = 1.47, 95% CI: 1.21-1.78, P = 0.003, 3 studies) (**Supplementary Figure 48**). Thus, metabolic disorders and insulin resistance are also risk factors for HUA.

Among lifestyle factors, alcohol consumption was a significant risk factor for HUA in patients with T2DM (OR = 1.47, 95% CI: 1.06-2.03, P = 0.020, 6 studies) (**Supplementary Figure 51**). In contrast, smoking (OR = 0.95, 95% CI: 0.67-1.34, P = 0.778, 6 studies) did not show statistical significance (**Supplementary Figure 50**).

Regarding diabetes-specific characteristics, each additional year of diabetes duration (OR = 1.00, 95% CI: 0.99-1.01, P = 0.941, 2 studies) was not significantly associated with HUA (**Supplementary Figure 46**). HbA1c levels were negatively associated with the incidence of HUA, for every 1% increase in HbA1c, the risk of HUA decreased by 8% (OR = 0.92, 95% CI: 0.89-0.96, P < 0.001, I² = 0%, 4 studies) (**Supplementary Figure 52**).

#### 3.4.7 Sensitivity analysis of risk factors for HUA

Leave-one-out sensitivity analyses showed that the pooled results for most risk factors were robust. However, the estimates for hypertension and alcohol consumption were sensitive to individual studies. For hypertension, excluding Woldeamlak 2019 reduced the pooled OR from 1.84 to 1.27, and excluding Peng 2025 rendered the result non-significant. For alcohol consumption, excluding Arersa 2020 or Choukem 2016 resulted in a loss of statistical significance. These findings indicate that, although the direction of association was consistently positive for both hypertension and alcohol consumption, the pooled estimates are not fully robust and require further validation in future studies.

### 3.5 Analysis of risk factors for T2DM complicated by gout

Four studies reported risk factors for gout in patients with T2DM. We conducted a meta-summary of combinable variables and summarized variables reported in individual studies (**Supplementary Table 9**). The summary results showed that sex was a significant risk factor for gout. Male patients had a significantly higher risk of developing gout than females (OR = 2.75, 95% CI: 1.36-5.55, P<0.001, 2 studies) (**Supplementary Figure 55**). In contrast, hypertriglyceridemia did not show statistical significance in the current meta-analysis (OR = 1.45, 95% CI: 0.89-2.37, P = 0.135, 2 studies) (**Supplementary Figure 56**).

## 4 Discussion

### 4.1 Main findings

This systematic review and meta-analysis, encompassing 87 studies and 977,573 patients with T2DM, provides the first comprehensive global assessment of the prevalence of HUA and gout and their associated risk factors in this population. Regarding prevalence, the prevalence of HUA and gout among T2DM patients was 22.0% and 6.0%, respectively. Subgroup analyses and meta-regression revealed significant geographical variations in HUA prevalence, with the highest rates observed in Africa and North America and lowest in South America. Regarding risk factors, impaired renal function, obesity, hypertension, dyslipidemia, MetS, the TyG index, and alcohol consumption were identified as major risk factors for HUA, while elevated HbA1c was associated with a reduced risk of HUA. Additionally, male sex was identified as a risk factor for concomitant gout.

### 4.2 Interpretation of results and discussion of mechanisms

#### 4.2.1 HUA and gout prevalence

According to the latest global modeling study by Ngandeu-Singweand and colleagues, the global prevalence of HUA in 2023 was 11.2% in adult females and 18.6% in adult males (109). In the present study, the pooled point estimate of HUA prevalence in patients with T2DM was 22.0%, which is higher than that in the general population. However, given the substantial between-study heterogeneity, the 95% PI was wide (4.9–39.2%), indicating that the pooled estimate of 22.0% reflects an overall trend of a greater HUA burden in patients with T2DM rather than a precise, universally applicable figure. Similarly, the pooled prevalence of gout was 6.0% (95% CI: 4.4–7.5%), which is higher than the 0.7% reported for the global general population (110), although this finding is also subject to high heterogeneity. A previous meta-analysis by Jiang and colleagues reported that the prevalence of DM in patients with HUA and gout was 19.10% and 16.70%, respectively, significantly exceeding that in the general population (12). The present findings further support the existence of a bidirectional relationship between T2DM and HUA/gout.

Subgroup analysis and meta-regression identified geographic region as a significant source of heterogeneity. HUA prevalence was higher in Africa and North America and lower in South America. The pooled prevalence in Africa (29.0%) was consistent with that of a previous meta-analysis of T2DM patients in Africa (27.28%) (13). The high prevalence in Africa may be attributable to the high burden of hypertension and kidney disease in the region (111, 112). Notably, the prevalence estimates for North America (28.6%) and South America (17.3%) were each based on only two studies, and therefore require validation through larger-scale investigations.

Diagnostic criteria represent another noteworthy source of heterogeneity. The definition of HUA varied considerably across the included studies, falling broadly into three categories: sex-specific criteria: M >7.0/F >6.0 mg/dL, or M >420/F >360 μmol/L (5), a uniform threshold of >7.0 mg/dL (or 420 μmol/L) irrespective of sex (113), and the >6.8 mg/dL threshold based on the physiological solubility of urate (114, 115). Subgroup analysis showed that the pooled prevalence under the two main criteria were similar (22.5% vs. 19.3%), with substantial overlap in their 95% CIs, and meta-regression did not identify diagnostic criteria as a significant source of heterogeneity (p = 0.364). Nevertheless, the choice of diagnostic threshold had a pronounced impact on prevalence estimates in females. The pooled HUA prevalence was 22.2% in males and 23.1% in females—comparable point estimates—yet the 95% PI for the female subgroup was wide (0–51.5%). Further analysis revealed that in females, the prevalence was 25.6% when the >6.0 mg/dL threshold was applied versus only 14.3% under the >7.0 mg/dL threshold. The risk factor analysis similarly showed that female sex was a risk factor for HUA under sex-specific criteria, whereas it appeared protective under the uniform >7.0 mg/dL criterion. This pattern differs from the sex distribution of HUA in the general population, where HUA prevalence is consistently higher in males than in females (109), and may be explained by the older age of patients with T2DM and the postmenopausal rise in SUA due to declining estrogen levels. In contrast, the risk of gout was significantly higher in males, consistent with previous reports. Collectively, these findings underscore the substantial influence of diagnostic criteria on sex-specific HUA prevalence estimates, highlighting the need for global standardization of HUA diagnostic criteria in future research.

#### 4.2.2 Risk factors

The relationship between age and HUA was complex in the present study, with inconsistent results between continuous and categorical variable analyses, which may be attributable to the nonlinear association between age and uric acid levels, as well as confounding factors such as renal function.

Obesity, central obesity, dyslipidemia (elevated TG and low HDL-C), hypertension, and MetS were all risk factors for HUA in patients with T2DM, and these factors represent core components of the MetS. Insulin resistance serves as the key link connecting these factors with T2DM and HUA: hyperinsulinemia upregulates URAT1 and GLUT9 while inhibiting ABCG2 and OAT1, thereby reducing renal uric acid excretion (116, 117). Furthermore, uric acid itself can activate the RAAS, promoting blood pressure elevation and endothelial dysfunction, thus creating a mutually reinforcing vicious cycle (118). Previous studies have confirmed the close association between MetS components and HUA (119–121), and the present study further validates these associations specifically in a T2DM population. In addition, the TyG index, a convenient surrogate marker of insulin resistance, was identified as an independent risk factor for HUA in this study.

Impaired renal function was another significant risk factor: patients with eGFR <60 had a 2.75-fold higher risk of HUA than those with eGFR ≥60, highlighting the importance of enhanced uric acid monitoring in patients with T2DM with concomitant renal insufficiency. HUA prevalence reached 48.1% among T2DM patients using diuretics. Diuretics, particularly thiazides, increase renal tubular uric acid reabsorption. Accordingly, antihypertensive agents with minimal impact on uric acid metabolism should be prioritized for T2DM patients with HUA.

Notably, HbA1c levels were inversely associated with HUA risk, indicating that patients with poorer glycemic control had a lower risk of HUA. This inverse relationship between SUA and HbA1c in patients with T2DM, contrasting with the positive correlation observed in healthy controls, has been reported previously (57). It may be explained by osmotic diuresis under hyperglycemic conditions promoting uric acid excretion (122), or by the uricosuric effect of medications such as SGLT2 inhibitors. However, this phenomenon should not be interpreted as “poorer glycemic control being beneficial for HUA”, given the well-established detrimental effects of hyperglycemia. Patients with T2DM should maintain adequate glycemic control while also attending to uric acid management. Future prospective cohort studies are needed to elucidate the causal relationship between HbA1c and SUA.

### 4.3 Clinical implications and future directions

The American Diabetes Association (ADA) 2026 Standards of Care identifies gout as a key comorbidity requiring assessment in patients with diabetes, and emphasize that diabetes management must shift from a narrow focus on glycemic control toward a patient-centered comprehensive evaluation (2). The present study further reveals the widespread burden of these comorbidities in patients with T2DM, providing epidemiological evidence to support the inclusion of uric acid assessment in the comprehensive medical evaluation for diabetes. In clinical practice, HUA risk stratification can be performed for T2DM patients with concomitant renal insufficiency, MetS, or dyslipidemia, with regular monitoring of SUA. For patients at high risk of HUA, thiazide diuretics should be avoided whenever possible in antihypertensive therapy, and SGLT2 inhibitors, which possess the dual benefits of glucose lowering and urate reduction, may be the preferred option (123–125). Furthermore, a comprehensive metabolic management strategy targeting body weight, blood pressure, and lipid profiles is equally important for T2DM patients with multiple metabolic comorbidities.

### 4.4 Strengths and limitations

This is the first meta-analysis to systematically evaluate the global prevalence of comorbid HUA and gout and their associated risk factors in patients with T2DM. With 87 studies included, covering six continents and encompassing nearly 980,000 patients, the study has good representativeness. We systematically explored sources of heterogeneity, reported prevalence estimates stratified by subgroup, and identified key risk factors.

Several limitations should be acknowledged. First, substantial between-study heterogeneity was present, and although subgroup analyses and meta-regression were performed to explore its sources, a considerable proportion remained unexplained. However, high I² values are almost inevitable in prevalence meta-analyses, as such, the pooled prevalence estimates should be interpreted as a summary of the central tendency of the available evidence rather than as precise global estimates. The wide 95% PIs reported in this study further reflect a cautious approach to this issue. Second, for HUA prevalence, the number of included studies from South America and North America was limited, and data from Oceania were lacking. Given the documented high burden of HUA among Pacific Islander populations (109), this geographic gap limits the generalizability of our findings. Third, the lack of uniformity in HUA diagnostic criteria may have introduced potential bias, even though meta-regression did not identify diagnostic criteria as a significant source of heterogeneity. Fourth, the limited number of studies on gout precluded more in-depth investigation of this condition.

## 5 Conclusions

This systematic review and meta-analysis found that the combined prevalence of HUA and gout in patients with T2DM was 22.0% (95% CI: 20.1%-24.0%, 95% PI: 4.9-39.2%) and 6.0% (95% CI: 4.4–7.5%), respectively, which is significantly higher than in the general population. Impaired renal function, obesity, dyslipidemia, hypertension, MetS, elevated TyG index, and alcohol consumption are risk factors for HUA, while male sex is a risk factor for gout. These findings provide evidence-based guidance for clinical practice. These results suggest that serum uric acid levels should be monitored regularly in patients with T2DM, and greater emphasis should be placed on comprehensive metabolic management, particularly for those with risk factors for HUA or gout.

## Data Availability

The data analyzed in this study is subject to the following licenses/restrictions: The data analyzed in this study were extracted from 87 previously published articles. These primary studies are publicly available via PubMed, Embase, Web of Science, and the Cochrane Library. However, the individual-level raw data from those studies are not publicly available because they are owned by the original study investigators. Requests to access these datasets should be directed to wanghailong@bucm.edu.cn.
