# Supplementary material for: Prevalence and risk factors of hyperuricemia and gout in patients with type 2 diabetes mellitus: a systematic review and meta-analysis
Source: Front Endocrinol (Lausanne). 2026 Jun 24;17:1857217. doi: 10.3389/fendo.2026.1857217 (PMC13341555; doi:10.3389/fendo.2026.1857217)
Supplement: Supplementary file 1 [file DataSheet1.docx]

**Supplementary Appendix**

**Search Strategy** **....................................................................................................................... 2**

**Table S1.** Characteristics of the included studies of HUA**........................................................ 4**

**Table S2.** Characteristics of the included studies of Gout**........................................................ 12**

**References included in this study........................................................................................... 14**

**Table S3.** The quality assessment of cross-sectional studies**................................................... 26**

**Table S4.** The quality assessment of cohort studies**................................................................. 31**

**Table S5-6.** Sex Subgroup Analysis Results of HUA prevalence**............................................ 32**

**Table S7.** Meta-regression Results of HUA prevalence**........................................................... 34**

**Table S8.** Summary of Non-pooled Risk Factors of HUA**....................................................... 35**

**Table S9.** Summary of Pooled Risk Factors of Gout**............................................................... 38**

**Table S10.** Summary of Non-pooled Risk Factors of Gout**..................................................... 39**

**Figure** **S1**. Funnel plot of overall HUA prevalence**................................................................. 40**

**Figure** **S2.** Egger's test plot for overall HUA prevalence**........................................................ 41**

**Figure S3.** Bubble plot of meta-regression of HUA Prevalence**............................................. 41**

**Figure S4-21** Subgroup Forest plot for HUA prevalence in T2DM**........................................ 43**

**Figure S22.** Forest plot of overall Gout prevalence**................................................................ 58**

**Figure S23.** Funnel plot of overall Gout prevalence**............................................................... 58**

**Figure S24.** Publication bias assessment for Gout prevalence**............................................... 59**

**Figure S25.** Sensitivity analysis of overall Gout prevalence**.................................................. 59**

**Figure S26-32.** Subgroup Forest plot for Gout prevalence in T2DM**..................................... 60**

**Figure S33-54.** Forest plot, Sensitivity analysis and funnel plot of risk factors for HUA**...... 64**

**Figure S55-56.** Forest plot, Sensitivity analysis and funnel plot of risk factors for Gout**....... 81**

**Search Strategy**

Pubmed:

((Diabetes Mellitus, Type 2[MeSH Terms]) OR (Diabetes Mellitus, Type 2[Title/Abstract]) OR (Type 2 Diabetes Mellitus[Title/Abstract]) OR (Type 2 Diabetes[Title/Abstract]) OR (T2DM[Title/Abstract]) OR (T2D[Title/Abstract])) AND (((((Gout[MeSH Terms]) OR (Gout[Title/Abstract])) OR (Arthritis, Gouty[Title/Abstract])) OR (Arthritides, Gouty[Title/Abstract])) OR (Gouty Arthritides[Title/Abstract]) OR (((((((((((((hyperuricemia[MeSH Terms]) OR (hyperuricemia[Title/Abstract])) OR (uric acid[MeSH Terms])) OR (uric acid[Title/Abstract])) OR (Acid, Uric[Title/Abstract])) OR (Monosodium Urate[Title/Abstract])) OR (Urate, Monosodium[Title/Abstract])) OR (Sodium Acid Urate[Title/Abstract])) OR (Acid Urate, Sodium[Title/Abstract])) OR (Urate, Sodium Acid[Title/Abstract])) OR (Sodium Acid Urate Monohydrate[Title/Abstract])) OR (Sodium Urate[Title/Abstract])) OR (Urate, Sodium[Title/Abstract])) OR (Urate[Title/Abstract]))

Web of science:

(TS=(Diabetes Mellitus, Type 2 OR Type 2 Diabetes Mellitus OR Type 2 Diabetes OR T2DM OR T2D)) AND (TS=(Gout OR Arthritis, Gouty OR Arthritides, Gouty OR Gouty Arthritides OR hyperuricemia OR uric acid OR Acid, Uric OR Monosodium Urate OR Urate, Monosodium OR Sodium Acid Urate OR Acid Urate, Sodium OR Urate, Sodium Acid OR Sodium Acid Urate Monohydrate OR Sodium Urate OR Urate, Sodium OR Urate))

Embse:

('diabetes mellitus type 2'/exp OR 'diabetes mellitus type 2':ab,ti OR 'type 2 diabetes mellitus':ab,ti OR 'type 2 diabetes':ab,ti OR 't2dm':ab,ti OR 't2d':ab,ti)

AND

('Gout'/exp OR 'Gout':ab,ti OR 'arthritis Gouty':ab,ti OR 'arthritides Gouty':ab,ti OR 'Gouty arthritides':ab,ti OR 'hyperuricemia'/exp OR 'hyperuricemia':ab,ti OR 'uric acid'/exp OR 'uric acid':ab,ti OR 'acid uric':ab,ti OR 'monosodium urate':ab,ti OR 'urate monosodium':ab,ti OR 'sodium acid urate':ab,ti OR 'acid urate sodium':ab,ti OR 'urate sodium acid':ab,ti OR 'sodium acid urate monohydrate':ab,ti OR 'sodium urate':ab,ti OR 'urate sodium':ab,ti OR 'urate':ab,ti)

Cochrane Library:

#1 Diabetes Mellitus, Type 2 78699

#2 Type 2 Diabetes Mellitus 78699

#3 Type 2 Diabetes 103326

#4 T2DM 10153

#5 T2D 6243

#6 #1 OR #2 OR #3 OR #4 OR #5 103845

#7 Gout 2416

#8 Gouty Arthritis 474

#9 Gouty Arthritides 0

#10 hyperuricemia 1698

#11 uric acid 6298

#12 Monosodium Urate 84

#13 Sodium Acid Urate 65

#14 Sodium Acid Urate Monohydrate 1

#15 Sodium Urate 106

#16 Urate 1205

#17 #7 OR #8 OR #9 OR #10 3581

#18 #11 OR #12 OR #13 OR #14 6328

#19 #15 OR #16 OR #17 OR #18 8106

#20 #6 AND #19 1262

**Table S1.** Characteristics of the included studies of HUA

| Author (Year) | Country / Region | Study Design | Sample Size (N) | HUA (N) | HUA Prevalence | Age (Mean/SD) | Male n (%) | HUA Definition | Reported Risk Factors |
| --- | --- | --- | --- | --- | --- | --- | --- | --- | --- |
| Abdel 2024^1^ | Tanzania / Africa | CSS | 360 | 160 | 44.4% | 61 (57-68) | 40% | M > 450, F > 360 μmol/L | Gender, BMI, eGFR < 60 (CKD) |
| Abudureyimu 2023^2^ | China / Asia | CSS | 618 | 78 | 12.6% | 56.42 ± 12.075 | 66% | M ≥ 420, F ≥ 360 μmol/L | Sex, Ethnicity, ALB, CysC, IPHOS, ALP, CO2, HDL-C |
| Abujbara 2022^3^ | Jordan / Asia | CSS | 655 | 184 | 28.1% | ＞60 years (40.8%) | 47% | M > 7.0, F > 6.0 mg/dL | Gender, ACE and ARBs, β-blockers,  Family history of HUA, WC |
| Adotey 2025^4^ | China / Asia | CSS | 2268 | 315 | 13.9% | 56 (47-63) | 62% | > 420 μmol/L | / |
| Akande 2006^5^ | Nigeria / Africa | CSS | 121 | 13 | 10.7% | 57.3 ± 10 | 36% | ＞380 μmol/L |  |
| Andrade 2014^6^ | Brazil / South America | CSS | 123 | 14 | 11.4% | 50 ± 9 | 0% | M ≥ 7.0, F ≥ 6.0 mg/dL | / |
| Arersa 2020^7^ | Ethiopia / Africa | CSS | 287 | 63 | 22.0% | 51.79 ± 14.35 | 66% | M > 7.2, F > 6.0 mg/dL | Age, BMI, Duration of DM, Famliy History of CVD, SBP, DBP, Cigarette smoking, Alcohol drinking, WHR, Chat chewing |
| Barry 2021^8^ | Senegal / Africa | CSS | 153 | 45 | 29.4% | 56.63 (range: 21-87) | 35% | M > 416, F > 357 μmol/L |  |
| Bo 2001^9^ | Italy / Europe | CSS | 1186 | 143 | 12.1% | T1: 63.9 ± 11.4;  T2: 64.3 ± 10.6;  T3: 66.0 ± 11.6 | / | ＞ 416.4 μmol/L | / |
| Chen 2024 a^10^ | China / Asia | CSS | 8243 | 3766 | 45.7% | ≥65 years (73.0%) | 44% | M > 420, F > 360 μmol/L | Gender, WC, Diabetes medication use, DBP, GGT, BUN, TG, LDL-C, HDL-C, FPG, eGFR |
| Chen 2024 b^11^ | China / Asia | CSS | 1756 | 292 | 16.6% | 57.23 ± 10.15 | 50% | > 420 μmol/L | / |
| Choukem 2016^12^ | Cameroon / Africa | CSS | 438 | 167 | 38.1% | 56.3 ± 10.5 | 56% | M > 7.0, F > 6.0 mg/dL | History of DR, Alcohol, Smoking, BMI≥25, eGFR<60, Hydrochlorothiazide treatment, Statin treatment |
| Deng 2016^13^ | China / Asia | PCS | 8274 | 1069 | 12.9% | 61.7 (53.1–71.2) | 51% | > 7.0 mg/dL | / |
| Donkeng 2021^14^ | Cameroon / Africa | CSS | 80 | 22 | 27.5% | 61.22 ± 12.27 | 61% | M > 7.0, F > 6.0 mg/dL | / |
| Eljaaly 2021^15^ | Saudi Arabia / Asia | CSS | 433 | 109 | 25.2% | 55.4 ± 6.2 | 44% | M > 7.0, F > 6.0 mg/dL | Sex, Hip circumference (HC), TC, HDL-C, TG, Serum creatinine |
| Fayazi 2023^16^ | Iran / Asia | CSS | 230 | 61 | 26.5% | 57.36 ± 8.05 | 30% | ≥ 6.8 mg/dL | / |
| Fennoun 2020^17^ | Morocco / Africa | CSS | 190 | 50 | 26.3% | 53.9 (range: 27-80) | 24% | M > 7.0, F > 6.0 mg/dL | / |
| Fouad 2016^18^ | Egypt / Africa | CSS | 736 | 235 | 31.9% | 47.9 ± 11.8 | 66% | ≥ 7.0 mg/dL | / |
| Gao 2019^19^ | China / Asia | CSS | 435 | 90 | 20.7% | NUA (n = 345): 54.21 ± 10.21；Synthesis-increased HUA: 51.76 ± 10.84;  Mixed type of HUA: 49.67 ± 8.70;  Excretion-decreased HUA: 53.14 ± 10.74 | 56% | M > 420, F > 360 μmol/L | synthesis-increased hyperuricemia: HbA1c, eGFR, TBIL, TG, HDL-C; mixed type of hyperuricemia: TG; excretion-decreased hyperuricemia: BMI, HDL-C* |
| Gobusamang 2019^20^ | Botswana / Africa | CSS | 334 | 92 | 27.5% | HUA: 58±11; Non-HUA: 56±14 | 29% | > 400 μmol/L |  |
| Guo 2015^21^ | China / Asia | CSS | 20207 | 4151 | 20.5% | 71 ± 7 | 42% | M > 420, F > 360 μmol/L | / |
| Han 2017^22^ | China / Asia | CSS | 4625 | 590 | 12.8% | With SRC: 64.43 ± 10.40; Without SRC: 59.65 ± 11.74 | 60% | M > 420 μmol/L | / |
| Hermans 2009^23^ | Belgium / Europe | CSS | 705 | 229 | 32.5% | HUA: 68 ± 10; Non-HUA: 66 ± 12 | 100% | M ≥ 7.0 mg/dL | / |
| Hu 2021^24^ | China / Asia | CSS | 3481 | 661 | 19.0% | VTDR: 59.1 ± 9.1; Non-VTDR: 56.7 ± 11.3 | 57% | M ≥ 420, F ≥ 360 μmol/L | / |
| Huang 2025^25^ | China / Asia | CSS | 1261 | 300 | 23.8% | 59 (48-68) | 56% | ≥ 420 μmol/L | MHR |
| Kim 2012^26^ | Korea / Asia | CSS | 699 | 95 | 13.6% | 55 ± 14 | 44% | M > 7.0, F > 6.0 mg/dL | Age, Gender (Male), SBP, DBP, eGFR<60, TG, VFA |
| Kim 2026^27^ | Korea / Asia | CSS | 4575 | 460 | 10.1% | 61.0 ± 4.6 | 56% | ≥ 7.0 mg/dL | Diabetes duration |
| Li 2018^28^ | China / Asia | CSS | 609 | 98 | 16.1% | Male: 54.9 ± 11.0; Female: 58.7 ± 10.1 | 49% | M ≥ 420, F ≥ 360 μmol/L | Male: BMI, BUN, 24-hour urinary protein;  Female: BM, BUN, TG |
| Liang 2016^29^ | China / Asia | CSS | 385 | 93 | 24.2% | 64.6 ± 12.1 | 50% | ≥ 7.0 mg/dL | / |
| Lin 2024^30^ | China / Asia | CSS | 2956 | 822 | 27.8% | 58.9 ± 15.2 | 59% | M > 420, F > 360 μmol/L | Remnant cholesterol (RC) |
| Liu 2022^31^ | China / Asia | CSS | 345 | 56 | 16.2% | 54.27 ± 11.34 | 53% | ≥ 420 μmol/L | C-peptide releasing parameters* |
| Liu 2025^32^ | China / Asia | CSS | 2352 | 336 | 14.3% | 57.3 ± 13.2 | 41% | M > 420, F > 360 μmol/L | log eGDR, Age, eGFR, AST, UACR, Hb |
| Lopes 2023^33^ | Portugal / Europe | CSS | 420 | 72 | 17.1% | 80.59 ± 7.92 | 49% | Clinical Diagnosis | / |
| Lu 2023^34^ | China / Asia | CSS | 1447 | 279 | 19.3% | 63.5 (58.0-69.0) | 100% | M ＞ 7.0 mg/dL | WC, HC, WHR, WHtR |
| Luo 2022^35^ | China / Asia | CSS | 719 | 87 | 12.1% | 58 (53–67） | 61% | M > 420, F > 360 μmol/L | TyG |
| Mantovani 2016^36^ | Italy / Europe | CSS | 842 | 243 | 28.9% | With AF: 75 ± 9; Without AF: 65 ± 13 | 55% | M > 7.0, F > 6.0 mg/dL, or ULT | / |
| Mantovani 2018^37^ | Italy / Europe | CSS | 245 | 59 | 24.1% | 72 ± 9 | 57% | M > 7.0, F > 6.0 mg/dL, or ULT | / |
| Mirghani 2018^38^ | Sudan / Africa | CSS | 170 | 26 | 15.3% | 59.62 ± 9.91 | 52% | M ≥ 6.0, F ≥ 5.5 mg/dL |  |
| Mundhe 2016^39^ | India / Asia | CSS | 150 | 38 | 25.3% | Male: 57.06 ± 9.27; Female: 56.29 ± 9.35 | 59% | M > 7.0, F > 6.0 mg/dL | / |
| Ndang Ngou Milama 2022^40^ | Gabon / Africa | CSS | 333 | 96 | 28.8% | 56.6 ± 9.8 | 100% | Clinical Diagnosis | / |
| Ogbera 2010^41^ | Nigeria / Africa | CSS | 601 | 150 | 25.0% | 59.9 ± 10.3 | 45% | M > 7.0, F > 6.0 mg/dL | Age, BMI, WC, Sex, Alcohol history, Smoking history, HDL-C, TG,Duration of DM, HbA1c* |
| Paduraru 2025^42^ | Romania / Europe | CSS | 304 | 75 | 24.7% | 66.4 ± 11.0 | / | M > 7.0, F > 6.0 mg/dL | / |
| Panero 2012^43^ | Italy / Europe | PCS | 1509 | 199 | 13.2% | 68.8 ± 10.6 | 44% | > 416.36 μmol/L | / |
| Patel 2024^44^ | India / Asia | CSS | 580 | 110 | 19.0% | controls (M: 55±12, F: 50±15); T2DM (M: 57±11, F: 51±20) | 73% | M > 7.0, F > 6.0 mg/dL | / |
| Peng 2025^45^ | China / Asia | CSS | 1768 | 291 | 16.5% | HUA: 57(45, 64); Non-HUA: 59(51, 65) | 63% | M > 420, F > 360 μmol/L | WBC, Sex, Age, BMI, Hypertension, TG, HDL-C, LDL-C, Crea |
| Peralta-Vera 2025^46^ | Peru / South America | CSS | 815 | 185 | 22.7% | 57 ± 12.3 | 63% | ≥ 6 mg/dL | Abdominal Obesity, Sex, HbA1c, eGFR, SBP, DBP* |
| Qin 2021^47^ | China / Asia | CSS | 635 | 98 | 15.4% | 51.7 (50.8–52.7) | 63% | M > 416, F > 357 μmol/L | Urinary excretion rate of glucose (UEGL) |
| Qin 2025^48^ | China / Asia | CSS | 460 | 154 | 33.5% | 61.283 ± 7.777 | 100% | M > 420 μmol/L | / |
| Rafiullah 2020^49^ | Saudi Arabia / Asia | CSS | 198 | 31 | 15.7% | 57.20 ± 11.25 | 54% | M > 7.06, F > 6.05 mg/dL | / |
| Shang 2024^50^ | China / Asia | CSS | 714 | 64 | 9.0% | Good Glycemic Control: 61 (53, 68); Poor Glycemic Control: 58 (50, 66) | 52% | Clinical Diagnosis | / |
| Sheikhbahaei 2014^51^ | Iran / Asia | CSS | 1463 | 226 | 15.4% | Range by SUA quartiles: 54.4 ± 10.2 to 57.2 ± 11.2 | 45% | M ≥ 7.0, F ≥ 6.5 mg/dL | Mets, Central obesity, High blood pressure, TG, HDL-C, HOMA > 4.32, Microalbuminuria, Macroalbuminuria, CKD |
| Shi 2020^52^ | China / Asia | CSS | 2899 | 469 | 16.2% | 66.0 (60.0, 70.0) | 49% | M ≥ 420, F ≥ 360 μmol/L | CHD group: HbA1c, TG, BUN, SCR; DN group: BMI, HbA1c, TC, TG, BUN, SCR, UMA* |
| Shou 2020^53^ | China / Asia | CSS | 1476 | 737 | 49.9% | PAD: 72.5 ± 5.9: Non-PAD: 66.3 ± 4.8 | 43% | Clinical Diagnosis | / |
| Singh 2023^54^ | India / Asia | CSS | 312 | 39 | 12.5% | 47.03 ± 10.08 | 70% | M ≥ 7.0, F ≥ 6.0 mg/dL | / |
| Sun 2025^55^ | China / Asia | CSS | 996 | / | / | 60.39 ± 13.34 | 55% | > 420 μmol/L | TyG |
| Tanaka 2011^56^ | Japan / Asia | CSS | 1493 | 354 | 23.7% | 64.9 ± 10.1 | 83% | M ≥ 7.0, F ≥ 6.0 mg/dL, or ULT | / |
| Tang 2025 a^57^ | China / Asia | CSS | 1159 | 356 | 30.7% | HUA: 57.63 ± 12.31; Non-HUA: 59.74 ± 10.25 | 60% | M ≥ 420, F ≥ 360 μmol/L | TyG, BMI |
| Tang 2025 b^58^ | China / Asia | CSS | 1238 | 368 | / | 59.05 ± 11.08 | 61% | M ≥ 420, F ≥ 360 μmol/L | eGDR |
| Uwakwe 2014^59^ | Nigeria / Africa | CSS | 100 | 45 | 45.0% | M: 55.1 ± 7.9; F: 55.7 ± 10 | 64% | > 416 μmol/L |  |
| Valbusa 2013^60^ | Italy / Europe | PCS | 400 | 73 | 18.3% | 64 ± 10 | 59% | M ≥ 416, F ≥ 357 μmol/L, or ULT | / |
| Vázquez-Rivera 2025^61^ | Mexico / North America | CSS | 1036 | 323 | 31.2% | DKD: 66 ± 16; Non-DKD: 61 ± 16 | 41% | M ≥ 7.0, F ≥ 6.0 mg/dL | DKD, Hypertension, High TG, High CHO, Hyperglicemia, rs11722228, rs1014290, rs3775948, rs7678287* |
| Wang 2013^62^ | China / Asia | CSS | 2917 | 951 | 32.6% | 59.6 ± 13.2 | 45% | M > 420, F > 360 μmol/L | Age, Gender, Duration of DM, Current smoking, Current drinking, BMI, WC, Hypertension, HbA1c, FPG, eGFR, TC, TG,HDL-C, LDL-C |
| Wang 2024 a^63^ | China / Asia | CSS | 903 | 316 | 35.0% | 54.7 ± 8.2 | 53% | > 420 μmol/L | Perirenal fat thickness (PrFT), MHR |
| Wang 2025^64^ | China / Asia | CSS | 1588 | 158 | 9.9% | Medium: 62 | 53% | > 420 μmol/L | Sex, Smoking, Drinking, Hypertension, Fatty Liver, Age, Duration of DM, BMI, HbA1c, C-peptide, UACR, eGFR, TG, HDL-C, CVAI |
| Wei 2016^65^ | China / Asia | CSS | 2250 | 388 | 17.2% | 59.00 ± 15.00 | 54% | M ≥ 420, F ≥ 360 μmol/L | HbA1c |
| Woldeamlak 2019^66^ | Ethiopia / Africa | CSS | 384 | 121 | 31.5% | 55.74 ± 9.05 | 60% | ＞ 6.8 mg/dL | Duration of DM, Hypertension, SBP, DBP, Family history of DM, WC, BMI |
| Woyesa 2017^67^ | Ethiopia / Africa | CSS | 319 | 106 | 33.2% | 49.8 ± 9.8 | 66% | M > 7.2, F > 6.0 mg/dL | Age, Residence, Regular Exercise, Mets |
| Xia 2020^68^ | China / Asia | CSS | 2961 | 741 | 25.0% | 61 ± 11 | 51% | M ≥ 420, F ≥ 360 μmol/L | / |
| Xia 2026^69^ | China / Asia | CSS | 4520 | 550 | 12.2% | 54.8 ± 11.4 | 58% | ≥ 420 μmol/L | HbA1c, TyGI, Obesity, Sex, Smoking history, Drinking history, Hypertension |
| Yan 2015^70^ | China / Asia | CSS | 3017 | 565 | 18.7% | 60.5 ± 12.0 | 52% | M > 7.0, F > 6.0 mg/dL | / |
| Yan 2019^71^ | China / Asia | CSS | 2736 | 476 | 17.4% | 70.2 ± 7.7 | 52% | M ≥ 420, F ≥ 360 μmol/L | / |
| Yang 2024^72^ | China / Asia | CSS | 9488 | 2626 | 27.7% | 53 (42-61) | 60% | M ≥ 420, F ≥ 360 μmol/L | FPG, TG, TC, HDL-C, LDL-C, Apo A, Apo B, Lp(a), TyG, TG/HDL-C, LDL-C/HDL-C, TC/HDL, Non-HDL-C, Lipidemic-modulating drugs, Insulin, Cardiovascular drugs, Electrolytes acid–base balancing drugs, Adjuvant drugs for liver disease, Antipodagrics, Antiplatelet drug, Antithyroid drugs |
| Yang 2025^73^ | China / Asia | RCS | 897 | 151 | 16.8% | 75.73 ± 5.41 | 60% | > 420 μmol/L | / |
| Yi 2017^74^ | China / Asia | CSS | 3861 | 590 | 15.3% | 58.82 ± 12.97 | 48% | M > 420, F > 360 μmol/L | / |
| Zhang 2023^75^ | USA / North America | CSS | 5676 | 1492 | 26.3% | 59.36 ± 0.30 | 51% | M ≥ 420, F ≥ 360 μmol/L | / |
| Zhao 2025^76^ | China / Asia | CSS | 1036 | 121 | 11.7% | 48.6 ± 12.0 | 68% | ≥ 420 μmol/L | / |
| Zoppini 2009^77^ | Italy / Europe | PCS | 2726 | 438 | 16.1% | 67 ± 10 | 55% | M > 416, F > 386 μmol/L | / |
| Zou 2017^78^ | China / Asia | CSS | 338 | 62 | 18.3% | >51.5 years (74.6%) | 64% | M > 420, F > 360 μmol/L | / |

**Abbreviations:** CSS, cross-sectional study; RCS, retrospective cohort study; PCS, prospective cohort study; HUA, hyperuricemia; T2DM, type 2 diabetes mellitus; BMI, body mass index; WC, waist circumference; eGFR, estimated glomerular filtration rate; TyG, triglyceride-glucose index; HbA1c, glycated hemoglobin; TC, total cholesterol; TG, triglycerides; HDL-C, high-density lipoprotein cholesterol; LDL-C, low-density lipoprotein cholesterol; SBP, systolic blood pressure; DBP, diastolic blood pressure; Mets, metabolic syndrome; ULT, urate-lowering therapy; SD, standard deviation. Additional abbreviations are defined in the table footnotes.

Risk factors marked with an asterisk () were reported in the original studies but were excluded from the quantitative meta-analysis due to incompatible statistical methods (e.g., linear regression) or specific sub-population analyses.*

**Table S2:** Characteristics of the included studies of Gout

| Author (Year) | Country / Region | Study Design | Sample Size (N) | Gout (N) | Gout Prevalence | Age (Mean/SD) | Male(%) | Gout Definition | Reported Risk Factors |
| --- | --- | --- | --- | --- | --- | --- | --- | --- | --- |
| Cai 2024^79^ | China / Asia | CSS | 10535 | 600 | / | 55 (47-63) | 60% | 2015 ACR/EULAR | BMI, WC, WHR, VFA |
| Cai 2025^80^ | China / Asia | CSS | 14099 | 799 | 5.7% | 55 (47-64) | 61% | 1977 ACR criteria | CVAI |
| Collier 2016^81^ | England / Europe | CSS | 16988 | 1719 | 10.1% | 69.93 ± 10.81 | 55% | Recorded diagnosis using Read codes (v2), or ULT records | Male, age, BMI, TG, HDL, current smoker, eGFR stage, no of medications |
| Lee 2024^82^ | Korea / Asia | RCS | 757378 | 22122 | 2.9% | G1: 55.4 ± 11.8,  G2: 56.8 ± 10.7,  G3: 64.1 ± 12.0,  G4: 64.4 ± 10.4 | 63% | two outpatient visits or one hospitalisation under the ICD-10 code M10 within 5 years prior to the baseline health checkup. | / |
| Nata 2020^83^ | Thailand / Asia | CSS | 30377 | 1204 | 4.0% | 61.2 ± 10.9 | 31% | Medical records | / |
| Pan 2016^84^ | Singapore / Asia | PCS | 4005 | 156 | 3.9% | 62.1 ± 7.2 | / | Self-report of physician-diagnosed Gout | / |
| Suppiah 2008^85^ | New Zealand / Oceania | CSS | 265 | 59 | 22.3% | Gout:61.1 ± 10.2; Non-Gout: 55.9 ± 12.3 | 53% | Self report and Medical records | Male sex, Ethnicity, age, eGFR, Aspirin, Diuretics, High TG, low HDL |
| Wang 2024 b^86^ | China / Asia | CSS | 403 | 23 | 5.7% | DKD: 56 (51-63); Non-DKD: 58 (51-64) | 53% | History of Gout | / |
| Wei 2020^87^ | China / Asia | CSS | 4304 | 126 | 2.9% | SRC: 62.87 ± 11.17; Non-SRC: 56.88 ± 12.04 | 59% | Medical records | / |

**Abbreviations:** CSS, cross-sectional study; RCS, retrospective cohort study; PCS, prospective cohort study; ACR, American College of Rheumatology; EULAR, European Alliance of Associations for Rheumatology; ULT, urate-lowering therapy; ICD-10, International Classification of Diseases, Tenth Revision; BMI, body mass index; WC, waist circumference; WHR, waist-to-hip ratio; VFA, visceral fat area; CVAI, Chinese visceral adiposity index; TG, triglycerides; HDL, high-density lipoprotein cholesterol; LDL, low-density lipoprotein cholesterol; eGFR, estimated glomerular filtration rate; DKD, diabetic kidney disease; SD, standard deviation.

**References included in this study**

1. Abdel KA, Kalluvya SE, Sadiq AM, Ashir A, Masikini PI. Prevalence of hyperuricemia and associated factors among patients with type 2 diabetes mellitus in northwestern tanzania: A cross-sectional study. *Clin Med Insights Endocrinol Diabetes*. 2024;17:11795514241274694. doi:10.1177/11795514241274694

2. Abudureyimu P, Pang Y, Huang L, et al. A predictive model for hyperuricemia among type 2 diabetes mellitus patients in urumqi, China. *BMC Public Health*. 2023;23(1):1740. doi:10.1186/s12889-023-16669-6

3. Abujbara M, Al Hourani HM, Al-Raoush RI, Khader YS, Ajlouni K. Prevalence of hyperuricemia and associated factors among type 2 diabetic patients in jordan. *Int J Gen Med*. 2022;15:6611-6619. doi:10.2147/IJGM.S376857

4. Araújo Intchasso Adotey SA, Zhang Q, Chen M, et al. Correlation between Chinese visceral adiposity index and serum uric acid levels in type 2 diabetes mellitus patients. *Front Endocrinol*. 2025;16. doi:10.3389/fendo.2025.1479662

5. Aa A, Ak J, Oa A, Go O. Serum uric acid level as an independent component of the metabolic syndrome in type 2 diabetic blacks. *Nigerian journal of clinical practice*. 2007;10(2). Accessed March 7, 2026. https://pubmed.ncbi.nlm.nih.gov/17902506/

6. Andrade J a. M, Kang HC, Greffin S, Garcia Rosa ML, Lugon JR. Serum uric acid and disorders of glucose metabolism: The role of glycosuria. *Braz J Med Biol Res*. 2014;47(10):917-923. doi:10.1590/1414-431X20143878

7. Arersa KK, Wondimnew T, Welde M, Husen TM. Prevalence and determinants of hyperuricemia in type 2 diabetes mellitus patients attending jimma medical center, southwestern ethiopia, 2019. *Diabetes Metab Syndr Obes*. 2020;13:2059-2067. doi:10.2147/DMSO.S252825

8. Barry NOK, Djite M, Kandji PM, et al. Association of hyperuricemia and metabolic syndrome in type 2 diabetes mellitus patients in dakar. *AJBR*. 2021;15(3):43-48. doi:10.5897/AJBR2020.1111

9. Bo S, Cavallo-Perin P, Gentile L, Repetti E, Pagano G. Hypouricemia and hyperuricemia in type 2 diabetes: Two different phenotypes. *Eur J Clin Invest*. 2001;31(4):318-321. doi:10.1046/j.1365-2362.2001.00812.x

10. Chen Q, Hu H, She Y, et al. An artificial neural network model for evaluating the risk of hyperuricaemia in type 2 diabetes mellitus. *Sci Rep*. 2024;14(1):2197. doi:10.1038/s41598-024-52550-1

11. Chen X yu, Lu F, Zhang J, et al. The effect of hyperuricemia and its interaction with hypertension towards chronic kidney disease in patients with type 2 diabetes: Evidence from a cross- sectional study in eastern China. *Front Endocrinol*. 2024;15. doi:10.3389/fendo.2024.1415459

12. Choukem SP, Mengue JA, Doualla MS, Donfack OT, Beyiha G, Luma HN. Hyperuricaemia in patients with type 2 diabetes in a tertiary healthcare centre in sub-saharan africa: Prevalence and determinants. *Trop Doct*. 2016;46(4):216-221. doi:10.1177/0049475515626030

13. Deng Z, Gu Y, Hou X, et al. Association between uric acid, cancer incidence and mortality in patients with type 2 diabetes: Shanghai diabetes registry study. doi:10.1002/dmrr.2724

14. Donkeng M, Kuaté D, Koudjou PN, Noubiap JJ, Kuiate JR. Association between hyperuricemia and glycated hemoglobin in type 2 diabetes at the district hospital of dschang. *Pan Afr Med J*. 2021;40:177. doi:10.11604/pamj.2021.40.177.30207

15. Eljaaly Z, Mujammami M, Nawaz SS, Rafiullah M, Siddiqui K. <p>Risk predictors of high uric acid levels among patients with type-2 diabetes</p>. *DMSO*. 2021;14:4911-4920. doi:10.2147/DMSO.S344894

16. Fayazi HS, Mortazavi Khatibani SS, Motamed B, Yaseri M. Evaluation of levels of uric acid and lipid profile in hospitalized patients with diabetes. *BMC Research Notes*. 2023;16(1):154. doi:10.1186/s13104-023-06429-5

17. H F, Ne H, S EA, S B, A C. Risk factors associated with hyperuricemia in patients with diabetes type 2: About 190 cases. *Diab Res Open Access*. 2020;2(1):12-16. doi:10.36502/2020/droa.6163

18. Fouad M, Fathy H, Zidan A. Serum uric acid and its association with hypertension, early nephropathy and chronic kidney disease in type 2 diabetic patients. *Braz J Nephrol*. 2016;38:403-410. doi:https://doi.org/10.5935/0101-2800.20160065

19. Gao Z, Zuo M, Han F, et al. Renal impairment markers in type 2 diabetes patients with different types of hyperuricemia. *J Diabetes Investig*. 2019;10(1):118-123. doi:10.1111/jdi.12850

20. Gobusamang E, Nyepetsi NG, Motswaledi MS, Kasvosve I. Hyperuricaemia is associated with dyslipidemia but not HbA1c among type 2 diabetes mellitus patients in botswana. *African Journal of Laboratory Medicine*. 2019;8(1):4. doi:10.4102/ajlm.v8i1.786

21. Guo M, Niu JY, Li SR, et al. Gender differences in the association between hyperuricemia and diabetic kidney disease in community elderly patients. *J Diabetes Complications*. 2015;29(8):1042-1049. doi:10.1016/j.jdiacomp.2015.08.016

22. Han Y, Zhang M, Lu J, et al. Hyperuricemia and overexcretion of uric acid increase the risk of simple renal cysts in type 2 diabetes. *Sci Rep*. 2017;7(1):3802. doi:10.1038/s41598-017-04036-6

23. Hermans MP, Rousseau MF. Raised natriuretic peptides, big-endothelin-1 and improved beta-cell function in type 2 diabetic males with hyperuricaemia. *Diabetes & Vascular Disease Research*. 2009;6(3):190-193. doi:10.1177/1479164109336689

24. Hu Y, Chan Z, Li C, et al. Higher serum uric acid levels are associated with an increased risk of vision-threatening diabetic retinopathy in type 2 diabetes patients. *Invest Ophthalmol Vis Sci*. 2021;62(4):23. doi:10.1167/iovs.62.4.23

25. Huang B, Li X, Zhang XX, et al. MHR was associated with hyperuricemia risk in patients with type 2 diabetes mellitus: The mediating effect of body mass index. *DMSO*. 2025;18:3015-3025. doi:10.2147/DMSO.S535669

26. Kim TH, Lee SS, Yoo JH, et al. The relationship between the regional abdominal adipose tissue distribution and the serum uric acid levels in people with type 2 diabetes mellitus. *Diabetol Metab Syndr*. 2012;4(1):3. doi:10.1186/1758-5996-4-3

27. Kim K, Han K, Kim IY, et al. Association between diabetes duration and hyperuricemia: Korea national health and nutrition examination survey 2016 to 2021. *Korean J Intern Med*. 2026;41(1):143-151. doi:10.3904/kjim.2024.436

28. Li Y, Fan X, Li C, et al. The relationships among hyperuricemia, body mass index and impaired renal function in type 2 diabetic patients. *Endocr J*. 2018;65(3):281-290. doi:10.1507/endocrj.EJ17-0266

29. Liang CC, Lin PC, Lee MY, et al. Association of serum uric acid concentration with diabetic retinopathy and albuminuria in taiwanese patients with type 2 diabetes mellitus. *International Journal of Molecular Sciences*. 2016;17(8):1248. doi:10.3390/ijms17081248

30. Lin H, Xu J, Teng C. Correlation between remnant cholesterol and hyperuricemia in patients with type 2 diabetes mellitus: A cross-sectional study. *Lipids Health Dis*. 2024;23(1):155. doi:10.1186/s12944-024-02148-3

31. Liu Y, Zhao X, Yang Z, Wang S, Han C, Zhang H. Correlation between serum C-peptide-releasing effects and the risk of elevated uric acid in type 2 diabetes mellitus. *Endocr J*. 2022;69(7):773-784. doi:10.1507/endocrj.EJ21-0492

32. Liu P, Ji B, Peng Y. Correlation between loge GDR and hyperuricemia in patients with type 2 diabetes mellitus: A cross-sectional study. *Front Endocrinol*. 2025;16. doi:10.3389/fendo.2025.1637373

33. Lopes AC, Lourenço O, Roque F, Morgado M. Clinical and pharmacotherapeutic profile of patients with type 2 diabetes mellitus admitted to a hospital emergency department. *Biomedicines*. 2023;11(2):256. doi:10.3390/biomedicines11020256

34. Lu W, Zhao X, Sheng J, et al. Hip circumference has independent association with the risk of hyperuricemia in middle-aged but not in older male patients with type 2 diabetes mellitus. *Nutr J*. 2023;22(1):45. doi:10.1186/s12937-023-00874-5

35. Luo Y, Hao J, He X, et al. Association between triglyceride-glucose index and serum uric acid levels: A biochemical study on anthropometry in non-obese type 2 diabetes mellitus patients. *Diabetes Metab Syndr Obes*. 2022;15:3447-3458. doi:10.2147/DMSO.S387961

36. Mantovani A, Rigolon R, Pichiri I, et al. Hyperuricemia is associated with an increased prevalence of atrial fibrillation in hospitalized patients with type 2 diabetes. *J Endocrinol Invest*. 2016;39(2):159-167. doi:10.1007/s40618-015-0354-z

37. Mantovani A, Rigolon R, Civettini A, et al. Hyperuricemia is associated with an increased prevalence of paroxysmal atrial fibrillation in patients with type 2 diabetes referred for clinically indicated 24-h holter monitoring. *J Endocrinol Invest*. 2018;41(2):223-231. doi:10.1007/s40618-017-0729-4

38. Mirghani HO. Hypertriglyceridemia, hyperuricemia, and anemia among sudanese patients with type 2 diabetes mellitus. Indian J Basic Appl Med Res. 2018;7(3):493–501.

39. Mundhe S, Mhasde D. The study of prevalence of hyperuricemia and metabolic syndrome in type 2 diabetes mellitus. *Int J Adv Med*. Published online 2016:241-249. doi:10.18203/2349-3933.ijam20160655

40. Ndang Ngou Milama S, Mougougou A, Olagui SG, et al. Analysis of the factors associated with ED in type 2 diabetics at the university hospital of libreville. *Sex Med*. 2022;10(6):100564. doi:10.1016/j.esxm.2022.100564

41. Ogbera AO, Azenabor AO. Hyperuricaemia and the metabolic syndrome in type 2 DM. *Diabetol Metab Syndr*. 2010;2:24. doi:10.1186/1758-5996-2-24

42. Paduraru L, Vesa CM, Popoviciu MS, Ghitea TC, Zaha DC. Interrelationship between dyslipidemia and hyperuricemia in patients with uncontrolled type 2 diabetes: Clinical implications and a risk identification algorithm. *Healthcare (Basel)*. 2025;13(20):2605. doi:10.3390/healthcare13202605

43. Panero F, Gruden G, Perotto M, et al. Uric acid is not an independent predictor of cardiovascular mortality in type 2 diabetes: A population-based study. *Atherosclerosis*. 2012;221(1):183-188. doi:10.1016/j.atherosclerosis.2011.11.042

44. Patel S, Singh M, Kahlon N. Association of serum uric acid levels with glycated haemoglobin in diabetic patients and healthy controls. *J Family Med Prim Care*. 2024;13(11):5040-5046. doi:10.4103/jfmpc.jfmpc_777_24

45. Peng YF, Yin H, Hu L, Fang L, Jia DR, Li L. White blood cell count is associated with hyperuricemia in patients with type 2 diabetes mellitus. *J Inflamm Res*. 2025;18:3993-3999. doi:10.2147/JIR.S501890

46. Peralta-Vera FG, Castillo-Céspedes E, Collazos-Huamán LDC, Guerreros-Espino C, Herrera-Añazco P, Benites-Zapata VA. Association between abdominal obesity and hyperuricemia in peruvian adults with type 2 diabetes mellitus. *Clin Med Insights Endocrinol Diabetes*. 2025;18:11795514251403098. doi:10.1177/11795514251403098

47. Qin Y, Zhang S, Cui S, et al. High urinary excretion rate of glucose attenuates serum uric acid level in type 2 diabetes with normal renal function. *J Endocrinol Invest*. 2021;44(9):1981-1988. doi:10.1007/s40618-021-01513-8

48. Qin Y, Huang C, Wei W, Zhang Y. Influence of serum uric acid on bone mineral density across body mass index categories in men with type 2 diabetes: A cross-sectional study. *BMC Musculoskelet Disord*. 2025;26(1):1043. doi:10.1186/s12891-025-09169-8

49. Rafiullah M, Siddiqui K, Al-Rubeaan K. Association between serum uric acid levels and metabolic markers in patients with type 2 diabetes from a community with high diabetes prevalence. doi:10.1111/ijcp.13466

50. Shang C, Yuan M, Wang Y, et al. Association between visceral obesity and glycemic control in patients with type 2 diabetes mellitus: A retrospective study. *Diabetes Metab Syndr Obes*. 2024;17:2869-2880. doi:10.2147/DMSO.S470836

51. Sheikhbahaei S, Fotouhi A, Hafezi-Nejad N, Nakhjavani M, Esteghamati A. Serum uric acid, the metabolic syndrome, and the risk of chronic kidney disease in patients with type 2 diabetes. *Metabolic Syndrome and Related Disorders*. 2014;12(2):102-109. doi:10.1089/met.2013.0119

52. Shi R, Niu Z, Wu B, Hu F. Study on the risk factors for hyperuricaemia and related vascular complications in patients with type 2 diabetes mellitus. *Risk Manag Healthc Policy*. 2020;13:1661-1675. doi:10.2147/RMHP.S255042

53. Shou Z, Zhao Y, Zhang Y, Li S. Risk factors for peripheral arterial disease in elderly patients with type-2 diabetes mellitus: A clinical study. *Pak J Med Sci*. 2020;36(6):1344-1348. doi:10.12669/pjms.36.6.2906

54. Singh SK, Singh R, Singh SK. Prevalence of hypouricemia and hyperuricemia and looking beyond serum uric acid in patients with newly onset type 2 diabetes mellitus in eastern part of uttar pradesh: A cross-sectional study. *J Assoc Physicians India*. 2023;71(5):11-12. doi:10.5005/japi-11001-0239

55. Sun X, Li X, Qian Z, et al. Association between the triglyceride-glucose index and hyperuricemia in patients with type 2 diabetes mellitus. *Front Endocrinol (Lausanne)*. 2025;16:1666563. doi:10.3389/fendo.2025.1666563

56. Tanaka K, Hara S, Kushiyama A, et al. Risk of macrovascular disease stratified by stage of chronic kidney disease in type 2 diabetic patients: Critical level of the estimated glomerular filtration rate and the significance of hyperuricemia. *Clin Exp Nephrol*. 2011;15(3):391-397. doi:10.1007/s10157-011-0420-6

57. Tang D, Gu X, Xuan Y, Zhu F, Shen Y, Lu L. Association between the triglyceride-glucose index and hyperuricemia: Potential role of obesity in patients with type 2 diabetes mellitus. *Front Endocrinol (Lausanne)*. 2025;16:1637543. doi:10.3389/fendo.2025.1637543

58. Tang D, Gao P, Xie W, Wang S, Wang X, Lu L. Associations between estimated glucose disposal rate and hyperuricemia in patients with type 2 diabetes mellitus: A cross-sectional study. *BMC Nephrol*. 2025;26(1):663. doi:10.1186/s12882-025-04601-6

59. Uwakwe J, Agboghoroma O, Akanbi F, Odoh G, Puepet F. Hyperuricaemia in obese type 2 diabetics in Jos, north central Nigeria. Jos J Med. 2014;8(3):25–9.

60. Valbusa F, Bertolini L, Bonapace S, et al. Relation of elevated serum uric acid levels to incidence of atrial fibrillation in patients with type 2 diabetes mellitus. *American Journal of Cardiology*. 2013;112(4):499-504. doi:10.1016/j.amjcard.2013.04.012

61. Vázquez-Rivera GE, Gómez-García EF, Parra-Michel R, et al. Single nucleotide variants of the *SLC2A9* gene are associated with hyperuricemia in mexican patients with type 2 diabetes. *Archives of Medical Research*. 2025;56(6):103235. doi:10.1016/j.arcmed.2025.103235

62. Wang J, Chen RP, Lei L, et al. Prevalence and determinants of hyperuricemia in type 2 diabetes mellitus patients with central obesity in guangdong province in China. *Asia Pac J Clin Nutr*. 2013;22(4):590-598. doi:10.6133/apjcn.2013.22.4.16

63. Wang W, Tu M, Qiu XP, Tong Y, Guo XL. The interplay of systemic inflammation and oxidative stress in connecting perirenal adipose tissue to hyperuricemia in type 2 diabetes mellitus: A mediation analysis. *J Inflamm Res*. 2024;17:11319-11329. doi:10.2147/JIR.S488964

64. Wang Q, Liu T, Jia C, Wang P, Wang Y, He Q. Association of Chinese visceral adiposity index with asymptomatic hyperuricemia incidence in type 2 diabetes: A cross-sectional study. *PeerJ*. 2025;13:e19045. doi:10.7717/peerj.19045

65. Wei F, Chang B, Yang X, Wang Y, Chen L, Li WD. Serum uric acid levels were dynamically coupled with hemoglobin A1c in the development of type 2 diabetes. *Sci Rep*. 2016;6(1):28549. doi:10.1038/srep28549

66. Woldeamlak B, Yirdaw K, Biadgo B. Hyperuricemia and its association with cardiovascular disease risk factors in type two diabetes mellitus patients at the university of gondar hospital, northwest ethiopia. *EJIFCC*. 2019;30(3):325-339.

67. Woyesa SB, Hirigo AT, Wube TB. Hyperuricemia and metabolic syndrome in type 2 diabetes mellitus patients at hawassa university comprehensive specialized hospital, south west ethiopia. *BMC Endocr Disord*. 2017;17(1):76. doi:10.1186/s12902-017-0226-y

68. Xia Q, Zhang SH, Yang SM, et al. Serum uric acid is independently associated with diabetic nephropathy but not diabetic retinopathy in patients with type 2 diabetes mellitus. *J Chin Med Assoc*. 2020;83(4):350-356. doi:10.1097/JCMA.0000000000000285

69. Xia D, Li C, Sun X, et al. Exploration and clinical analysis of the correlation between the triglyceride–glucose index and hyperuricemia in patients with type 2 diabetes: A real-world, retrospective, cross-sectional study with subsequent cohort analysis. *BMC Endocr Disord*. 2026;26(1):65. doi:10.1186/s12902-026-02188-z

70. Yan D, Tu Y, Jiang F, et al. Uric acid is independently associated with diabetic kidney disease: A cross-sectional study in a Chinese population. *PLoS One*. 2015;10(6):e0129797. doi:10.1371/journal.pone.0129797

71. Yan ST, Jia JH, Lv XF, et al. Glycemic control and comprehensive metabolic risk factors control in older adults with type 2 diabetes. *Exp Gerontol*. 2019;127:110713. doi:10.1016/j.exger.2019.110713

72. Yang J, Zhou K fan, Tao G dong, Wei B, Lu Y wei. The predictive value of TyG and lipid ratios on the development of complications and hyperuricemia in patients with type 2 diabetes mellitus. *Lipids*. 2024;59(6):209-219. doi:10.1002/lipd.12411

73. Yang Y, Zhang Y, Tian Z. Elevated waist-to-height ratio increases the risk of cardiovascular and cerebrovascular disease mortality in elderly type 2 diabetes mellitus populations. *J Multidiscip Healthc*. 2025;18:2681-2692. doi:10.2147/JMDH.S521758

74. Yi M, Chen R ‐P., Yang R, Chen H. Increased prevalence and risk of non‐alcoholic fatty liver disease in overweight and obese patients with type 2 diabetes in south China. *Diabet Med*. 2017;34(4):505-513. doi:10.1111/dme.13174

75. Zhang J, Chen Y, Zou L, et al. Dose–response relationship between dietary antioxidant intake and diabetic kidney disease in the US adults with diabetes. *Acta Diabetol*. 2023;60(10):1365-1375. doi:10.1007/s00592-023-02125-9

76. Zhao M, Ma X, Xia Z, et al. Association between serum uric acid and dyslipidaemia in type 2 diabetes: A cross-sectional study. *BMJ Open*. 2025;15(1):e087954. doi:10.1136/bmjopen-2024-087954

77. Zoppini G, Targher G, Negri C, et al. Elevated serum uric acid concentrations independently predict cardiovascular mortality in type 2 diabetic patients. *Diabetes Care*. 2009;32(9):1716-1720. doi:10.2337/dc09-0625

78. Zou D, Ye Y, Zou N, Yu J. Analysis of risk factors and their interactions in type 2 diabetes mellitus: A cross-sectional survey in guilin, China. *Journal of Diabetes Investigation*. 2017;8(2):188-194. doi:10.1111/jdi.12549

79. Cai N, Chen M, Feng P, et al. Relationships between obesity and prevalence of Gout in patients with type 2 diabetes mellitus: A cross-sectional population-based study. *BMC Endocrine Disorders*. 2024;24(1):137. doi:10.1186/s12902-024-01672-8

80. Cai N, Chen M, Feng P, Wang Y, Zhu X, Zheng Q. Relationship between the Chinese visceral adiposity index and Gout in individuals with type 2 diabetes mellitus: A cross-sectional population-based study. *Front Nutr*. 2025;12:1697822. doi:10.3389/fnut.2025.1697822

81. Collier A, Stirling A, Cameron L, Hair M, Crosbie D. Gout and diabetes: A common combination. *Postgrad Med J*. 2016;92(1089):372-378. doi:10.1136/postgradmedj-2015-133691

82. Lee DY, Moon JS, Jung I, et al. Risk acceleration by Gout on major adverse cardiovascular events and all‐cause death in patients with diabetes and chronic kidney disease. Published online 2024. doi:10.1111/dom.16165

83. Nata N, Rangsin R, Supasyndh O, Satirapoj B. Impaired glomerular filtration rate in type 2 diabetes mellitus subjects: A nationwide cross-sectional study in thailand. *J Diabetes Res*. 2020;2020:6353949. doi:10.1155/2020/6353949

84. Pan A, Teng GG, Yuan JM, Koh WP. Bidirectional association between diabetes and Gout: The singapore Chinese health study. *Sci Rep*. 2016;6:25766. doi:10.1038/srep25766

85. Suppiah R, Dissanayake A, Dalbeth N. High prevalence of Gout in patients with type 2 diabetes: Male sex, renal impairment, and diuretic use are major risk factors. *N Z Med J*. 2008;121(1283):43-50.

86. Wang X, Liu X, Zhao J, Chen M, Wang L. Construction of a nomogram-based prediction model for the risk of diabetic kidney disease in T2DM. *DMSO*. 2024;17:215-225. doi:10.2147/DMSO.S442925

87. Wei L, Xiao Y, Xiong X, et al. The relationship between simple renal cysts and renal function in patients with type 2 diabetes. *Front Physiol*. 2020;11. doi:10.3389/fphys.2020.616167

**Table S3.** The quality assessment of cross-sectional studies.

| Author＆Year | 1 | 2 | 3 | 4 | 5 | 6 | 7 | 8 | 9 | 10 | 11 | total |
| --- | --- | --- | --- | --- | --- | --- | --- | --- | --- | --- | --- | --- |
| Abdel 2024 | 1 | 1 | 1 | 1 | 1 | 0 | 1 | 1 | 0 | 0 | 0 | 7 |
| Abudureyimu 2023 | 1 | 1 | 1 | 1 | 0 | 0 | 1 | 1 | 0 | 0 | 0 | 6 |
| Abujbara 2022 | 1 | 1 | 1 | 0 | 0 | 0 | 1 | 1 | 0 | 0 | 0 | 5 |
| Adotey 2025 | 1 | 1 | 1 | 1 | 0 | 0 | 1 | 1 | 0 | 0 | 0 | 6 |
| Akande 2006 | 1 | 1 | 0 | 0 | 0 | 0 | 1 | 1 | 0 | 0 | 0 | 4 |
| Andrade 2014 | 1 | 1 | 1 | 1 | 0 | 1 | 1 | 1 | 0 | 0 | 0 | 7 |
| Arersa 2020 | 1 | 1 | 1 | 1 | 0 | 1 | 1 | 1 | 0 | 0 | 0 | 7 |
| Barry 2021 | 1 | 1 | 1 | 0 | 0 | 0 | 1 | 1 | 0 | 0 | 0 | 5 |
| Bo 2001 | 1 | 1 | 1 | 1 | 0 | 1 | 1 | 1 | 0 | 1 | 0 | 8 |
| Cai 2024 | 1 | 1 | 1 | 1 | 0 | 1 | 1 | 1 | 1 | 0 | 0 | 8 |
| Cai 2025 | 1 | 1 | 1 | 1 | 0 | 1 | 1 | 1 | 1 | 0 | 0 | 8 |
| Chen 2024a | 1 | 1 | 1 | 1 | 0 | 1 | 1 | 1 | 1 | 0 | 0 | 8 |
| Chen 2024b | 1 | 1 | 1 | 1 | 1 | 0 | 1 | 1 | 0 | 1 | 0 | 8 |
| Choukem 2016 | 1 | 1 | 1 | 1 | 0 | 1 | 1 | 1 | 0 | 0 | 0 | 7 |
| Collier 2016 | 1 | 0 | 1 | 1 | 0 | 0 | 0 | 1 | 1 | 0 | 0 | 5 |
| Donkeng 2021 | 1 | 1 | 1 | 0 | 0 | 0 | 1 | 0 | 0 | 1 | 0 | 5 |
| Eljaaly 2021 | 1 | 1 | 1 | 0 | 0 | 1 | 1 | 1 | 0 | 0 | 0 | 6 |
| Fayazi 2023 | 1 | 1 | 1 | 1 | 0 | 0 | 1 | 0 | 0 | 0 | 0 | 5 |
| Fennoun 2020 | 1 | 1 | 1 | 0 | 0 | 0 | 1 | 1 | 0 | 0 | 0 | 5 |
| Fouad 2016 | 1 | 1 | 1 | 0 | 0 | 0 | 1 | 1 | 0 | 0 | 0 | 5 |
| Gao 2019 | 1 | 1 | 1 | 0 | 0 | 0 | 1 | 1 | 0 | 0 | 0 | 5 |
| Gobusamang 2019 | 1 | 1 | 1 | 0 | 0 | 1 | 1 | 0 | 1 | 0 | 0 | 6 |
| Guo 2015 | 1 | 1 | 1 | 1 | 0 | 1 | 1 | 1 | 1 | 1 | 0 | 9 |
| Han 2017 | 1 | 1 | 1 | 1 | 0 | 1 | 1 | 1 | 1 | 0 | 0 | 8 |
| Hermans 2009 | 1 | 1 | 0 | 0 | 0 | 1 | 1 | 1 | 0 | 0 | 0 | 5 |
| Hu 2021 | 1 | 1 | 1 | 1 | 0 | 1 | 1 | 1 | 0 | 0 | 0 | 7 |
| Huang 2025 | 1 | 1 | 1 | 0 | 0 | 0 | 1 | 1 | 1 | 0 | 0 | 6 |
| Kim 2012 | 1 | 1 | 1 | 0 | 1 | 1 | 1 | 1 | 1 | 0 | 0 | 8 |
| Kim 2026 | 1 | 1 | 1 | 1 | 0 | 0 | 1 | 1 | 1 | 0 | 0 | 7 |
| Li 2018 | 1 | 1 | 0 | 1 | 0 | 0 | 1 | 1 | 1 | 1 | 0 | 7 |
| Liang 2016 | 1 | 1 | 1 | 0 | 0 | 0 | 1 | 1 | 0 | 0 | 0 | 5 |
| Lin 2024 | 1 | 1 | 0 | 1 | 0 | 0 | 1 | 1 | 1 | 1 | 0 | 7 |
| Liu 2022 | 1 | 1 | 1 | 1 | 0 | 1 | 1 | 1 | 0 | 0 | 0 | 7 |
| Liu 2025 | 1 | 1 | 1 | 1 | 0 | 1 | 1 | 1 | 0 | 0 | 0 | 7 |
| Lopes 2023 | 1 | 1 | 1 | 1 | 0 | 0 | 1 | 1 | 1 | 0 | 0 | 7 |
| Lu 2023 | 1 | 1 | 1 | 1 | 0 | 1 | 1 | 1 | 0 | 0 | 0 | 7 |
| Luo 2022 | 1 | 1 | 1 | 0 | 0 | 0 | 1 | 1 | 1 | 0 | 0 | 6 |
| Mantovani 2016 | 1 | 1 | 1 | 1 | 0 | 1 | 1 | 1 | 1 | 0 | 0 | 8 |
| Mantovani 2018 | 1 | 1 | 1 | 1 | 0 | 0 | 1 | 1 | 1 | 0 | 0 | 7 |
| Mirghani 2018 | 1 | 1 | 1 | 0 | 0 | 0 | 0 | 0 | 0 | 0 | 0 | 3 |
| Mundhe 2016 | 1 | 1 | 0 | 1 | 0 | 1 | 1 | 0 | 0 | 0 | 0 | 5 |
| Nata 2020 | 1 | 1 | 1 | 1 | 0 | 1 | 1 | 1 | 0 | 0 | 0 | 7 |
| Ndang Ngou Milama 2022 | 1 | 1 | 1 | 0 | 0 | 0 | 1 | 1 | 1 | 0 | 0 | 6 |
| Ogbera 2010 | 1 | 1 | 1 | 0 | 0 | 1 | 1 | 1 | 0 | 0 | 0 | 6 |
| Paduraru 2025 | 1 | 1 | 1 | 1 | 0 | 0 | 1 | 1 | 1 | 0 | 0 | 7 |
| Patel 2024 | 1 | 1 | 0 | 0 | 0 | 0 | 1 | 1 | 1 | 0 | 0 | 5 |
| Peng 2025 | 1 | 1 | 1 | 1 | 0 | 0 | 1 | 1 | 1 | 0 | 0 | 7 |
| Peralta-Vera 2025 | 1 | 1 | 1 | 0 | 0 | 1 | 1 | 1 | 1 | 0 | 0 | 7 |
| Qin 2021 | 1 | 1 | 1 | 0 | 0 | 1 | 1 | 1 | 1 | 0 | 0 | 7 |
| Qin 2025 | 1 | 1 | 1 | 1 | 0 | 0 | 1 | 1 | 1 | 0 | 0 | 7 |
| Rafiullah 2020 | 1 | 0 | 1 | 1 | 0 | 0 | 1 | 1 | 0 | 0 | 0 | 5 |
| Shang 2024 | 1 | 1 | 1 | 1 | 0 | 0 | 1 | 1 | 1 | 0 | 0 | 7 |
| Sheikhbahaei 2014 | 1 | 1 | 1 | 1 | 0 | 1 | 1 | 1 | 0 | 0 | 0 | 7 |
| Shi 2020 | 1 | 1 | 1 | 1 | 0 | 1 | 1 | 1 | 1 | 1 | 1 | 10 |
| Shou 2020 | 1 | 1 | 1 | 0 | 0 | 0 | 1 | 1 | 0 | 0 | 0 | 5 |
| Singh 2023 | 1 | 1 | 1 | 0 | 0 | 0 | 1 | 1 | 0 | 0 | 0 | 5 |
| Sun 2025 | 1 | 1 | 1 | 1 | 0 | 0 | 1 | 1 | 0 | 0 | 0 | 6 |
| Suppiah 2008 | 1 | 0 | 1 | 1 | 0 | 0 | 0 | 1 | 0 | 0 | 0 | 4 |
| Tanaka 2011 | 1 | 1 | 1 | 0 | 0 | 0 | 0 | 1 | 0 | 0 | 0 | 4 |
| Tang 2025 a | 1 | 1 | 1 | 1 | 0 | 0 | 1 | 1 | 1 | 0 | 0 | 7 |
| Tang 2025 b | 1 | 1 | 1 | 1 | 0 | 0 | 1 | 1 | 1 | 0 | 0 | 7 |
| Uwakwe 2014 | 1 | 1 | 0 | 0 | 0 | 0 | 0 | 1 | 0 | 0 | 0 | 3 |
| Vázquez-Rivera 2025 | 1 | 1 | 0 | 0 | 0 | 1 | 1 | 1 | 0 | 0 | 0 | 5 |
| Wang 2013 | 1 | 1 | 1 | 0 | 0 | 1 | 1 | 1 | 0 | 0 | 0 | 6 |
| Wang 2024 a | 1 | 1 | 1 | 0 | 0 | 1 | 1 | 1 | 1 | 0 | 0 | 7 |
| Wang 2024 b | 1 | 1 | 1 | 0 | 0 | 0 | 0 | 1 | 0 | 0 | 0 | 4 |
| Wang 2025 | 1 | 1 | 1 | 0 | 0 | 0 | 1 | 1 | 1 | 0 | 0 | 6 |
| Wei 2016 | 1 | 1 | 1 | 0 | 0 | 0 | 0 | 1 | 0 | 0 | 0 | 4 |
| Wei 2020 | 1 | 0 | 0 | 0 | 0 | 0 | 1 | 1 | 1 | 0 | 0 | 4 |
| Woldeamlak 2019 | 1 | 1 | 1 | 1 | 0 | 0 | 1 | 1 | 0 | 1 | 0 | 7 |
| Woyesa 2017 | 1 | 1 | 1 | 1 | 0 | 1 | 1 | 1 | 1 | 1 | 0 | 9 |
| Xia 2020 | 1 | 1 | 1 | 1 | 0 | 0 | 1 | 1 | 1 | 0 | 0 | 7 |
| Xia 2026 | 1 | 1 | 1 | 1 | 0 | 0 | 1 | 1 | 0 | 0 | 1 | 7 |
| Yan 2015 | 1 | 1 | 0 | 0 | 0 | 0 | 1 | 1 | 0 | 0 | 0 | 4 |
| Yan 2019 | 1 | 1 | 1 | 0 | 1 | 1 | 1 | 1 | 1 | 0 | 0 | 8 |
| Yang 2024 | 1 | 1 | 1 | 0 | 0 | 0 | 1 | 1 | 0 | 0 | 0 | 5 |
| Yi 2017 | 1 | 1 | 1 | 0 | 1 | 0 | 1 | 1 | 0 | 0 | 0 | 6 |
| Zhang 2023 | 1 | 1 | 1 | 1 | 0 | 0 | 1 | 1 | 1 | 0 | 1 | 8 |
| Zhao 2025 | 1 | 1 | 1 | 1 | 1 | 0 | 1 | 1 | 1 | 0 | 0 | 8 |
| Zou 2017 | 1 | 1 | 1 | 1 | 0 | 1 | 1 | 1 | 1 | 0 | 0 | 8 |

AHRQ was used to evaluate the quality of cross-sectional studies.

**Table S4.** The quality assessment of cohort studies.

| Author＆Year | Selection | | | | Comparability | Outcome | | | Total score |
| --- | --- | --- | --- | --- | --- | --- | --- | --- | --- |
| Deng 2016 | 1 | 1 | 1 | 1 | 2 | 1 | 1 | 0 | 8 |
| Lee 2024 | 1 | 1 | 1 | 1 | 2 | 1 | 1 | 1 | 9 |
| Pan 2016 | 1 | 1 | 1 | 1 | 2 | 1 | 1 | 1 | 9 |
| Panero 2012 | 1 | 1 | 1 | 1 | 2 | 1 | 1 | 1 | 9 |
| Valbusa 2013 | 1 | 1 | 1 | 1 | 2 | 1 | 1 | 1 | 9 |
| Yang 2025 | 1 | 0 | 1 | 0 | 1 | 1 | 1 | 1 | 6 |
| Zoppini 2009 | 1 | 1 | 1 | 1 | 2 | 1 | 1 | 0 | 8 |

The NOS scale was used to evaluate the quality of the cohort studies.

**Table S5** Sex(Male) Subgroup Analysis Results of HUA prevalence

| **Subgroup** | **No. of cohorts** | **HUA Prevalence (95%CI) (%)** | **P for heterogeneity** | **I2%** |
| --- | --- | --- | --- | --- |
| **Male** | 48 | 22.2 (20.4 , 23.9) | <0.001 | 95.4 |
| **Diagnostic Criteria** |  |  |  |  |
| > 7.0 mg/dL | 42 | 21.2 (19.3 , 23.0) | <0.001 | 95.6 |
| > 6.8 mg/dL | 2 | 33.3 (28.2 , 38.4) | 0.932 | 0 |
| > 7.2 mg/dL | 2 | 26.8 (14.5 , 39.1) | 0.004 | 87.8 |
| ≥6.0 mg/dL | 1 | 29.0 (25.1 , 33.0) | / | / |
| ＞450 μmol/L | 1 | 33.8 (26.1 , 41.5) | / | / |
| **Region** |  |  |  |  |
| Africa | 9 | 30.4 (25.2 , 35.7) | <0.001 | 79.6 |
| Asia | 33 | 20.2 (18.3 , 22.1) | <0.001 | 96 |
| Europe | 5 | 22.4 (14.7 , 30.1) | <0.001 | 94.7 |
| South America | 1 | 29.0 (25.1 , 33.0) | / | / |
| **Study Quality** |  |  |  |  |
| High | 28 | 22.2 (20.1 , 24.3) | <0.001 | 95.1 |
| Moderate / Low | 20 | 22.2 (18.9 , 25.5) | <0.001 | 95.3 |
| **Sample Size** |  |  |  |  |
| ≥1000 | 13 | 19.6 (16.8 , 22.3) | <0.001 | 97.6 |
| <1000 | 35 | 23.6 (20.8 , 26.3) | <0.001 | 93.1 |

**Table S6** Sex(Female) Subgroup Analysis Results of HUA prevalence

| **Subgroup** | **No. of cohorts** | **HUA Prevalence (95%CI) (%)** | **P for heterogeneity** | **I2%** |
| --- | --- | --- | --- | --- |
| **Female** | 45 | 23.1 (19.0 , 27.3) | <0.001 | 99.2 |
| **Diagnostic Criteria** |  |  |  |  |
| > 7.0 mg/dL | 11 | 14.3 (11.0 , 17.6) | <0.001 | 95.7 |
| > 6.0 mg/dL | 31 | 25.6 (20.5 , 30.7) | <0.001 | 99.1 |
| > 6.8 mg/dL | 2 | 26.5 (22.1 , 30.8) | 0.394 | 0.0 |
| ≥6.5 mg/dL | 1 | 14.5 (19.0 , 27.3) | <0.001 | 99.2 |
| **Region** |  |  |  |  |
| Africa | 9 | 34.9 (28.0 , 41.8) | <0.001 | 86.4 |
| Asia | 31 | 20.3 (15.2 , 25.3) | <0.001 | 99.4 |
| Europe | 4 | 22.6 (11.3 , 33.9) | <0.001 | 95.6 |
| South America | 1 | 12.1 (8.5 , 15.8) | / | / |
| **Study Quality** |  |  |  |  |
| High | 26 | 22.6 (16.7 , 28.6) | <0.001 | 99.5 |
| Moderate / Low | 19 | 23.4 (18.9 , 27.9) | <0.001 | 96.8 |
| **Sample Size** |  |  |  |  |
| ≥1000 | 11 | 24.0 (14.9 , 33.0) | <0.001 | 99.8 |
| <1000 | 34 | 22.5 (19.0 , 26.1) | <0.001 | 96.0 |

**Table S7** Meta-regression Results of HUA prevalence

| **Covariates** | **No. of studies** | **Coefficient (95% CI)** | **P-value** | **Adj R-squared** |
| --- | --- | --- | --- | --- |
| **Sample size** | 76 | -0.000008 (-0.00005 , 0.00003) | 0.690 | -1.21% |
| **Age** | 72 | 0.003 (-0.017, 0.022) | 0.778 | -1.41% |
| **Male(n%)** | 74 | 0.232 (-0.550, 1.015) | 0.556 | -1.10% |
| **Year** | 76 | 0.005 (-0.018, 0.028) | 0.677 | -1.24% |
| **Region** | 76 | - | 0.010 | 11.25% |
| Asia |  | Reference |  |  |
| Africa |  | 0.522 (0.225, 0.819) | 0.001 |  |
| Europe |  | 0.048 (-0.308, 0.405) | 0.788 |  |
| South America |  | -0.145 (-0.892, 0.602) | 0.701 |  |
| North America |  | 0.526 (-0.171, 1.223) | 0.137 |  |
| **Diagnosis criteria** | 76 | - | 0.364 | 0.96% |
| Sex-specific |  | Reference |  |  |
| > 7.0 |  | -0.21 (-0.49, 0.08) | 0.151 |  |
| > 6.8 |  | 0.36 (-0.27, 0.99) | 0.260 |  |
| Other criteria |  | -0.13 (-0.60, 0.33) | 0.576 |  |
| Clinical Diagnosis |  | 0.09 (-1.44, -1.12) | 0.731 |  |
| **Study quality** | 76 | - | 0.193 | 1.06% |
| High |  | Reference |  |  |
| Moderate/Low |  | 0.16 (-0.08 , 0.40) | 0.193 |  |
| **ULT treatment** | 76 | - | 0.794 | 0%* |
| Excluded |  | Reference |  |  |
| Not excluded |  | 0.03 (-0.23 , 0.30) | 0.794 |  |

**Table S8** Summary of Non-pooled Risk Factors of HUA

| **Study** | **Risk Factor** |  | **OR (95%CI)** |
| --- | --- | --- | --- |
| Arersa 2020 | Age | 40-49 vs <40 | 0.63 (0.15 - 2.61) |
| Arersa 2020 | Age | 50-59 vs <40 | 1.26 (0.45 - 3.60) |
| Arersa 2020 | Age | ≥60 vs <40 | 2.07 (0.74 - 5.82) |
| Woyesa 2017 | Age | ≥45 vs <45 | 1.90 (1.16 - 3.10) |
| Eljaaly 2021 | WHR | Male≥90, Female≥85 | 0.798 (0.254 – 2.501) |
| Lu 2023 (Middle-aged) | WHR | Per 0.05 increase of WHR | 0.85 (0.68 – 1.08) |
| Lu 2023 (Elderly) | WHR | Per 0.05 increase of WHR | 1.11 (0.89 – 1.38) |
| Lu 2023 (Middle-aged) | WHtR | Per 0.04 increase of WHtR | 1.13 (0.86 – 1.49) |
| Lu 2023 (Elderly) | WHtR | Per 0.04 increase of WHtR | 1.32 (1.06 – 1.64) |
| Chen 2024 a | Antidiabetic Drugs | Non-use vs Use | 0.75 (0.65 – 0.86) |
| Yang 2024 | Insulin |  | 1.13 (1.02 – 1.25) |
| Abudureyimu 2023 | ALB | Per 1g/L increase of ALB | 1.211 (1.121 – 1.308) |
| Wang 2025 | UACR | Per 1 unit increase of UACR | 1.001 (1 – 1.002) |
| Liu 2025 | AST | Per 1 U/L increase of AST | 1.013 (1.002 – 1.023) |
| Abudureyimu 2023 | Ethnic | Other ethnicity vs The Han ethnicity | 3.445 (1.918 – 6.188) |
| Woldeamlak 2019 | Family History of DM |  | 1.5 (1.2 – 3.5) |
| Sheikhbahaei 2014 | HOMA-IR | High vs Normal | 1.39 (0.88 – 2.21) |
| Huang 2025 | MHR |  | 2.04 (1.023 – 4.071) |
| Wang 2024 a | MHR | Per 1 unit increase of MHR | 1.32 (1.14 – 1.53) |
| Wang 2024 a | PrFT | Per 1 mm increase PrFT | 1.24 (1.19 – 1.3) |
| Arersa 2020 | Family History of CVD |  | 2.929 (1.124 – 7.63) |
| Arersa 2020 | Chat chewing |  | 1.475 (0.69 – 3.154) |
| Tang 2025 b | eGDR | Per 1 SD increase of eGDR | 0.83 (0.72 – 0.95) |
| Tang 2025 b | eGDR | T2 vs T1 | 0.84 (0.61 – 1.15) |
| Tang 2025 b | eGDR | T3 vs T1 | 0.68 (0.48 – 0.95) |
| Liu 2025 | Log eGDR | Per 1 increase of Log eGDR | 0.279 (0.17 - 0.459) |
| Kim 2012 | VFA | >143 vs <93 | 2.33 (1.21 - 4.5) |
| Kim 2012 | VFA | 93-143 vs <93 | 1.5 (0.76 - 2.97) |
| Abujbara 2022 | ACEI / ARB drug use |  | 1.68 (1.12 – 2.5) |
| Abujbara 2022 | β-blocker drug use |  | 2.2 (1.51 – 3.22) |
| Abujbara 2022 | Family History of HUA |  | 2.56 (1.57 – 4.16) |
| Peng 2025 | CHD |  | 1.225 (0.817 – 1.837) |
| Peng 2025 | WBC | Per 1*10^9/L increase of WBC | 1.185 (1.077 – 1.303) |
| Yang 2024 | Apo-A | > 0.99 vs ≤ 0.99 | 1.09 (0.97 – 1.22) |
| Yang 2024 | Apo-B | > 4.00 vs ≤ 4.00 | 1.04 (0.92 – 1.17) |
| Yang 2024 | Lp(a) | > 3.79 vs ≤ 3.79 | 0.99 (0.87 – 1.13) |
| Yang 2024 | Cardiovascular drugs |  | 1.29 (1.14 – 1.47) |
| Yang 2024 | Electrolytes, acid–base balancing drugs |  | 1.07 (0.89 – 1.27) |
| Yang 2024 | Adjuvant drugs for liver disease |  | 1.70 (1.45 – 2.00) |
| Yang 2024 | Antipodagrics |  | 1.20 (1.00 – 1.45) |
| Yang 2024 | Antiplatelet drug |  | 1.08 (0.88 – 1.33) |
| Yang 2024 | Antithyroid drugs |  | 3.84 (3.25 – 4.53) |
| Qin 2021 | UEGL | Q2 vs Q1 | 0.45 (0.22 – 0.9) |
| Qin 2021 | UEGL | Q3 vs Q1 | 0.47 (0.23 – 0.96) |
| Qin 2021 | UEGL | Q4 vs Q1 | 0.28 (0.12 – 0.63) |
| Choukem 2016 | DR |  | 2.4 (1.3 – 4.5) |
| Choukem 2016 | Hydrochlorothiazide |  | 3.2 (1.3 – 8.1) |
| Liu 2025 | Hb |  | 0.981 (0.97 – 0.992) |
| Wang 2025 | CVAI | Per 1 unit increase | 1.011 (1.002 – 1.02) |
| Wang 2025 | CVAI | Q2 vs Q1 | 2.688 (1.301 – 5.554) |
| Wang 2025 | CVAI | Q3 vs Q1 | 2.752 (1.32 – 5.739) |
| Wang 2025 | CVAI | Q4 vs Q1 | 4.99 (2.392 – 10.409) |
| Lin 2024 | Remnant Cholesterol | Per 1 unit increase | 1.65 (1.42 – 1.92) |
| Lin 2024 | Remnant Cholesterol | Q2 vs Q1 | 0.93 (0.64 – 1.33) |
| Lin 2024 | Remnant Cholesterol | Q3 vs Q1 | 1.71 (1.22 – 2.39) |
| Lin 2024 | Remnant Cholesterol | Q4 vs Q1 | 2.36 (1.71 – 3.26) |
| Woyesa 2017 | Resident | Urban vs Rural | 0.8 (0.5 – 1.3) |
| Woyesa 2017 | Regular Exercise |  | 1.5 (1.33 – 2.45) |
| Chen 2024 a | Exercise |  | 1.08 (0.97 – 1.2) |
| Chen 2024 a | TBIL | ≥ 17.1 vs＜17.1 | 0.97 (0.85 – 1.12) |
| Chen 2024 a | GGT | ≥ 40 vs ＜40 | 1.38 (1.13 – 1.7) |
| Abudureyimu 2023 | IPHOS | Per 1 mmol/L increase | 5.146 (1.098 – 24.125) |
| Abudureyimu 2023 | CO2 | Per 1 mmol/L increase | 0.829 (0.747 – 0.921) |
| Abudureyimu 2023 | CysC | Per 1 mg/L increase | 13.011 (5.229 – 32.374) |
| Abudureyimu 2023 | ALP | Per 1 U/L increase | 0.978 (0.965 – 0.991) |
| Wang 2025 | Fatty Liver |  | 0.969 (0.621 – 1.513) |
| Wang 2025 | C-Peptide | Per 1 ng/mL increase | 1.13 (0.954 – 1.339) |

**Table S9** Summary of Risk Factors of Gout

| **Risk Factor** | **No. of Cohort** | **OR (95%CI)** | **P for heterogeneity** | **I2%** | **P for overall effect** |
| --- | --- | --- | --- | --- | --- |
| **Sex (Male)** | 2 | 2.75 (1.36 - 5.55) | 0.058 | 72.2 | <0.001 |
| **Hypertriglyceridemia** | 2 | 1.45 (0.89 - 2.37) | 0.148 | 52.3 | 0.135 |

**Table S10** Summary of Non-pooled Risk Factors of Gout

| **Study** | **Risk Factor** |  | **OR (95%CI)** |
| --- | --- | --- | --- |
| Cai 2024 | BMI | ≥ 28 | 1.775(1.468–2.145) |
| Cai 2024 | BMI | Q2 vs Q1 | 1.888(1.380–2.582) |
| Cai 2024 | BMI | Q3 vs Q1 | 1.999(1.469–2.721) |
| Cai 2024 | BMI | Q4 vs Q1 | 2.751(2.034–3.720) |
| Cai 2024 | WC | M ≥ 90,F ≥ 85 cm | 1.691(1.394–2.053) |
| Cai 2024 | WC | Q2 vs Q1 | 1.869(1.336–2.614) |
| Cai 2024 | WC | Q3 vs Q1 | 2.139(1.539–2.966) |
| Cai 2024 | WC | Q4 vs Q1 | 2.999(2.185–4.118) |
| Cai 2024 | WHR | M ≥ 0.9,F ≥ 0.85 | 1.858(1.367–2.524) |
| Cai 2024 | WHR | Q2 vs Q1 | 1.462(1.049–2.037) |
| Cai 2024 | WHR | Q3 vs Q1 | 1.834(1.335–2.520) |
| Cai 2024 | WHR | Q4 vs Q1 | 2.127(1.561–2.896) |
| Cai 2024 | VFA | ≥ 100 | 1.578(1.317–1.890) |
| Cai 2024 | VFA | Q2 vs Q1 | 1.266(0.940–1.704) |
| Cai 2024 | VFA | Q3 vs Q1 | 1.360(1.020–1.815) |
| Cai 2024 | VFA | Q4 vs Q1 | 2.259(1.730–2.950) |
| Cai 2025 | CVAI | Per 1 increase | 1.07 (1.05∼1.09) |
| Cai 2025 | CVAI | Q2 vs Q1 | 1.65 (1.23∼2.21) |
| Cai 2025 | CVAI | Q3 vs Q1 | 2.17 (1.64∼2.87) |
| Cai 2025 | CVAI | Q4 vs Q1 | 2.44 (1.85∼3.22) |
| Collier 2016 | Age (decades) |  | 1.14 (1.07 to 1.21) |
| Collier 2016 | No of medications |  | 1.10 (1.06 to 1.14) |
| Collier 2016 | BMI | over 30 | 1.25 (1.11 to 1.40) |
| Collier 2016 | Triglyceride level |  | 1.25 (1.18 to 1.33) |
| Collier 2016 | eGFR stage 3a |  | 1.81 (1.59 to 2.07) |
| Collier 2016 | eGFR stage 3b |  | 3.44 (2.86 to 4.13) |
| Collier 2016 | eGFR stage 4/5 |  | 6.97 (5.37 to 9.04) |
| Collier 2016 | Current smoker |  | 0.64 (0.54 to 0.76) |
| Collier 2016 | Haemoglobin A1c level |  | 0.98 (0.98 to 0.99) |
| Collier 2016 | Low-density lipoprotein cholesterol |  | 0.89 (0.83 to 0.96) |
| Suppiah 2008 | Ethnicity | European vs Asian | 1.0 (0.0 – 1.8) |
| Suppiah 2008 | Ethnicity | Indian vs Asian | 0.6 (0.2 – 1.9) |
| Suppiah 2008 | Ethnicity | Māori vs Asian | 1.2 (0.4 – 3.1) |
| Suppiah 2008 | Ethnicity | Pacific people vs Asian | 1.6 (0.7 – 4.2) |
| Suppiah 2008 | age | Every 10-year increase in age | 1.0 (1.0 – 1.0) |
| Suppiah 2008 | GFR | Every 10 ml/min decrease in GFR | 1.2 (1.1 – 1.4) |
| Suppiah 2008 | Aspirin |  | 1.2 (0.6 – 2.5) |
| Suppiah 2008 | Diuretics |  | 3.2 (1.6 – 6.6) |
| Suppiah 2008 | Low HDL |  | 0.7 (0.3 – 1.4) |

**Figure S1.** Funnel plot of overall HUA prevalence


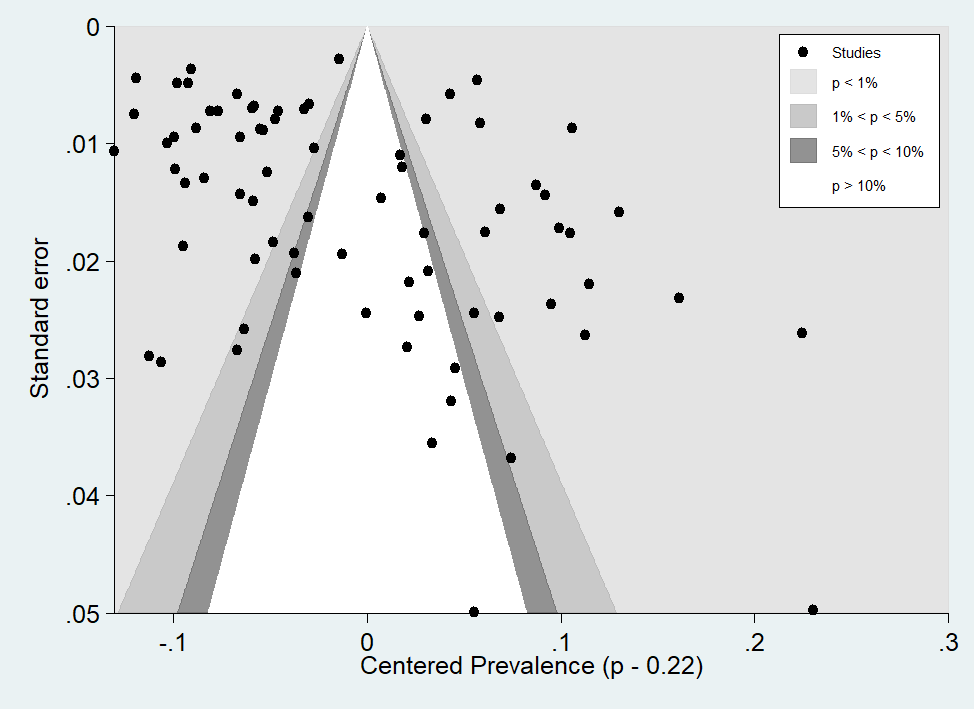


**Figure S2.** Egger's test plot for overall HUA prevalence


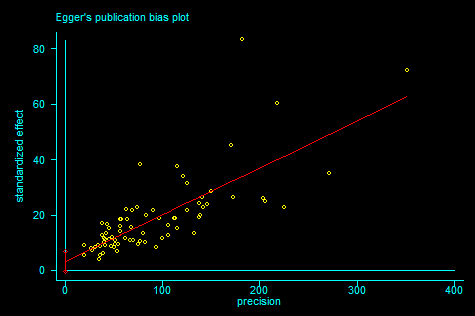


**Figure S3.** Bubble plot of meta-regression of HUA Prevalence

1. Bubble plot of meta-regression of mean age and HUA Prevalence


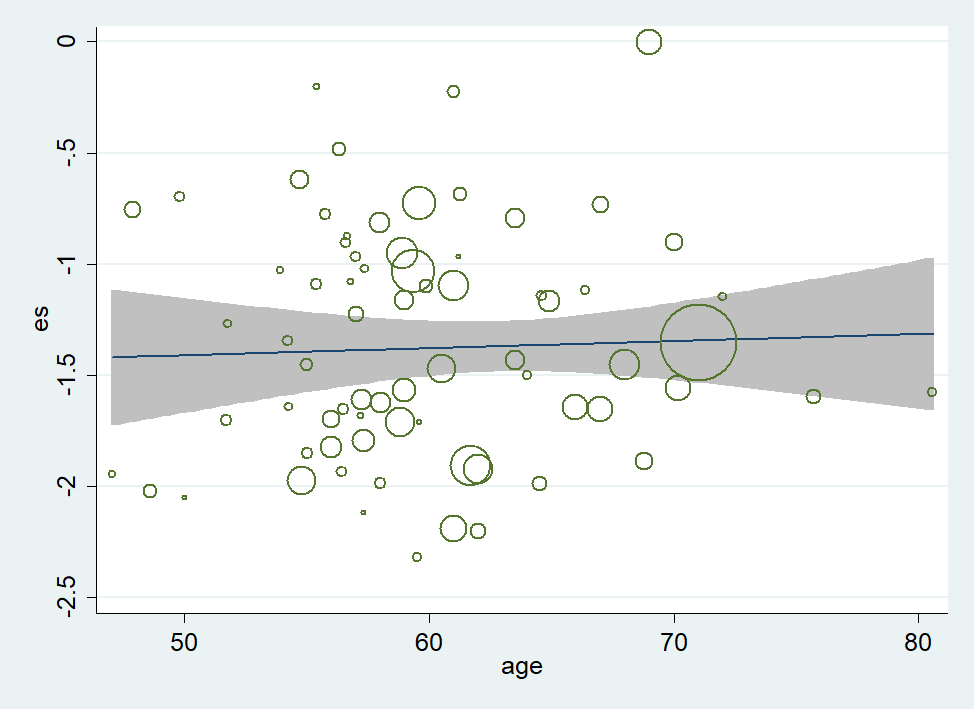


1. Bubble plot of meta-regression of Male(n%) and HUA Prevalence


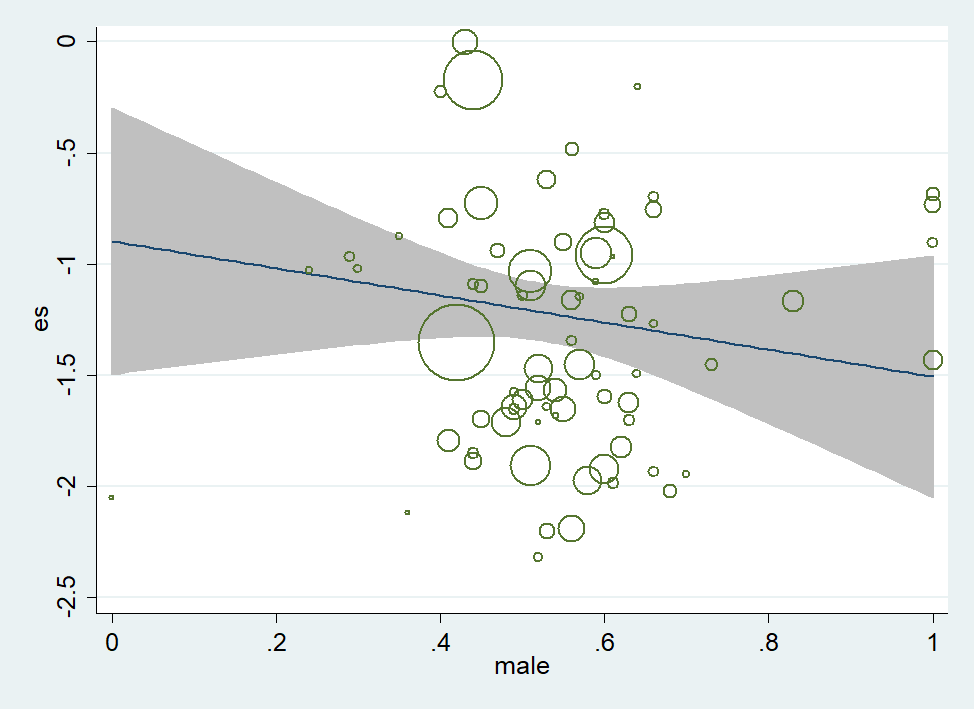


1. Bubble plot of meta-regression of Year and HUA Prevalence


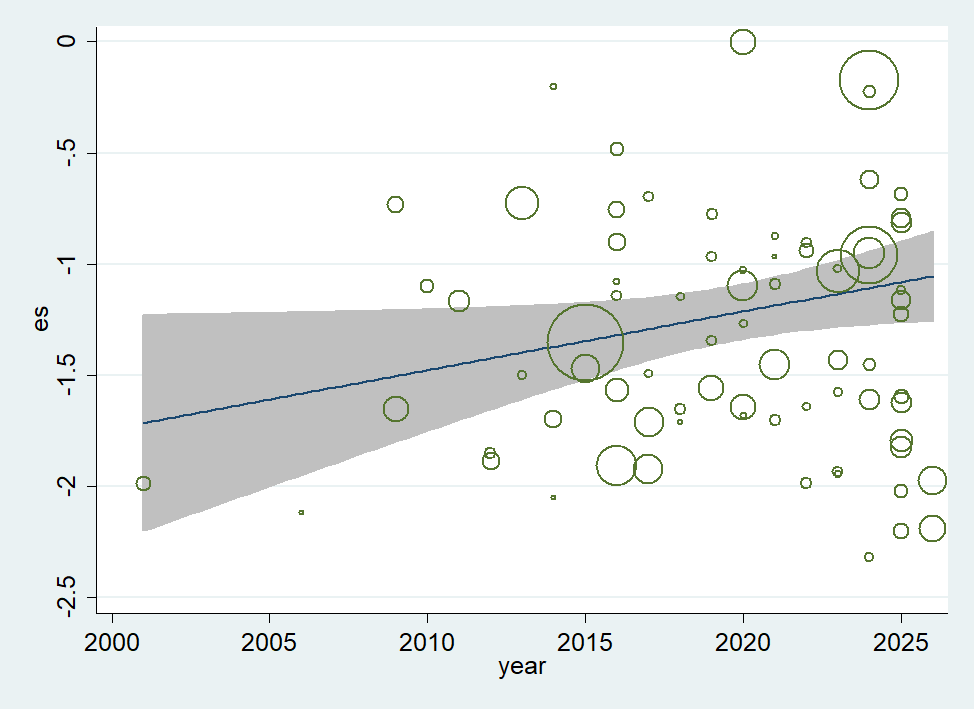


1. Bubble plot of meta-regression of Sample size and HUA Prevalence


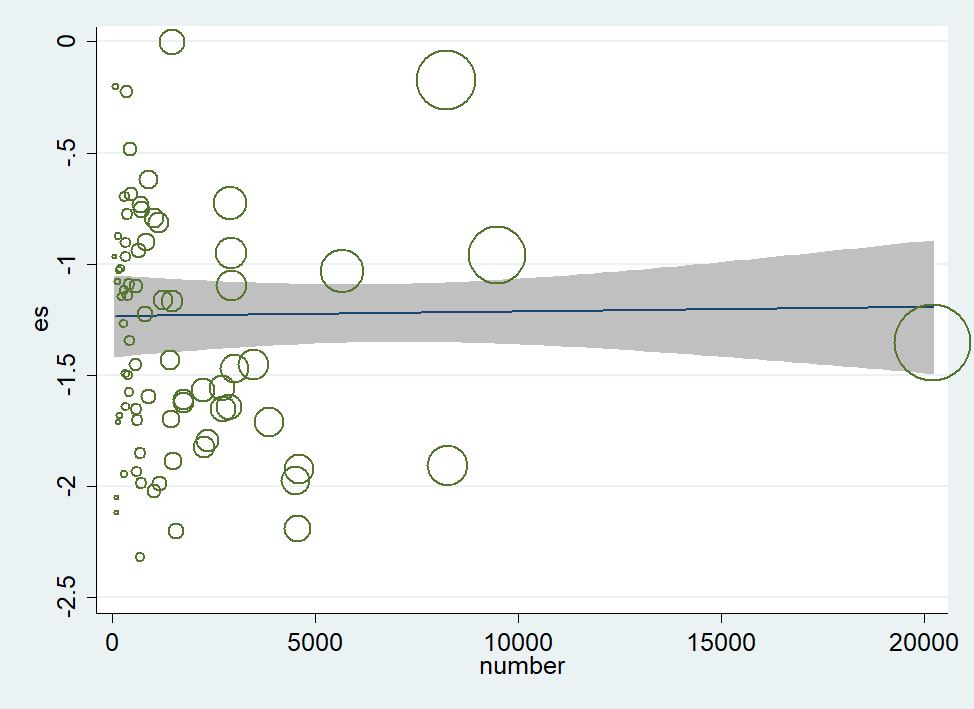


**Figure S4.** Forest plot of HUA prevalence in the Male subgroup


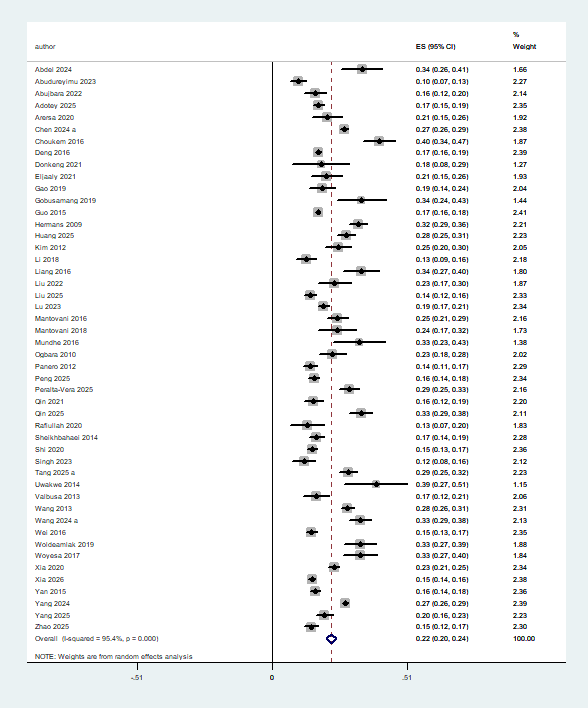


**Figure S5.** Forest plot of HUA prevalence in the Female subgroup


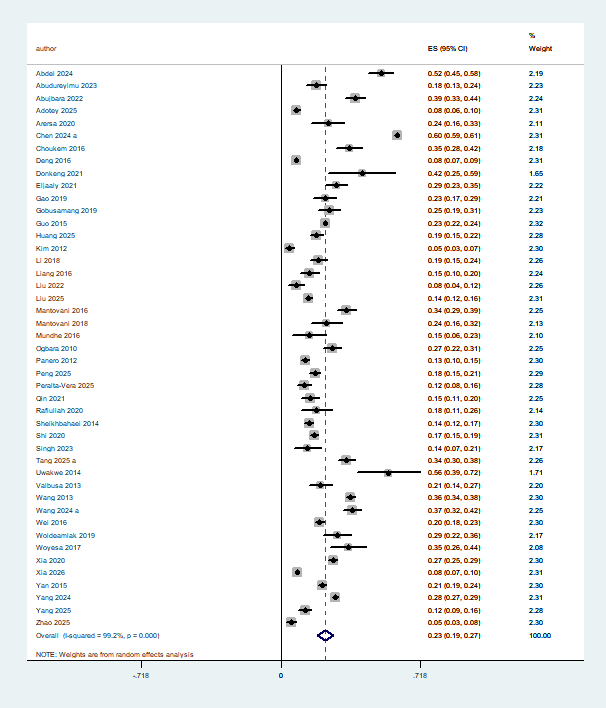


**Figure S6.** Forest plot of HUA prevalence by age


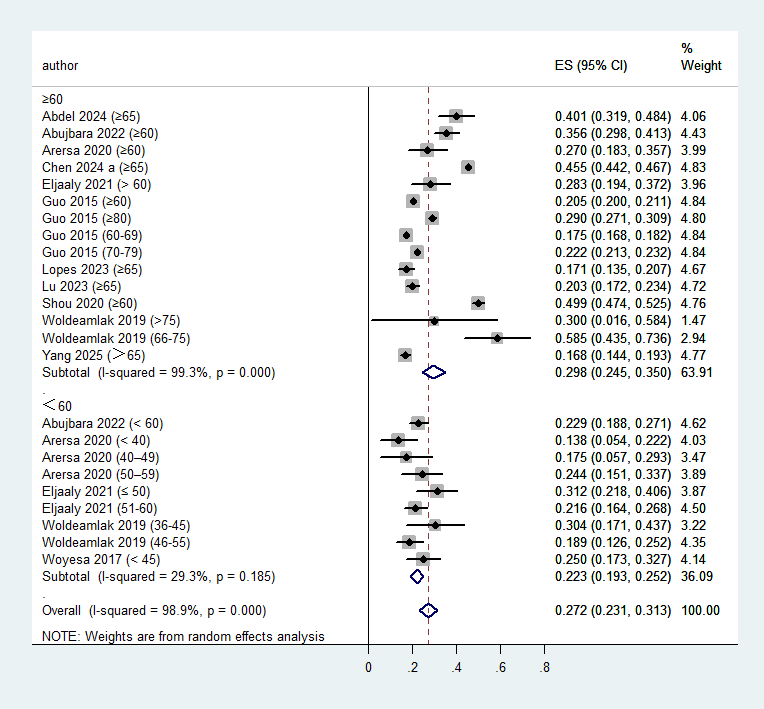


**Figure S7.** Forest plot of HUA prevalence by smoking status
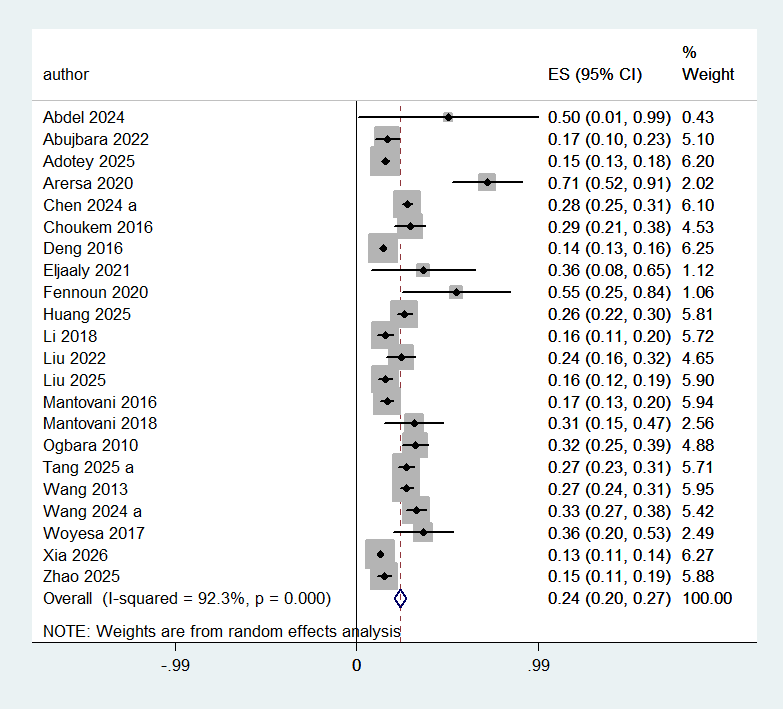


**Figure S8.** Forest plot of HUA prevalence by alcohol consumption


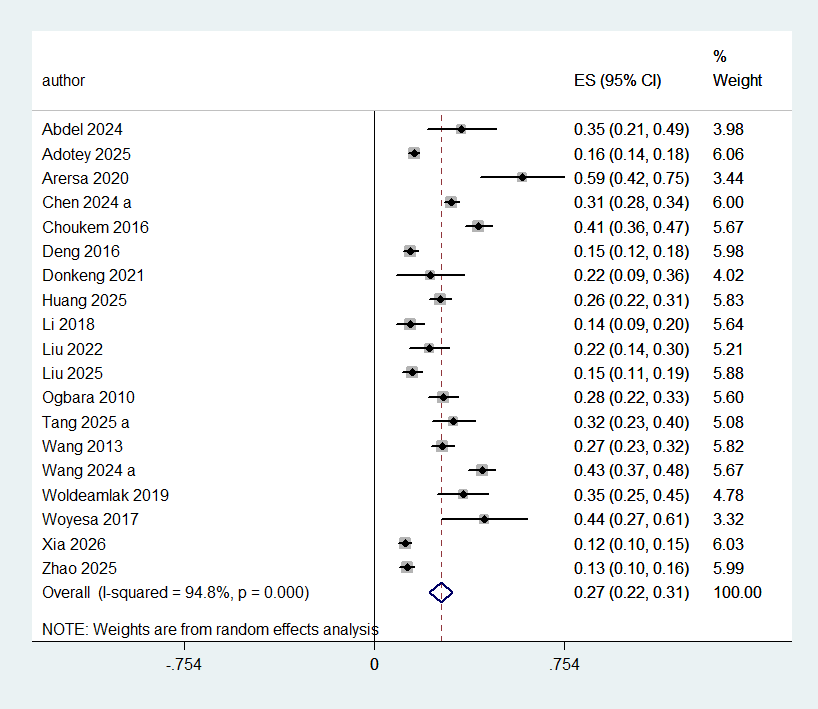


**Figure S9.** Forest plot of HUA prevalence by diabetes duration
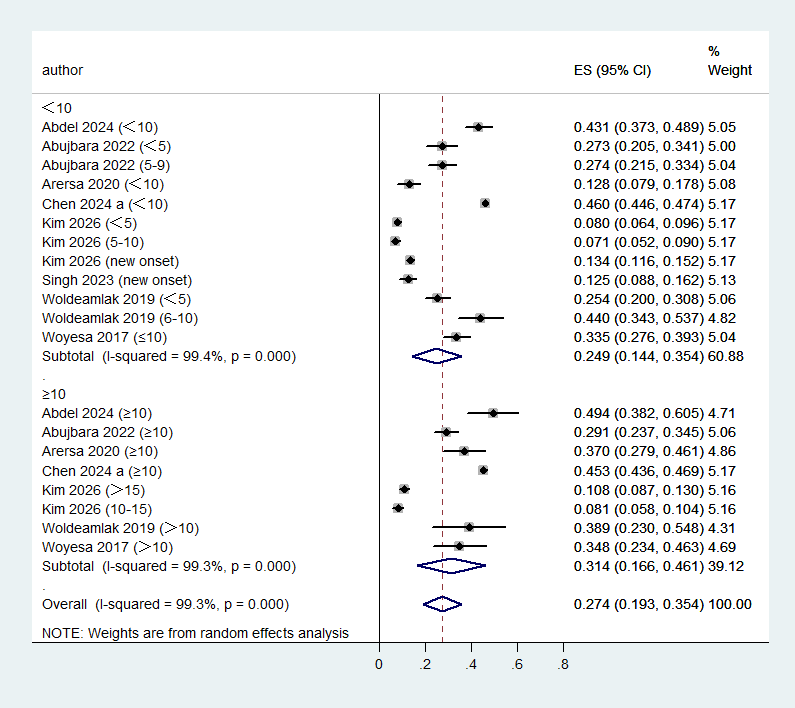


**Figure S10.** Forest plot of HUA prevalence by family history of diabetes


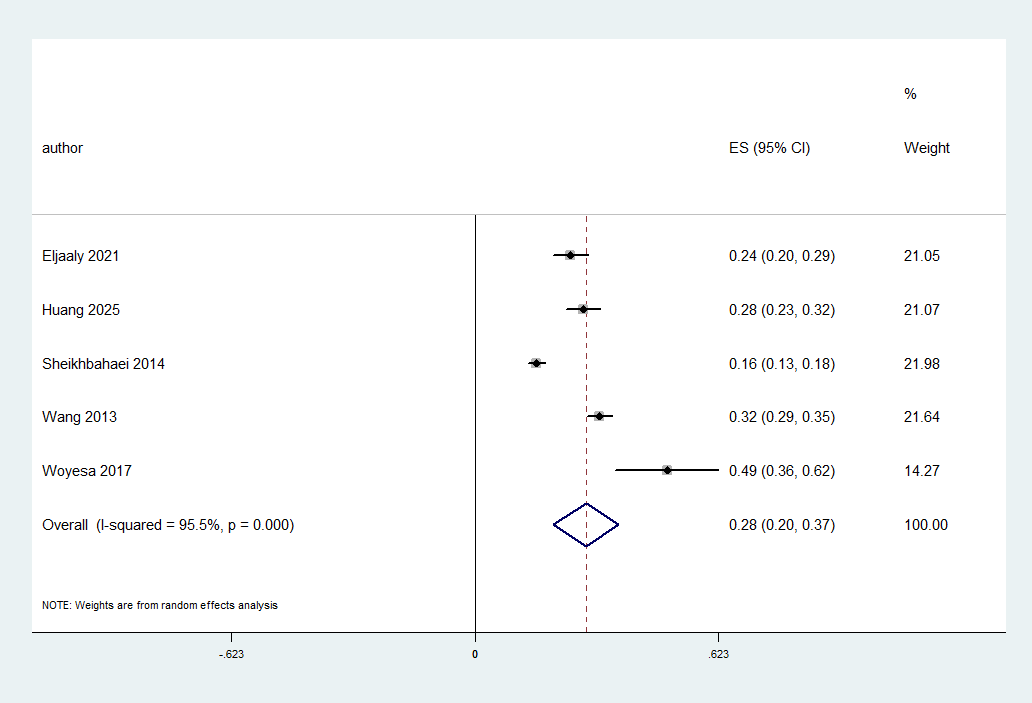


**Figure S11-1.** Forest plot of HUA prevalence in overweight and obesity subgroups


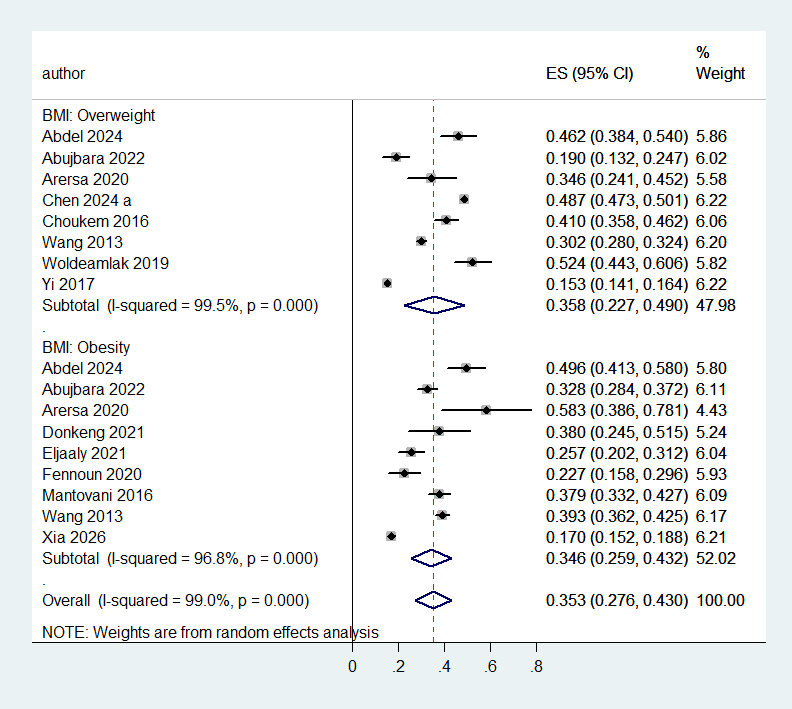


**Figure S11-2.** Forest plot of HUA prevalence in the central obesity subgroup


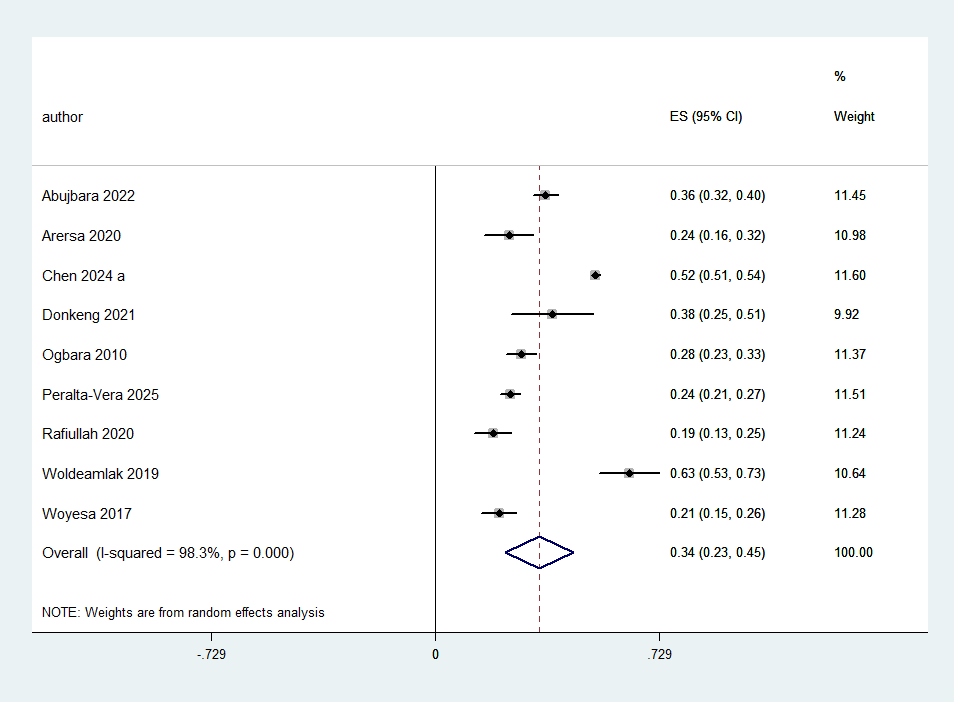


**Figure S12-1.** Forest plot of HUA prevalence in the dyslipidemia subgroup


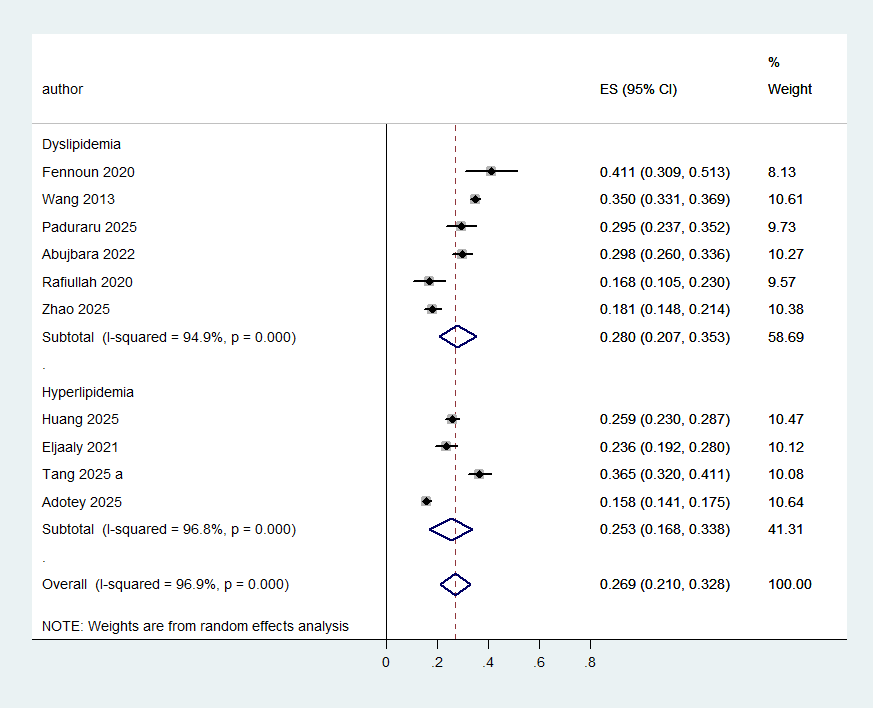


**Figure S12-2.** Forest plot of HUA prevalence in the hypertriglyceridemia subgroup


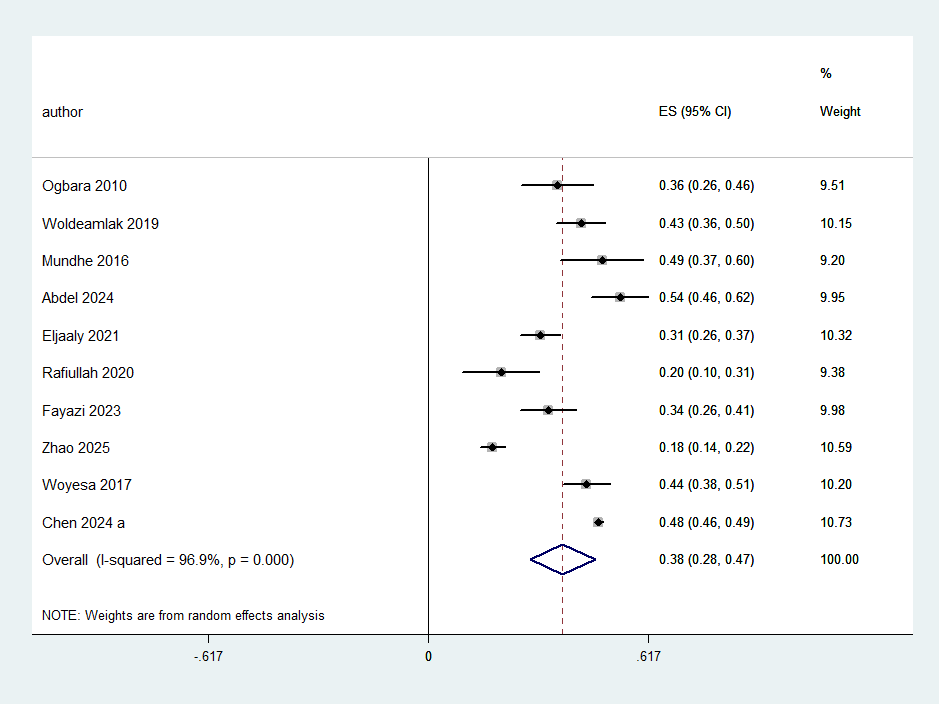


**Figure S12-3.** Forest plot of HUA prevalence in the hypercholesterolemia subgroup

**
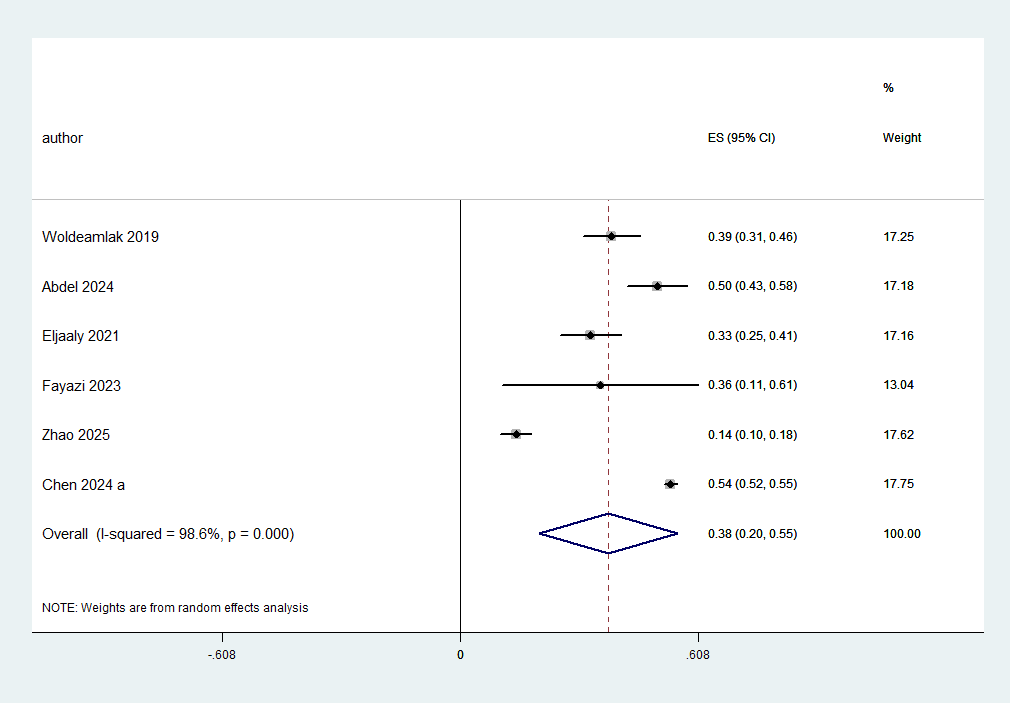
**

**Figure S12-4.** Forest plot of HUA prevalence in the high LDL-C subgroup

**
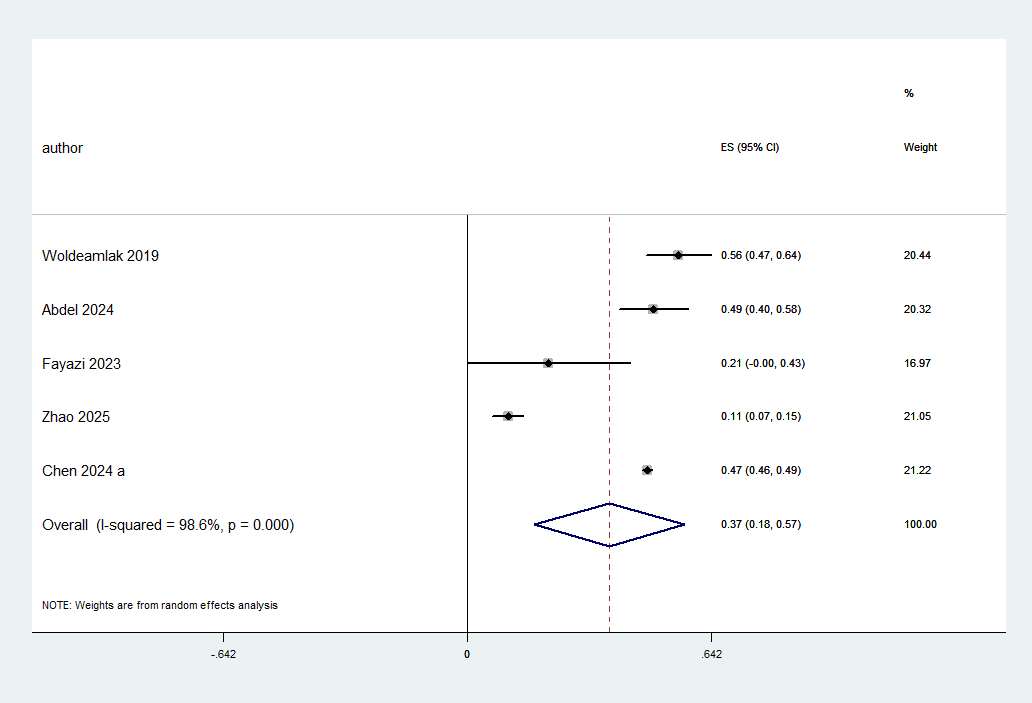
**

**Figure S12-5.** Forest plot of HUA prevalence in the low HDL-C subgroup


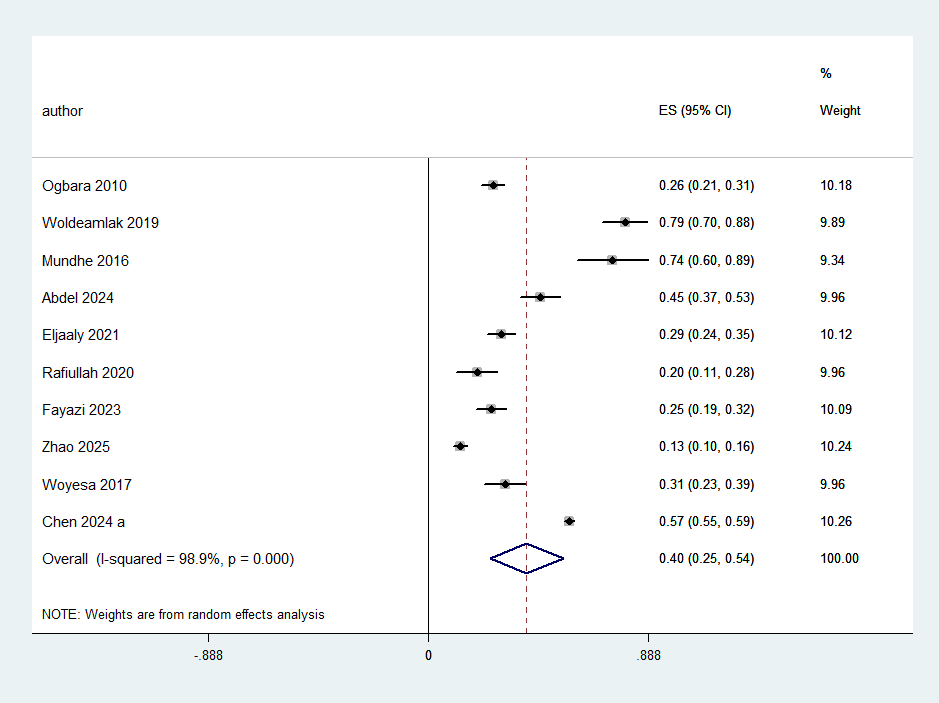


**Figure S13.** Forest plot of HUA prevalence in the metabolic syndrome subgroup


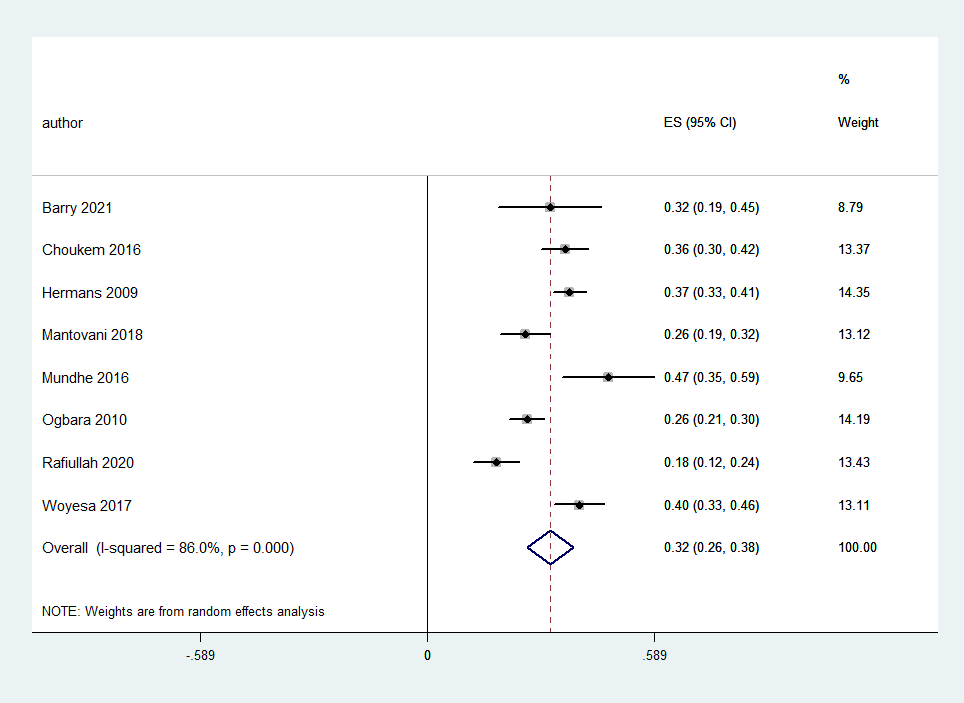


**Figure S14.** Forest plot of HUA prevalence in the hypertension subgroup


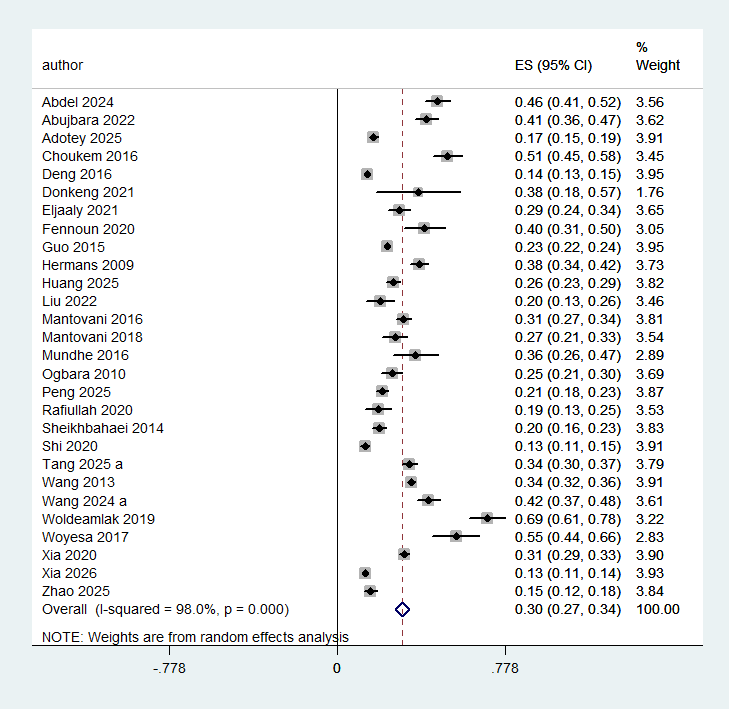


**Figure S15.** Forest plot of HUA prevalence in the renal insufficiency subgroup


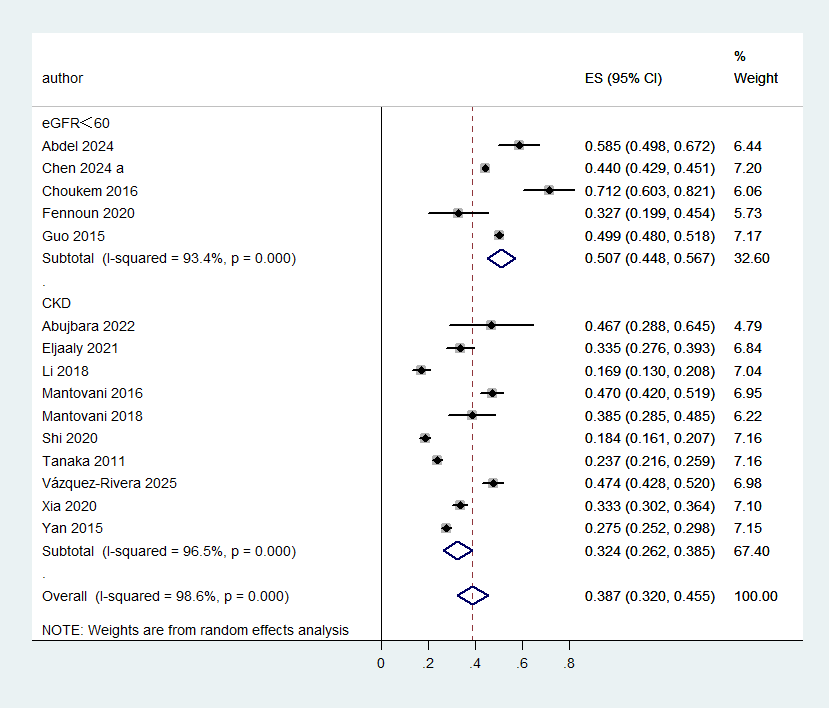


**Figure S16.** Forest plot of HUA prevalence in the ischemic heart disease subgroup


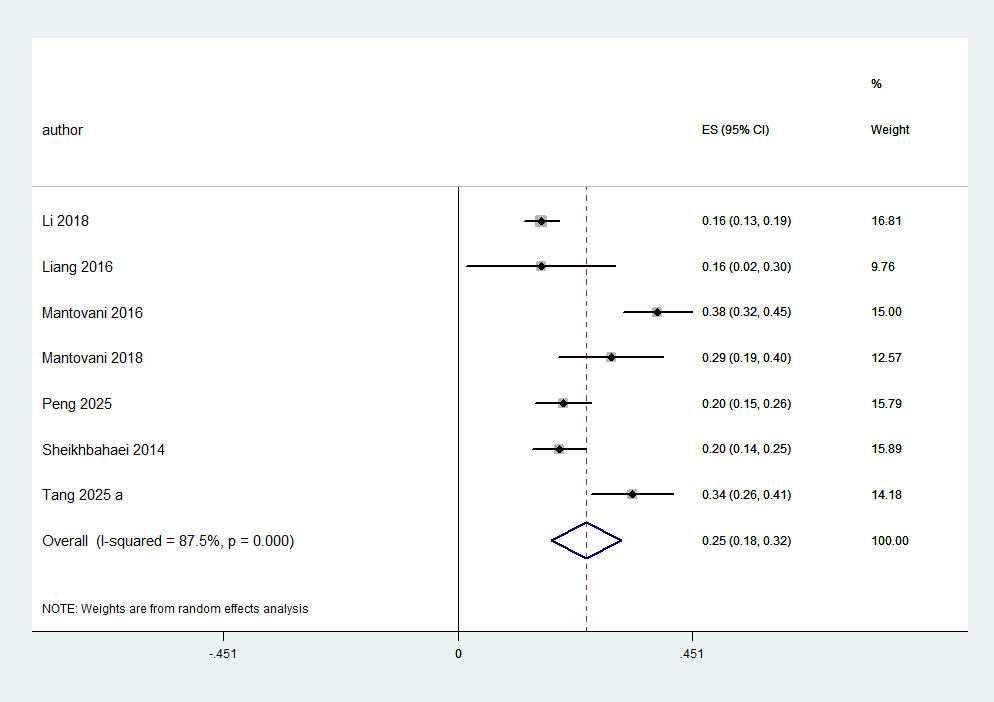


**Figure S17.** Forest plot of HUA prevalence in the diabetic retinopathy subgroup


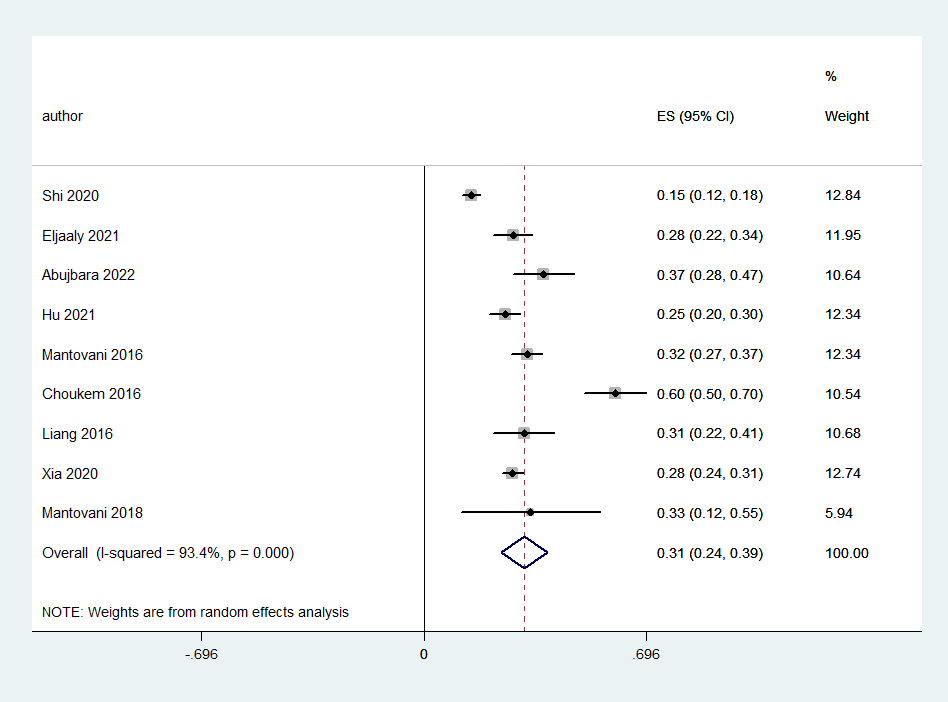


**Figure S18-1.** Forest plot of HUA prevalence in the HbA1c≥7% subgroup


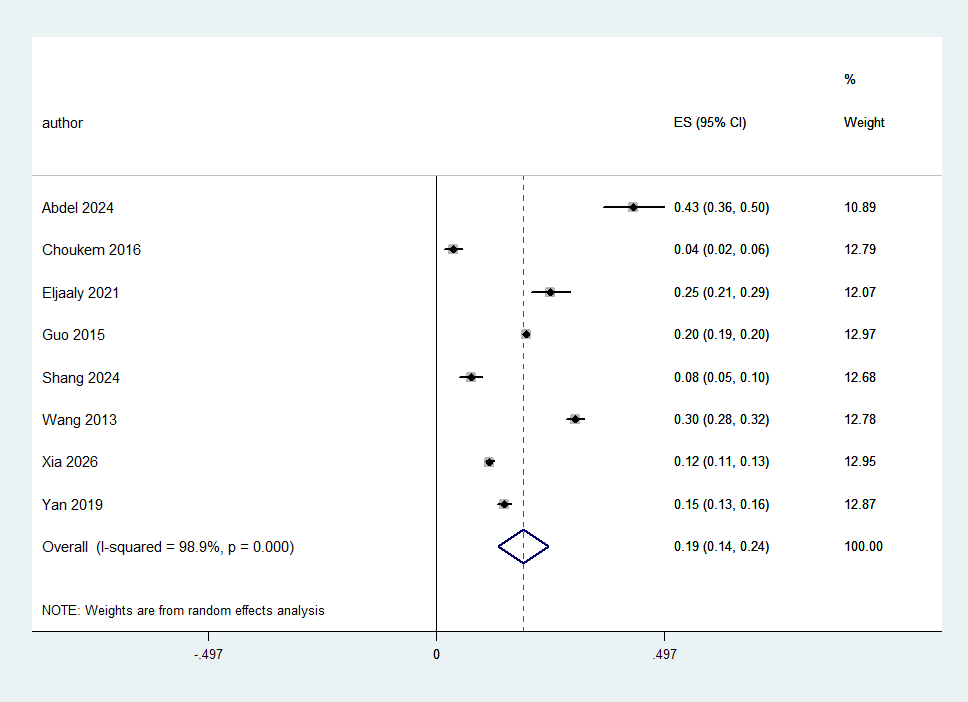


**Figure S18-2.** Forest plot of HUA prevalence in the HbA1c<7% subgroup


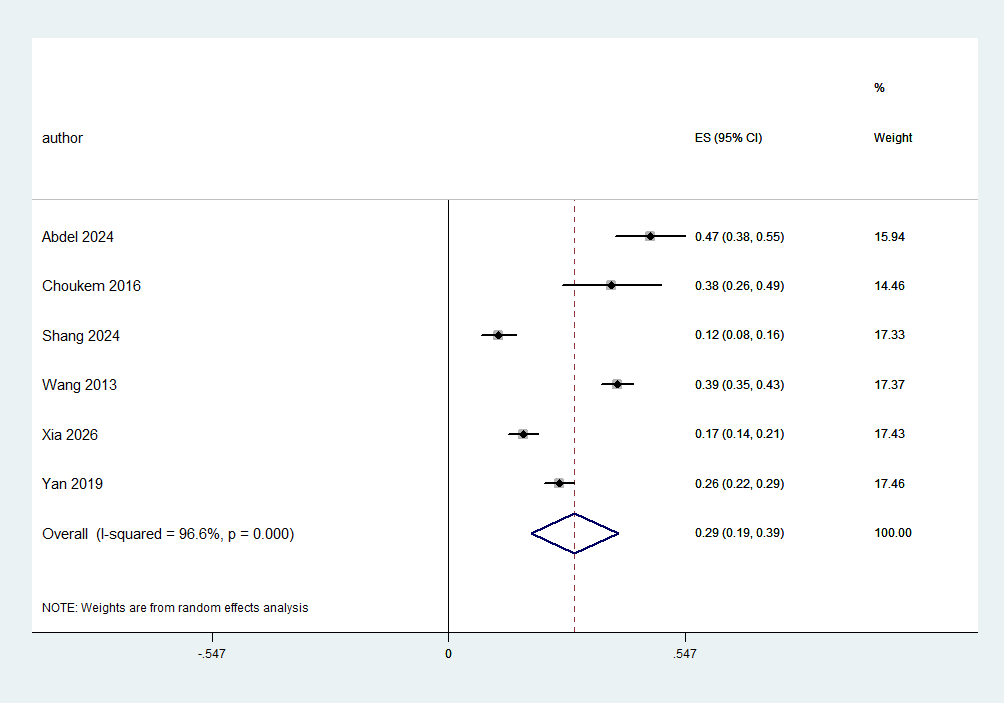


**Figure S19.** Forest plot of HUA prevalence by diuretic use


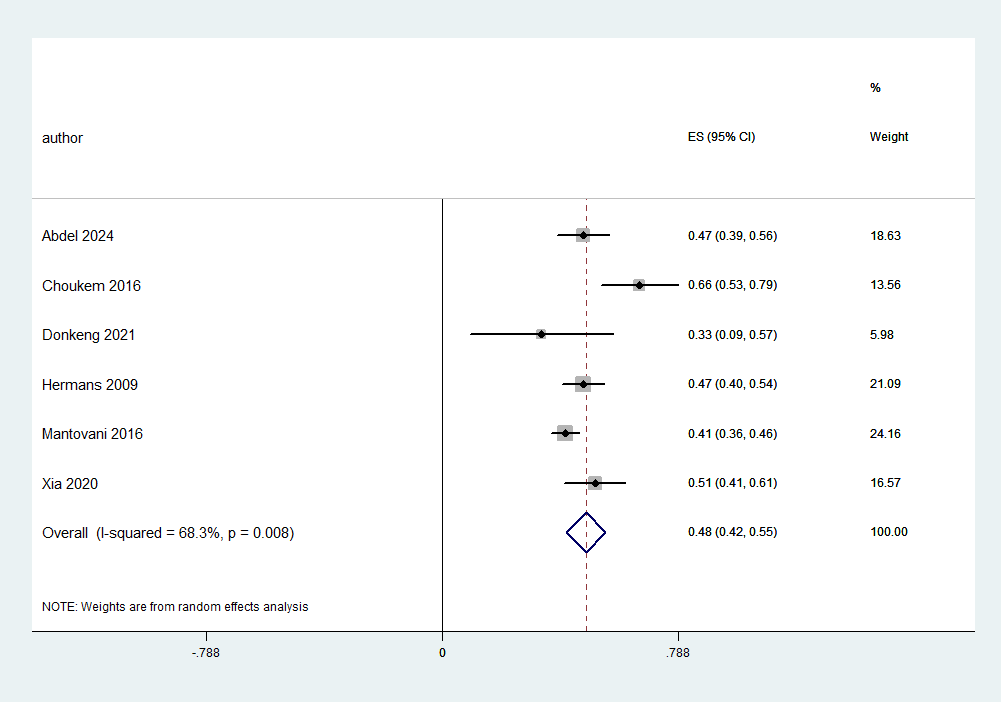


**Figure S20.** Forest plot of HUA prevalence by lipid-lowering drug use


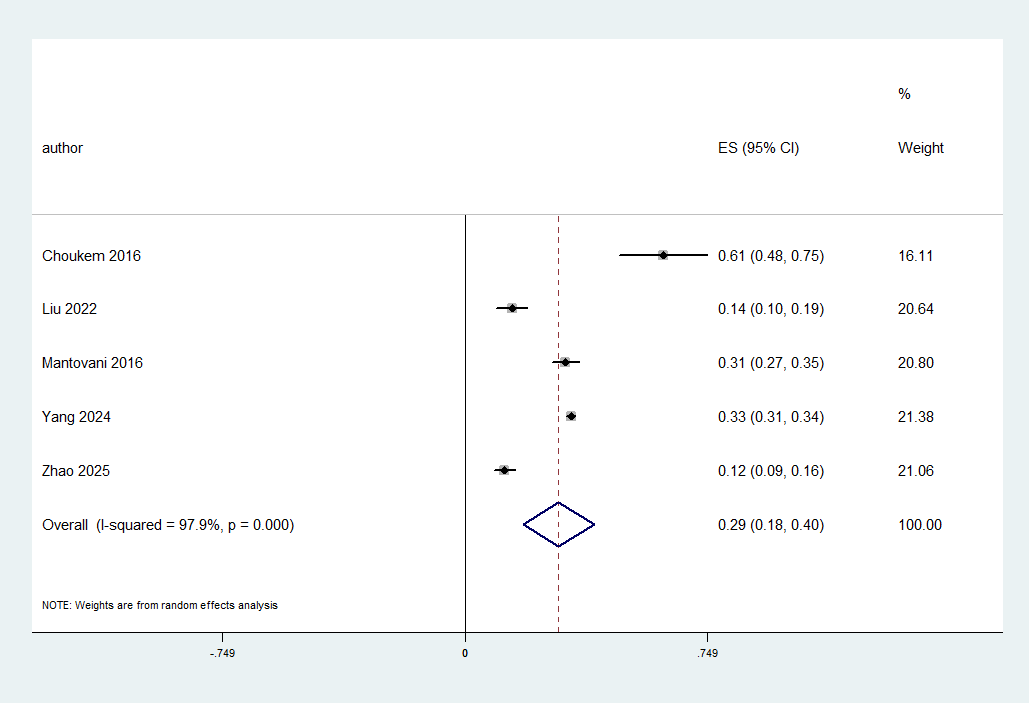


**Figure S21.** Forest plot of HUA prevalence by hypoglycemic drug use


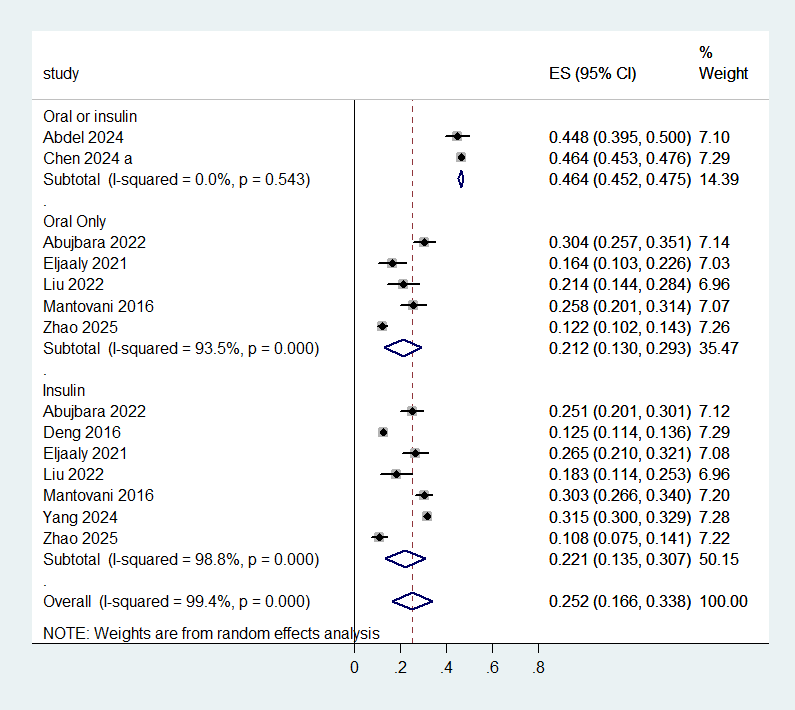


**Figure S22.** Forest plot of overall Gout prevalence


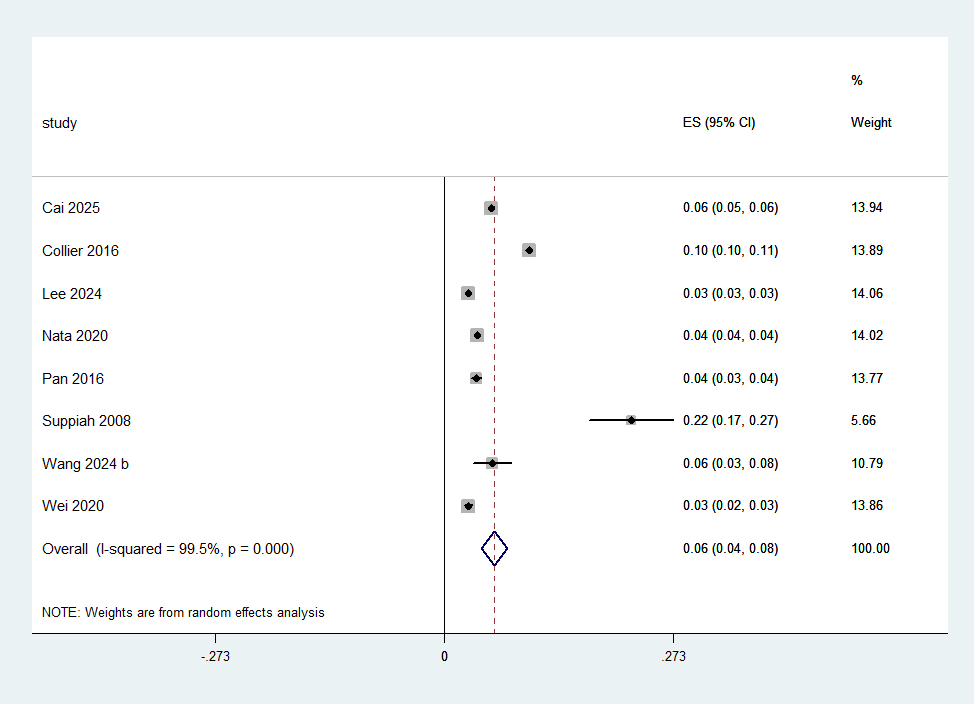


**Figure S23.** Funnel plot of overall Gout prevalence


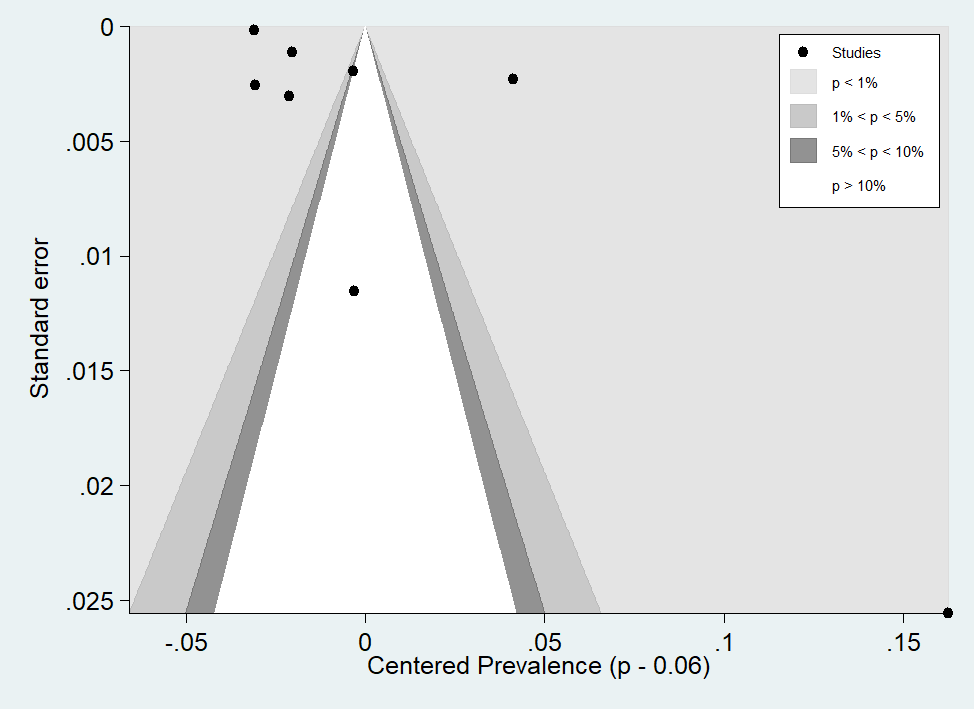


**Figure S24.** Publication bias assessment for Gout prevalence


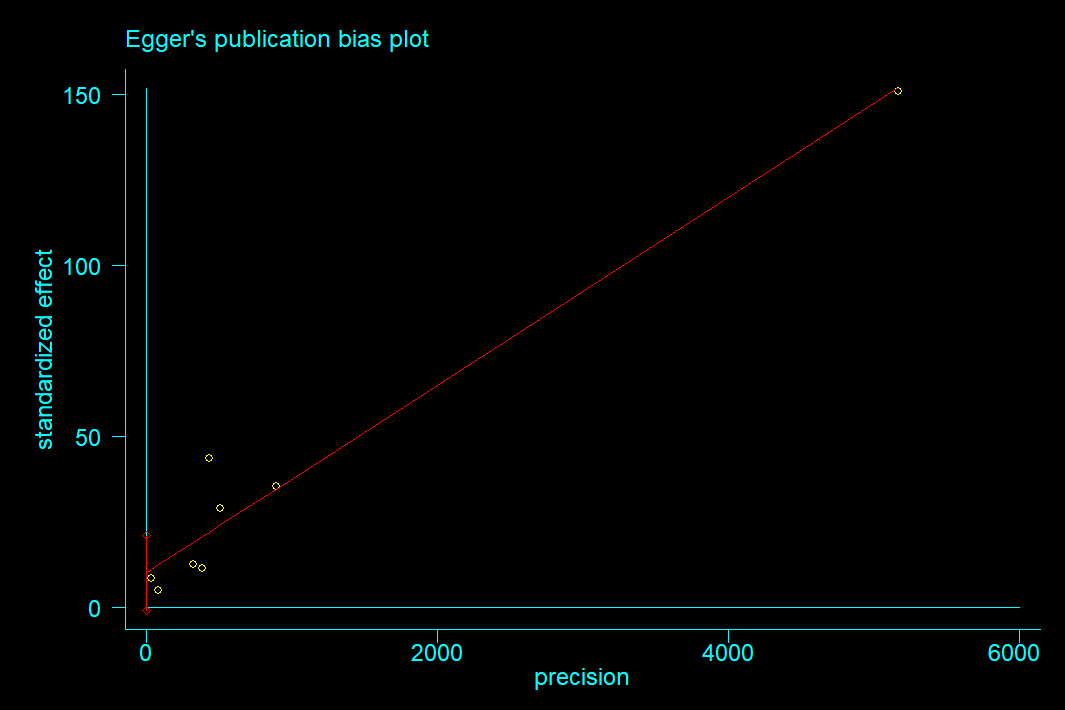


**Figure S25.** Sensitivity analysis of overall Gout prevalence


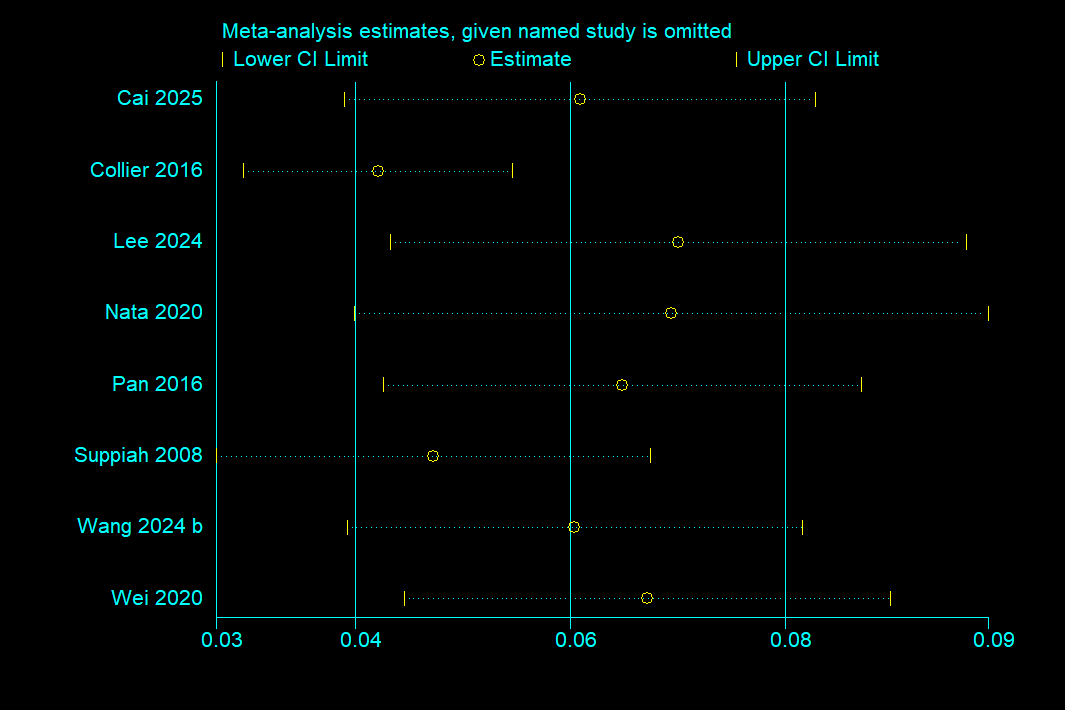


**Figure S26.** Forest plot of Gout prevalence by region


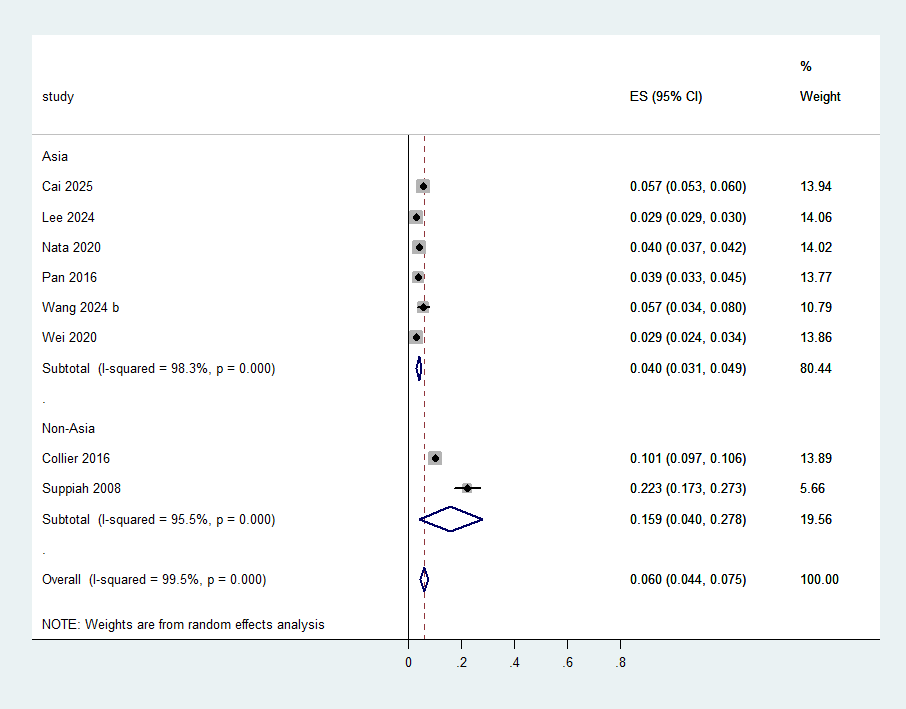


**Figure S27.** Forest plot of Gout prevalence by diagnostic criteria


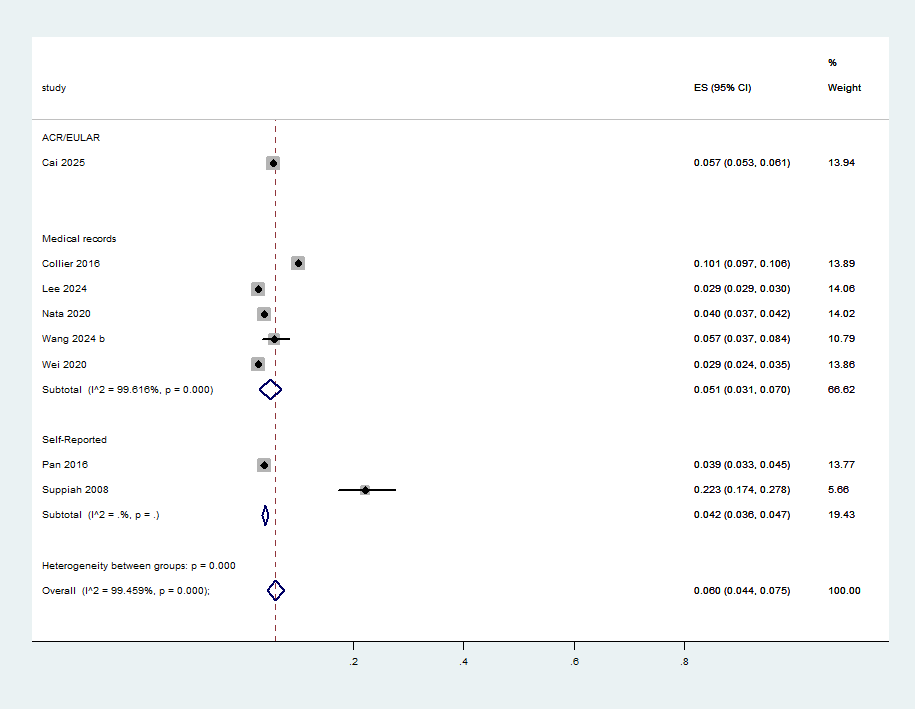


**Figure S28.** Forest plot of Gout prevalence by sample size


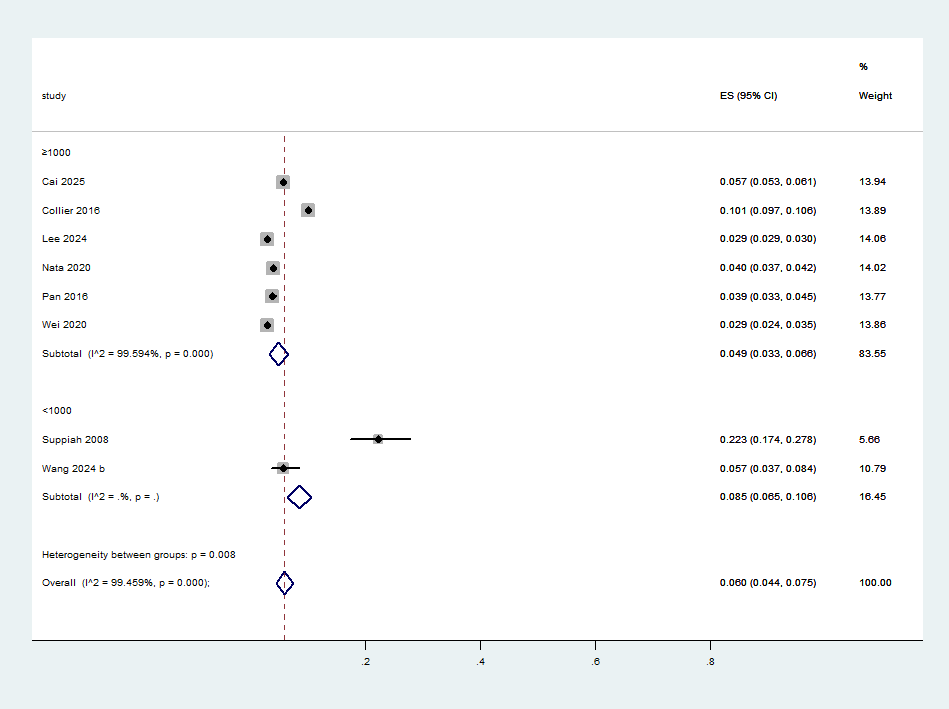


**Figure S29.** Forest plot of Gout prevalence by study quality


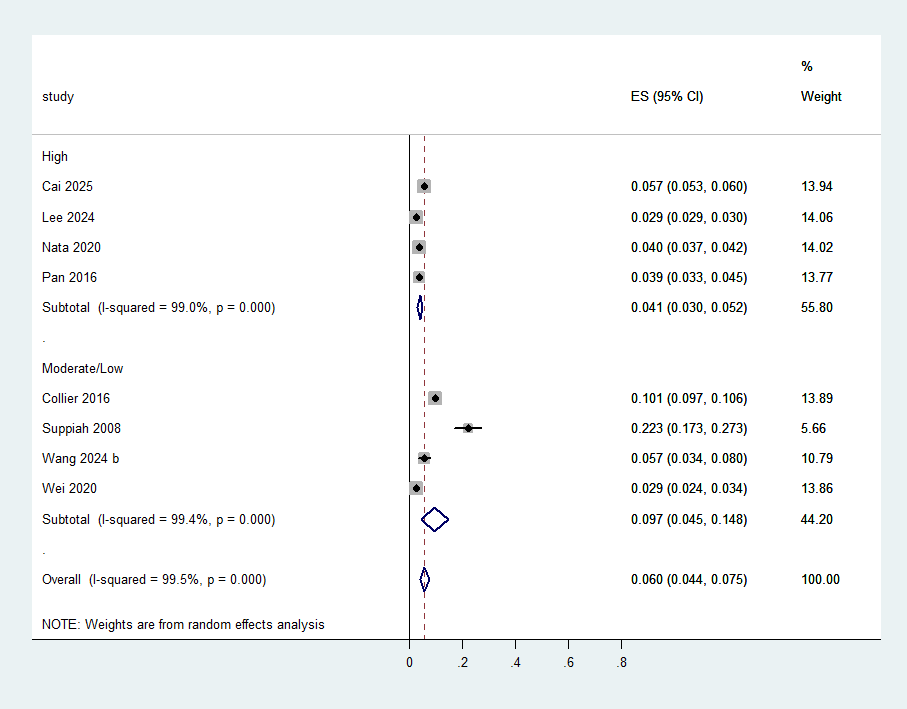


**Figure S30.** Forest plot of Gout prevalence in the male subgroup


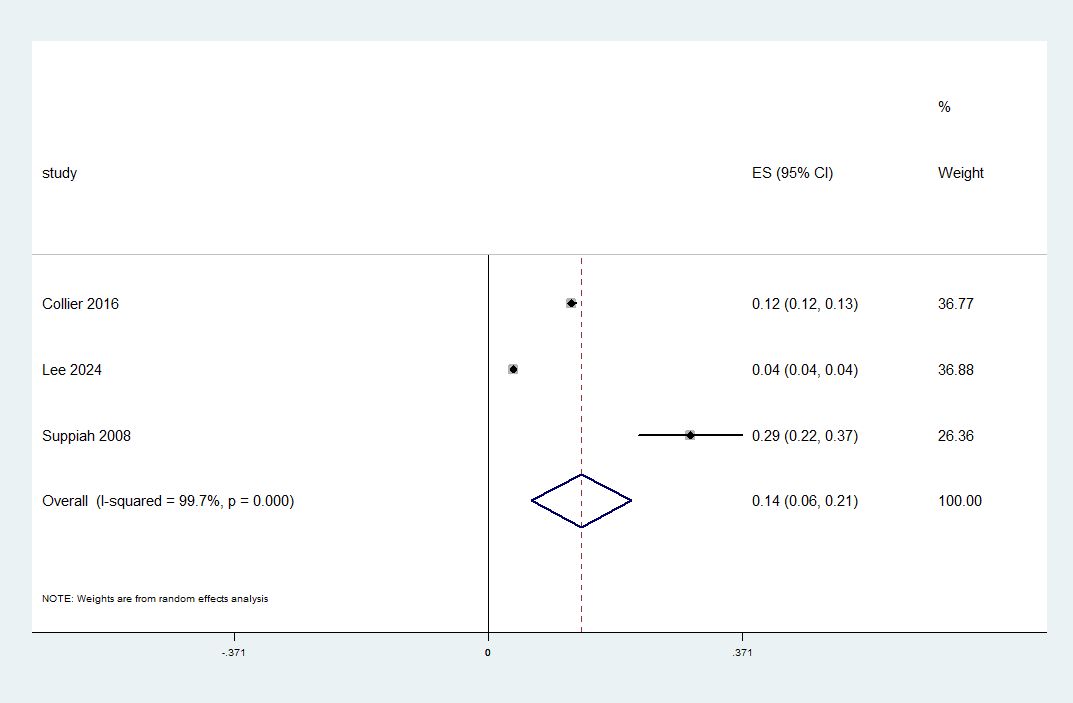


**Figure S31.** Forest plot of Gout prevalence in the female subgroup


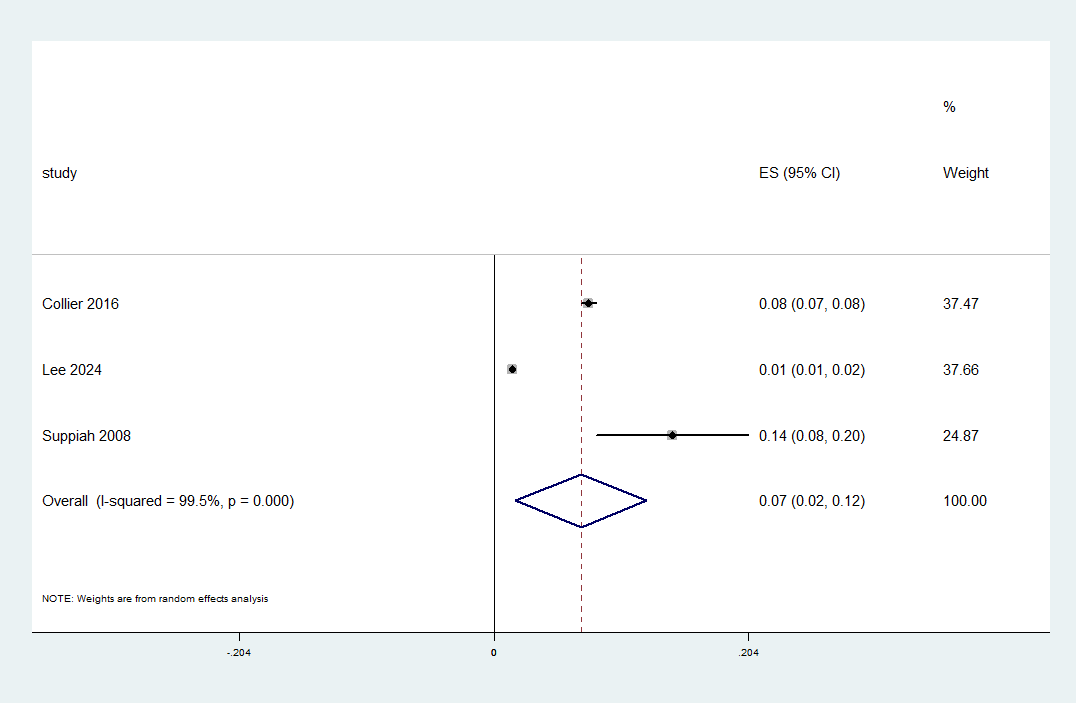


**Figure S32.** Forest plot of Gout prevalence in the CKD subgroup


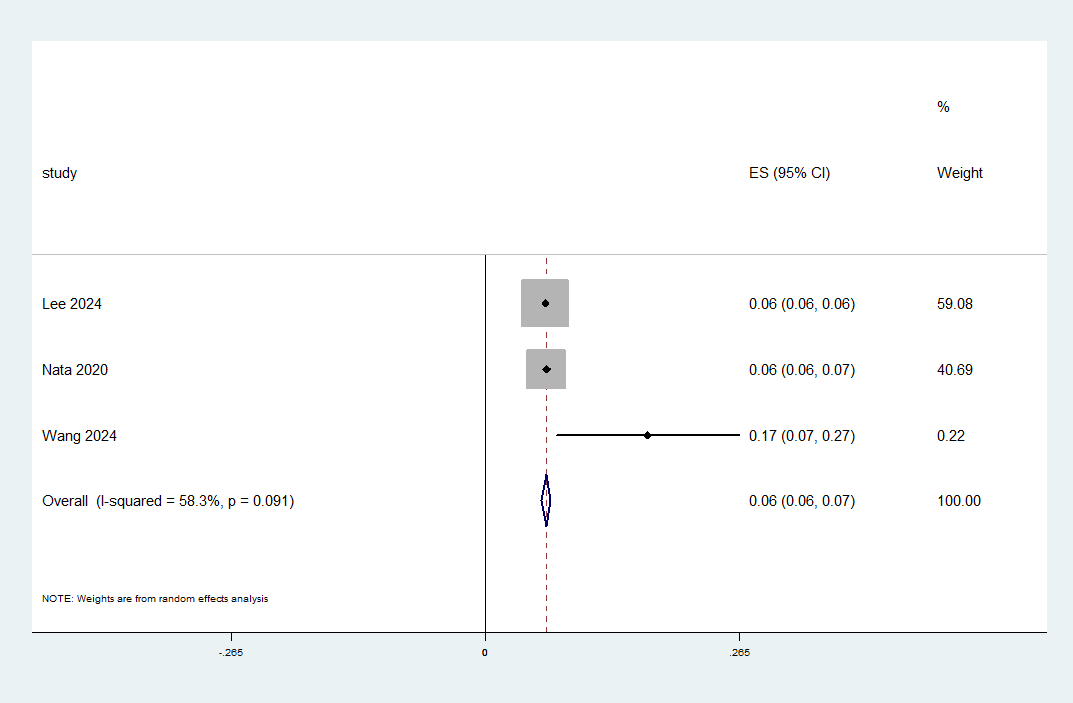


**Figure S33-1.** Forest plot of Sex(Female) as a risk factor for HUA

**
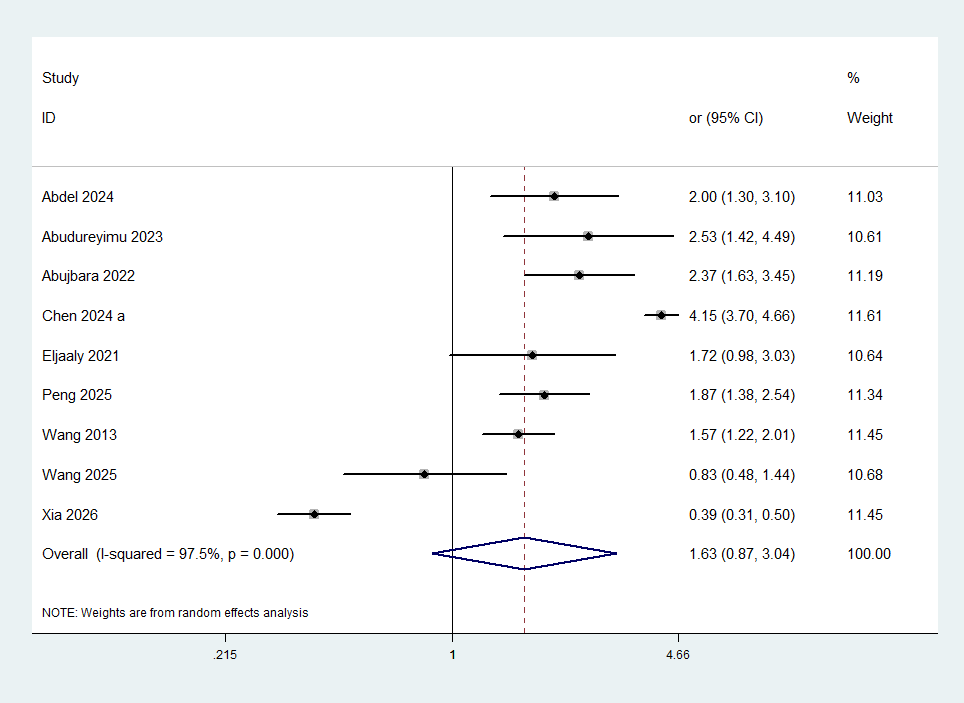
**

**Figure S33-2.** Forest plot of Sex(Female) as a risk factor for HUA stratified by diagnostic criteria

**
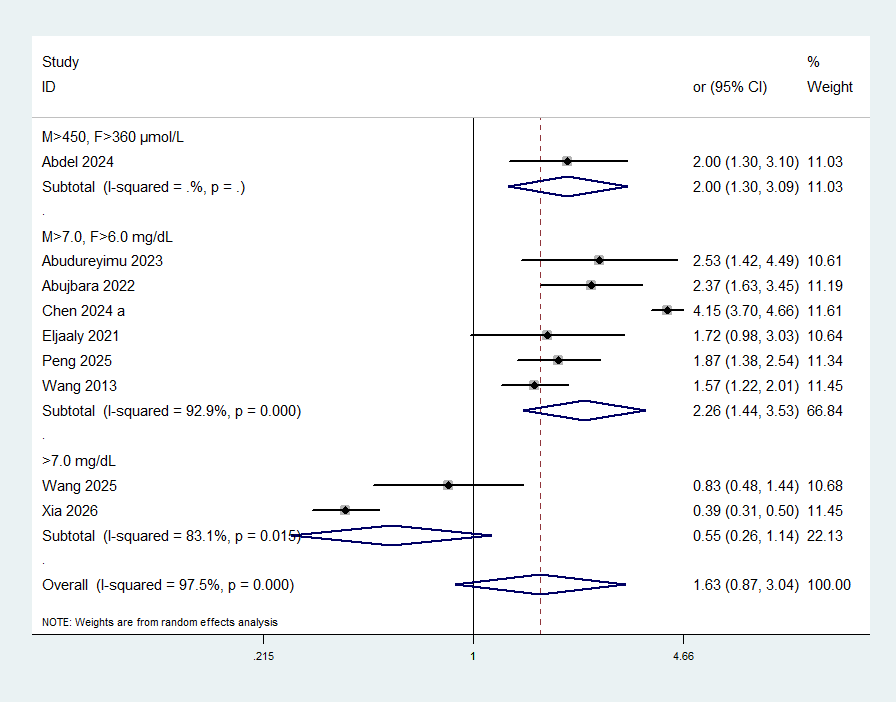
**

**Figure S34-1.** Forest plot of Age as a risk factor for HUA

**
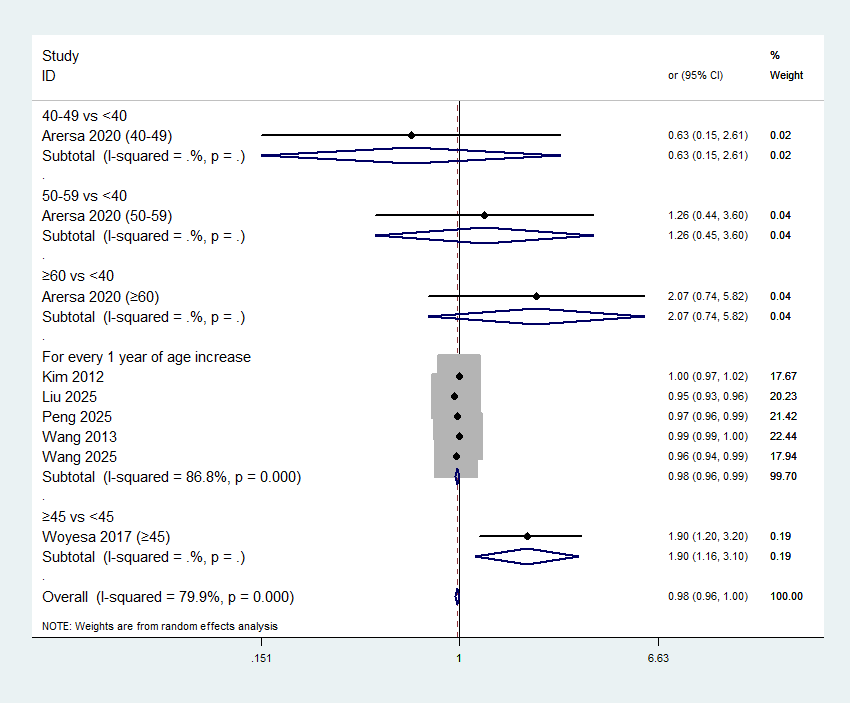
**

**Figure S35-1.** Forest plot of BMI as a risk factor for HUA


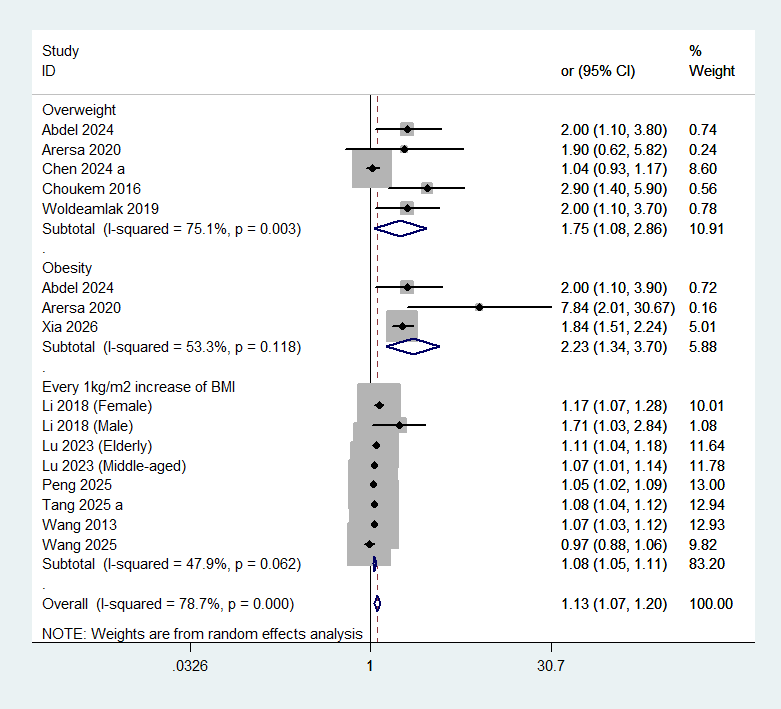


**Figure S35-2.** Contour-enhanced Funnel plot of BMI as a risk factor for HUA


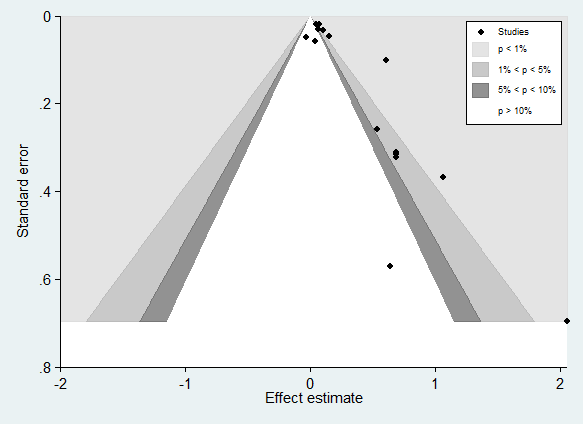


**Figure S35-3.** Sensitivity analysis of BMI as a risk factor for HUA


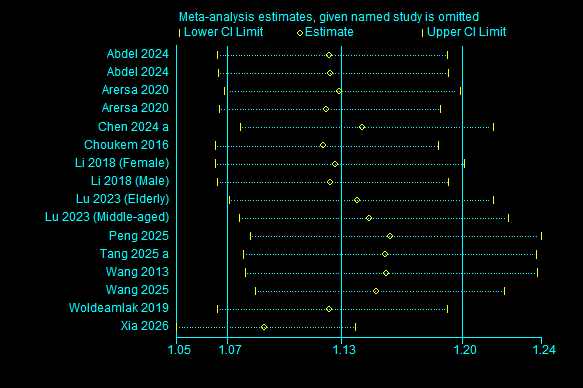


**Figure S36-1.** Forest plot of TG as a risk factor for HUA


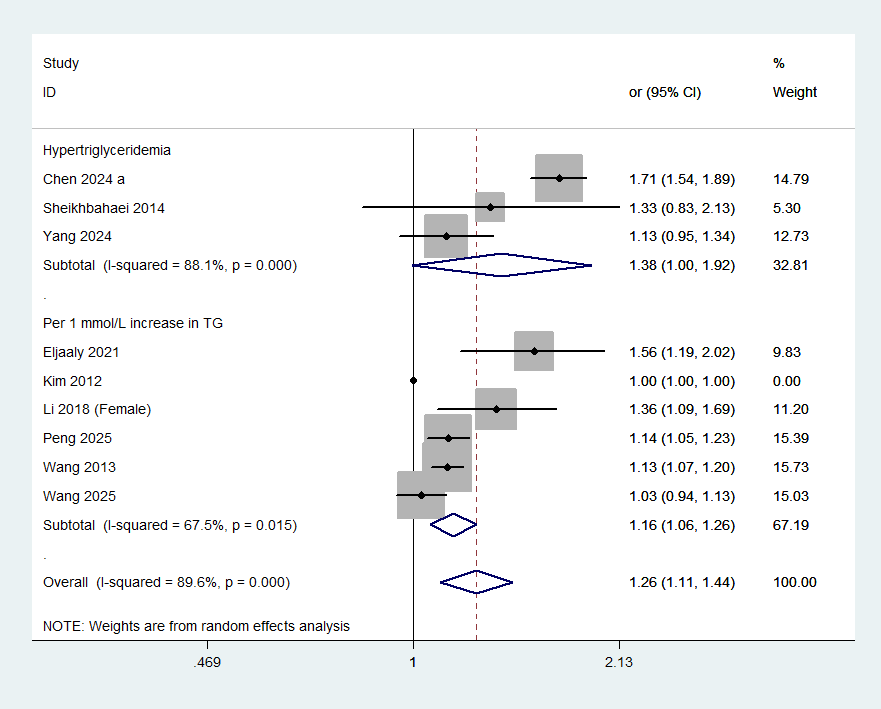


**Figure S36-2.** Sensitivity analysis of TG as a risk factor for HUA

**
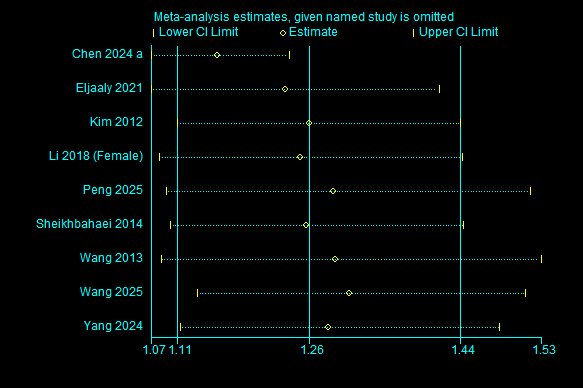
**

**Figure S37-1.** Forest plot of HDL as a risk factor for HUA

**
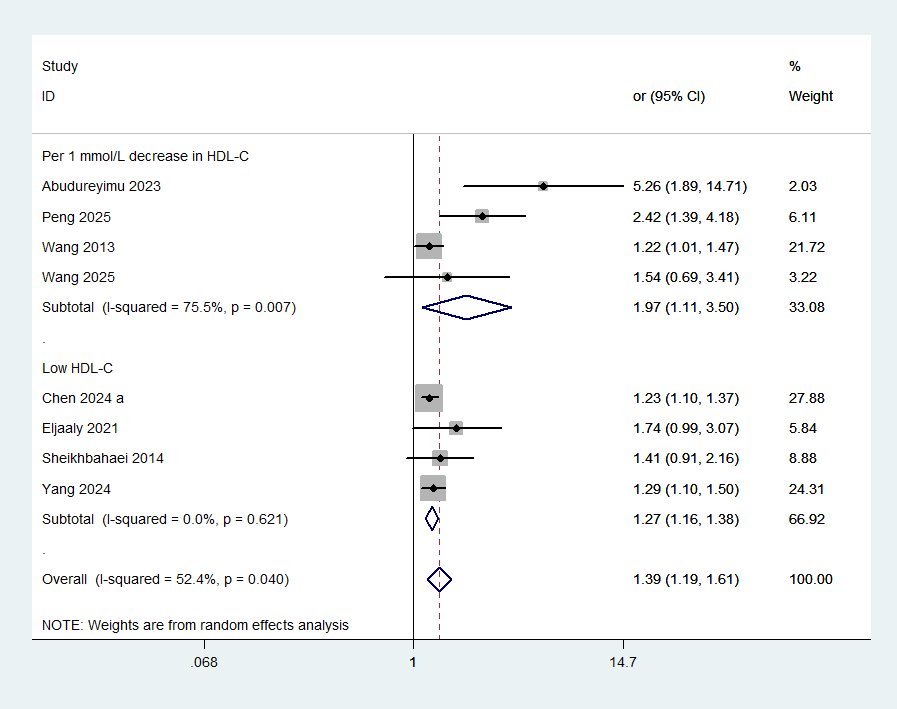
**

**Figure S37-2.** Sensitivity analysis of HDL as a risk factor for HUA

**
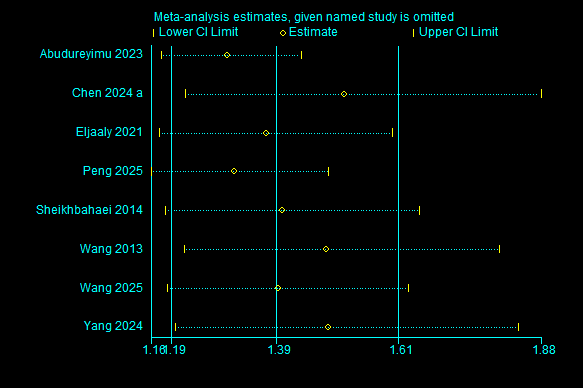
**

**Figure S38.** Forest plot of TC as a risk factor for HUA

**
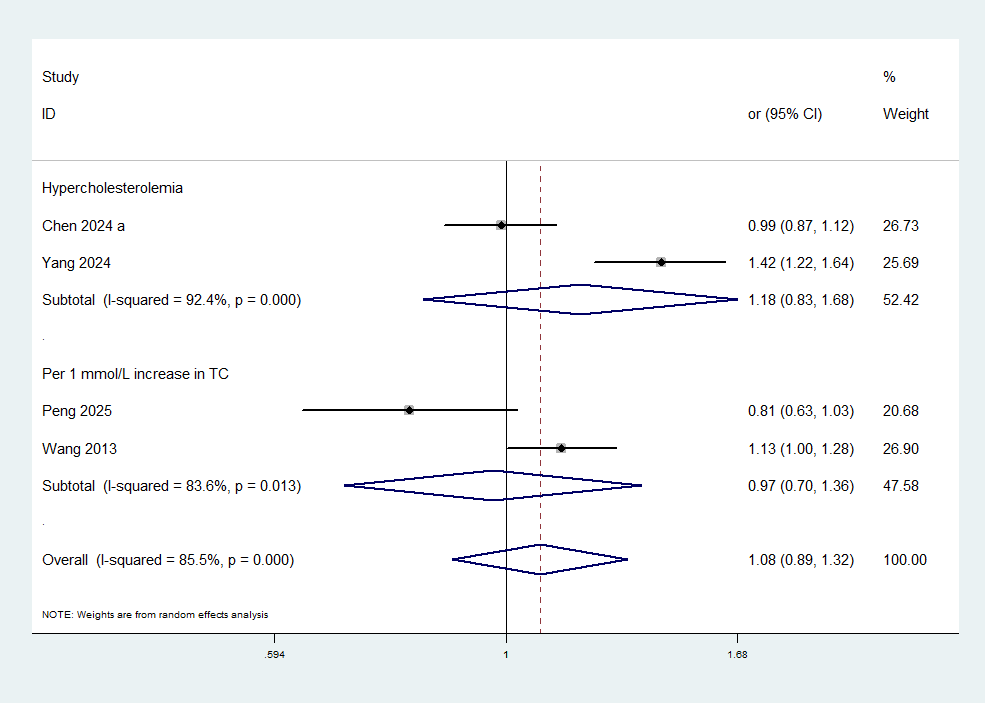
**

**Figure S39.** Forest plot of LDL-C as a risk factor for HUA

**
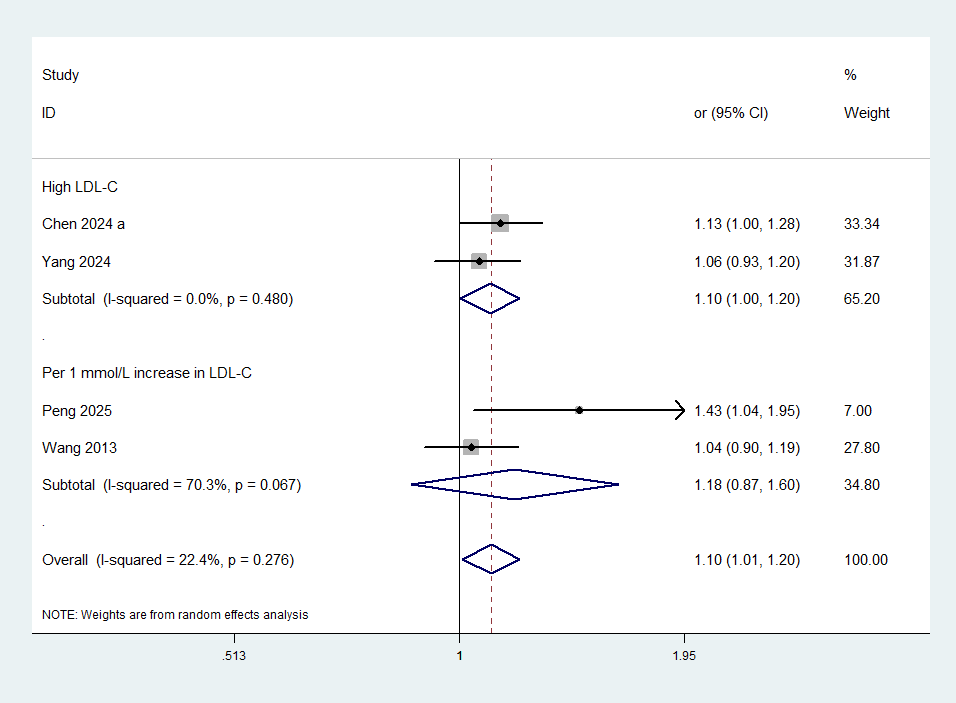
**

**Figure S40-1.** Forest plot of Waist Circumference as a risk factor for HUA

**
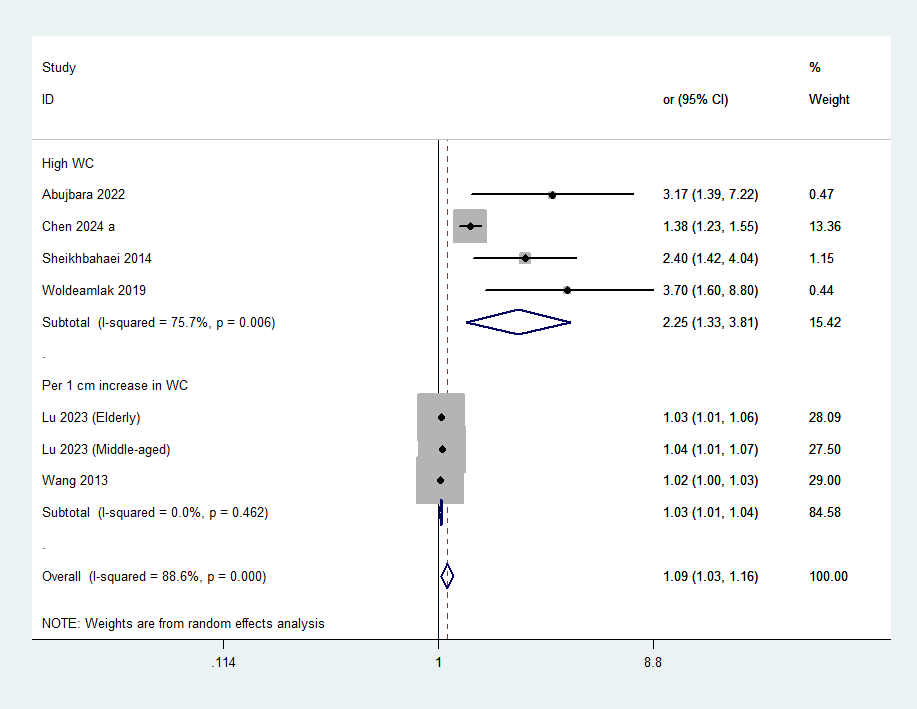
**

**Figure S40-2.** Sensitivity analysis of Waist Circumference as a risk factor for HUA

**
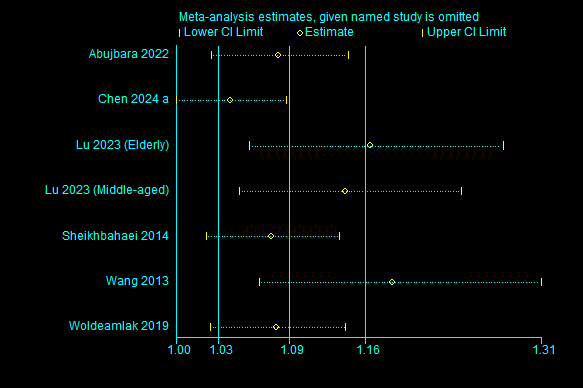
**

**Figure S41.** Forest plot of FPG as a risk factor for HUA

**
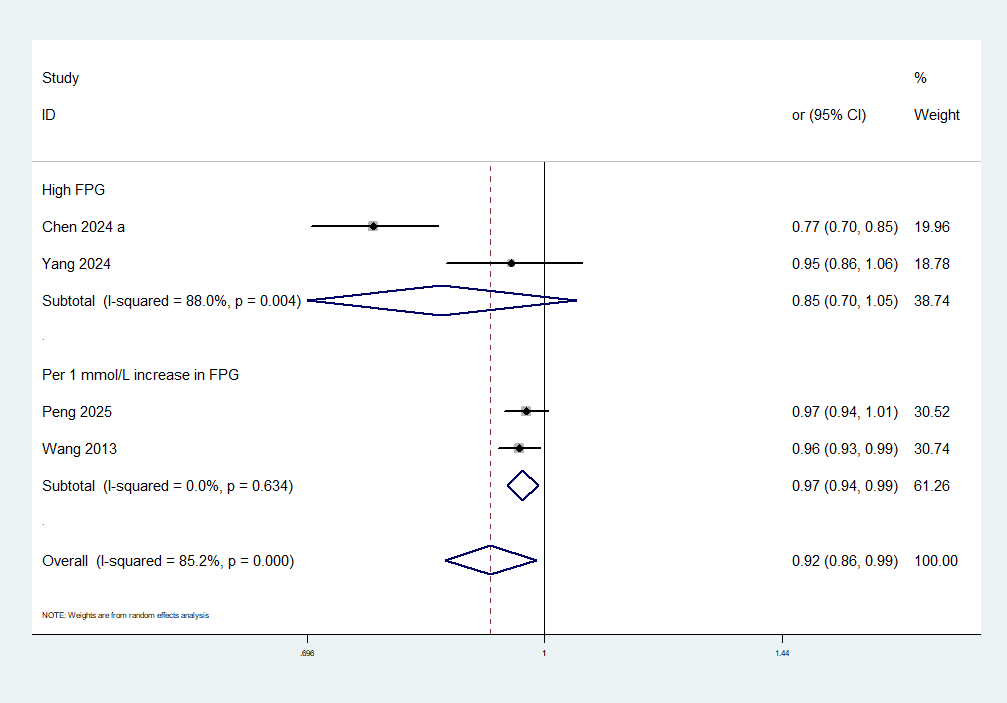
**

**Figure S42.** Forest plot of Hip Circumference as a risk factor for HUA

**
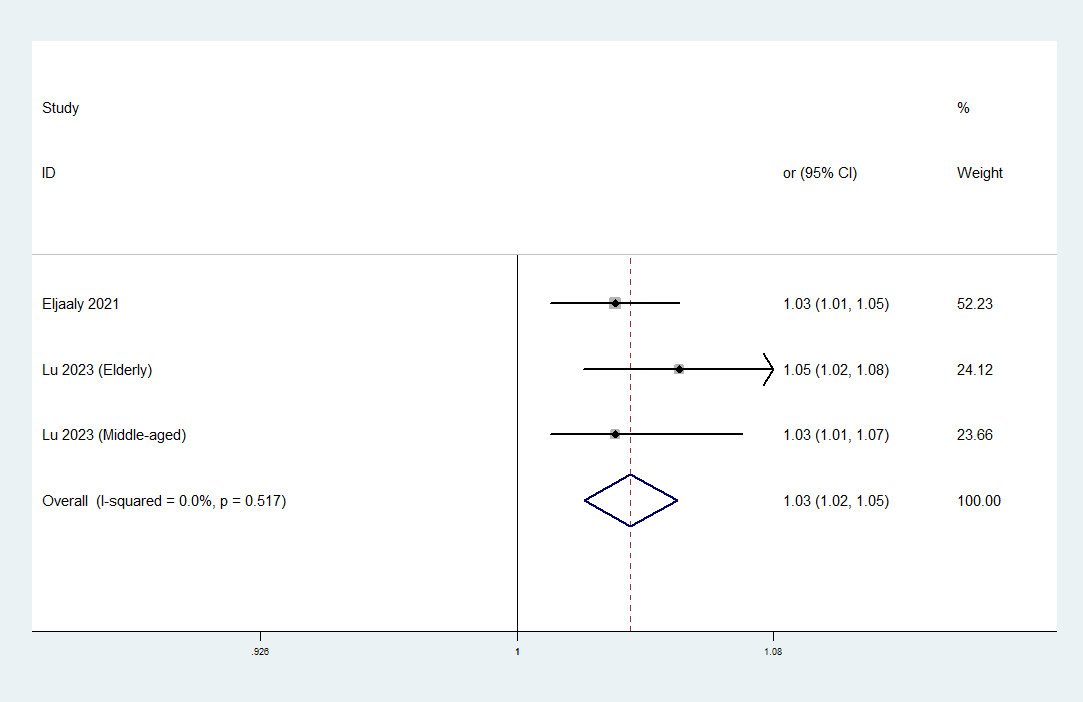
**

**Figure S43-1.** Forest plot of Hypertension as a risk factor for HUA

**
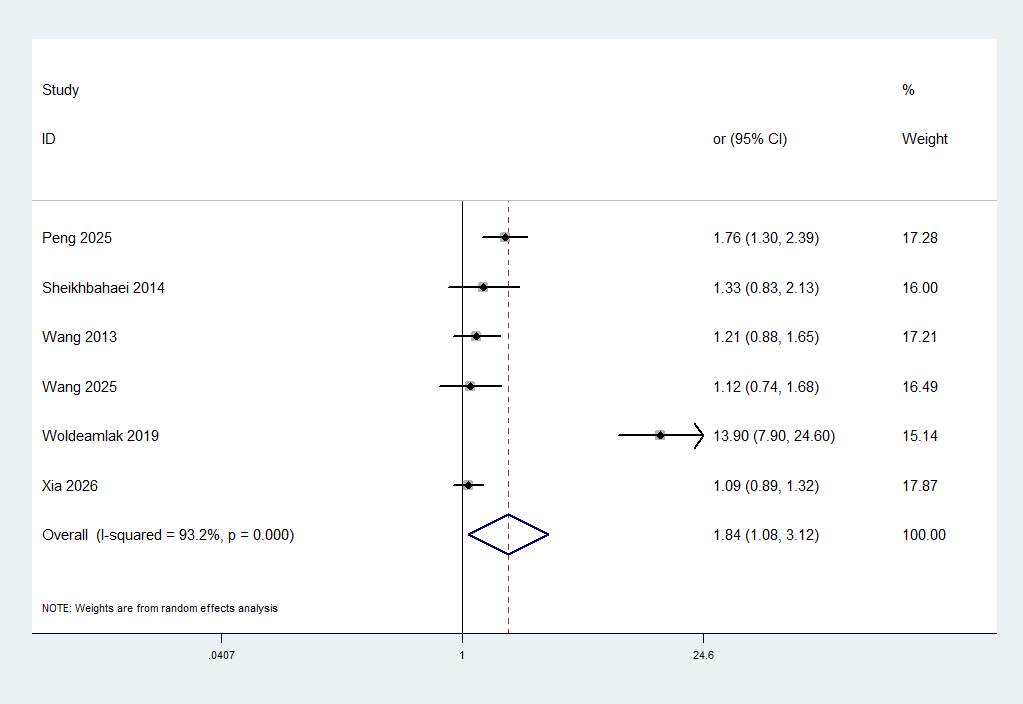
**

**Figure S43-2.** Sensitivity analysis of Hypertension as a risk factor for HUA

**
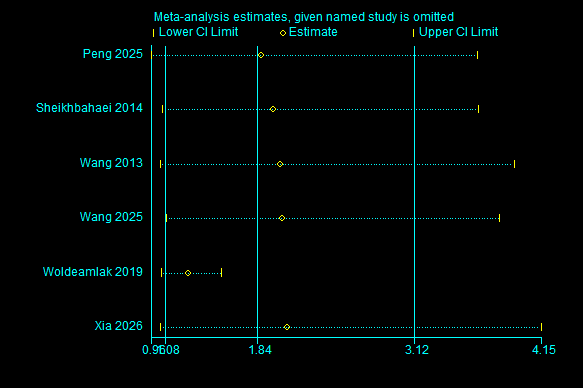
**

**Figure S44.** Forest plot of SBP as a risk factor for HUA

**
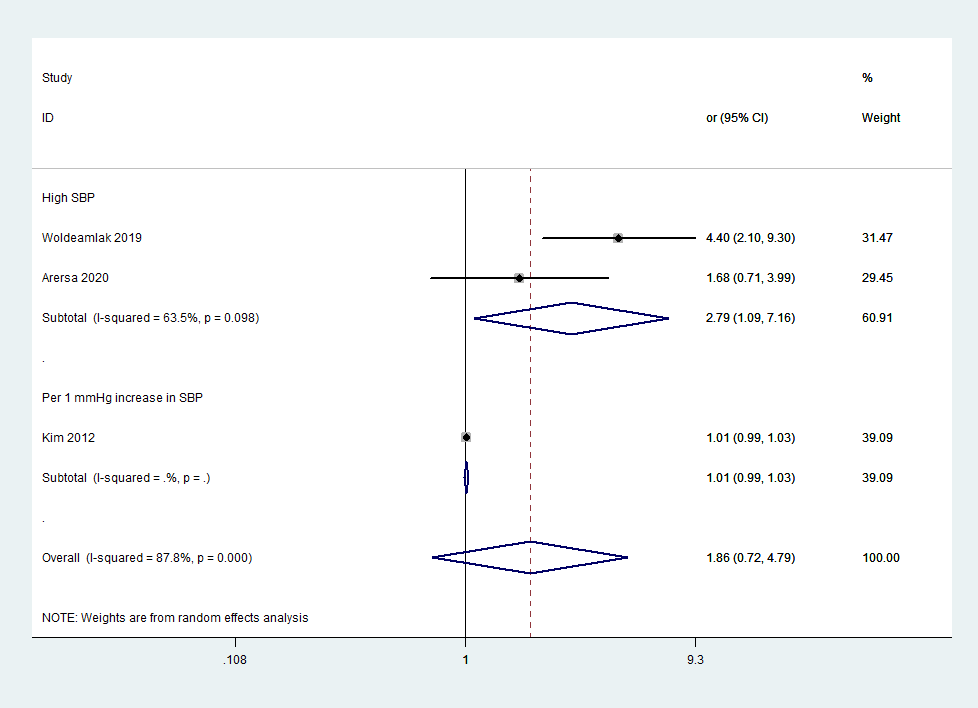
**

**Figure S45.** Forest plot of DBP as a risk factor for HUA

**
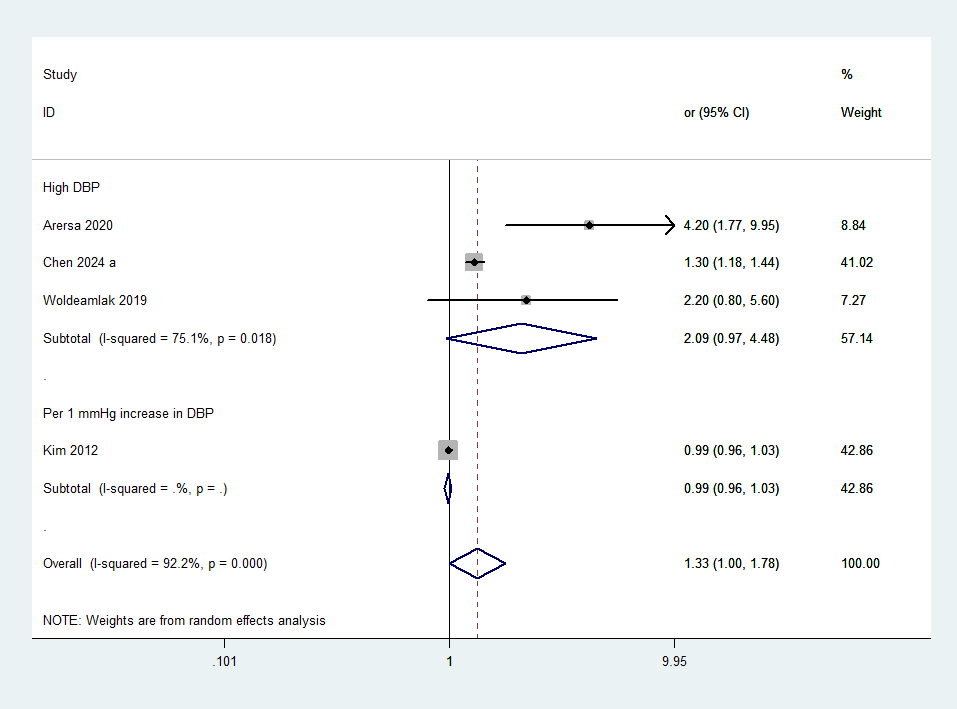
**

**Figure S46.** Forest plot of Duration of T2DM as a risk factor for HUA

**
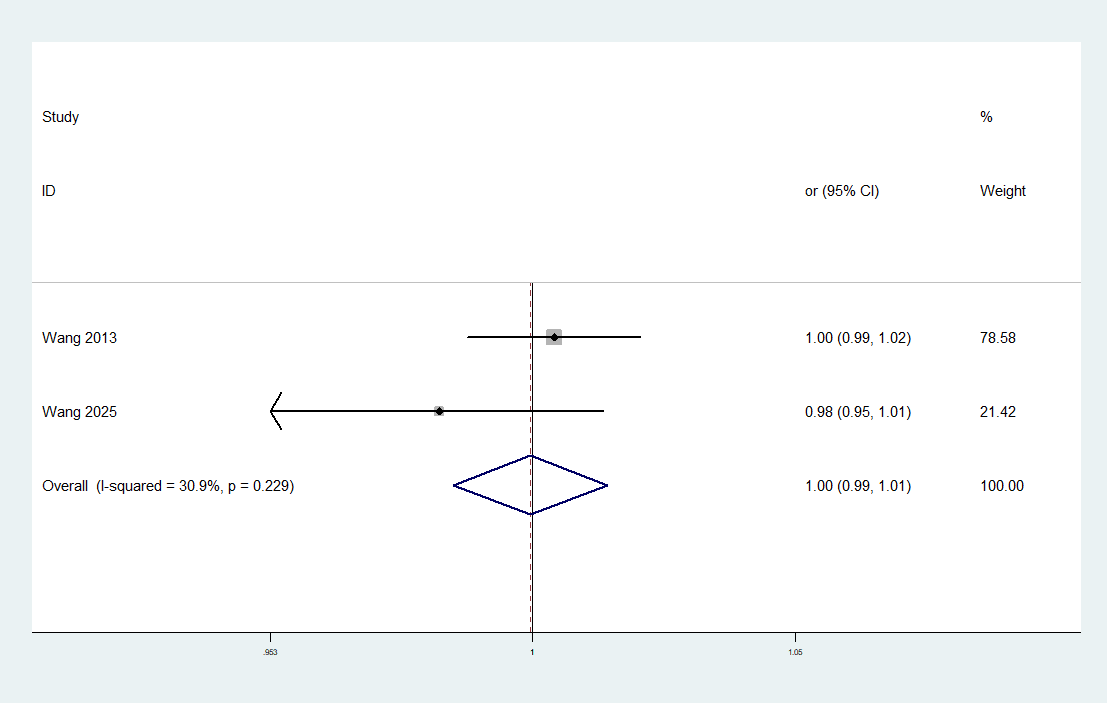
**

**Figure S47.** Forest plot of Mets as a risk factor for HUA

**
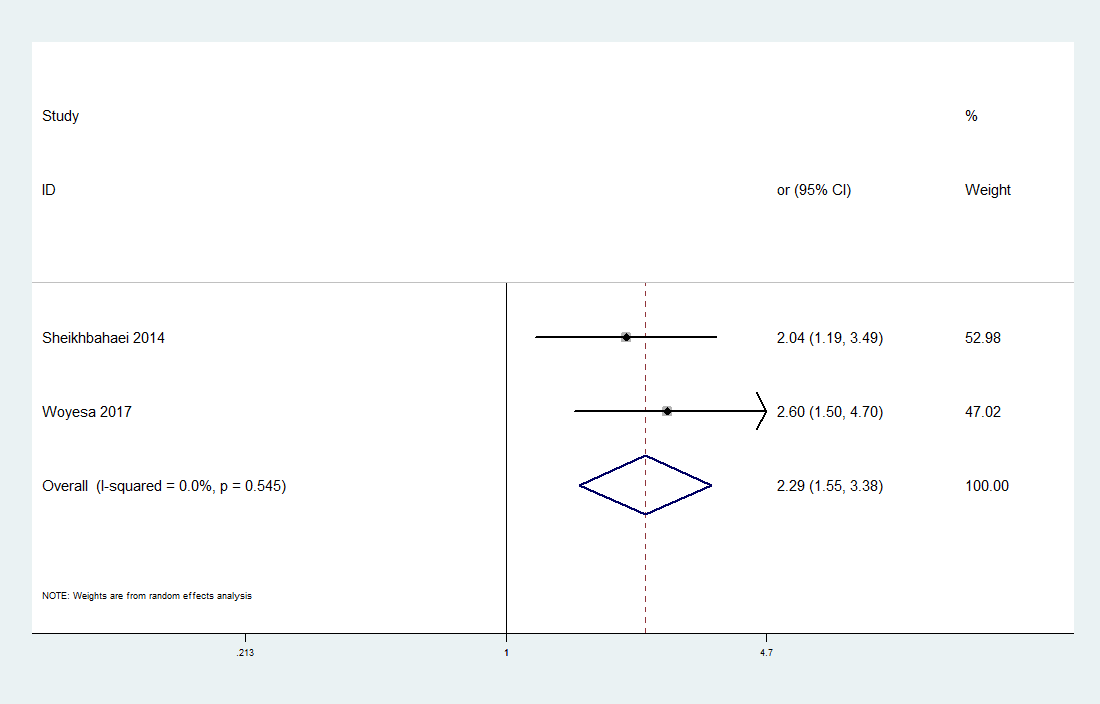
**

**Figure S48-1.** Forest plot of TyG as a risk factor for HUA

**
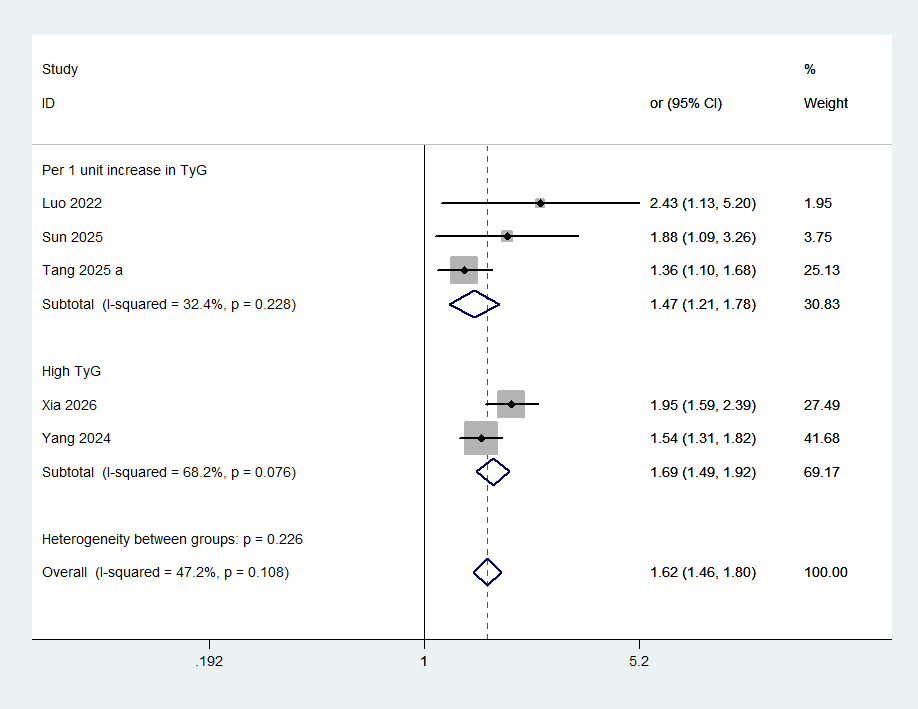
**

**Figure S48-2.** Sensitivity analysis of TyG as a risk factor for HUA

**
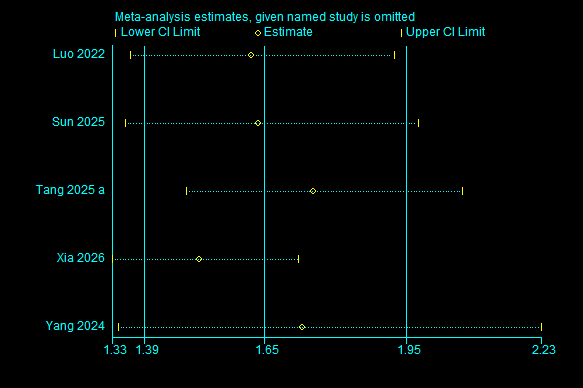
**

**Figure S49.** Forest plot of BUN as a risk factor for HUA

**
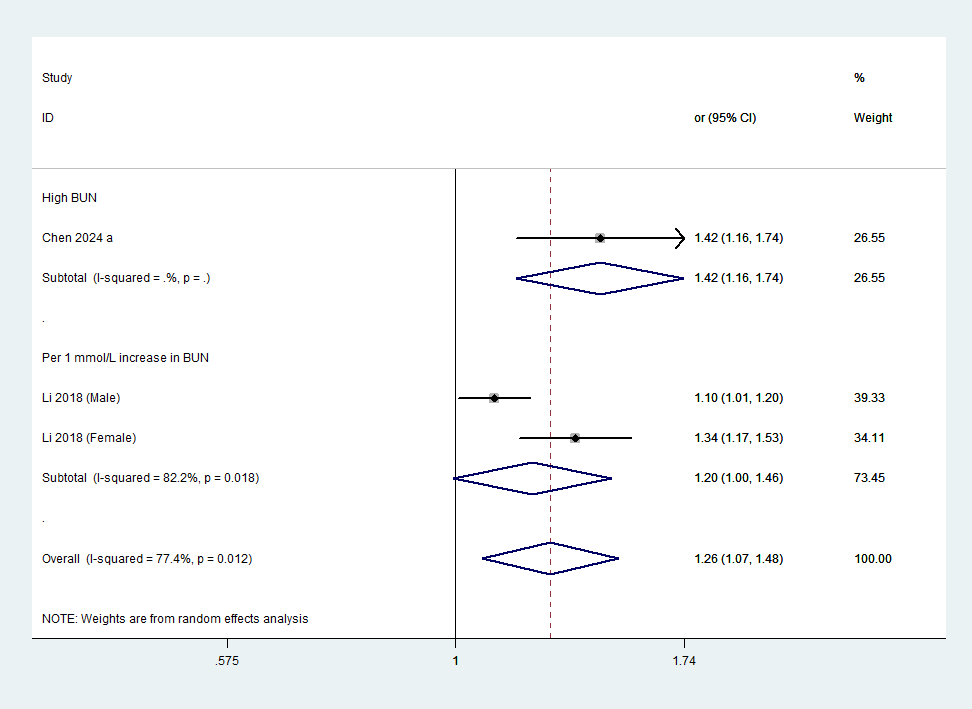
**

**Figure S50.** Forest plot of Smoke as a risk factor for HUA

**
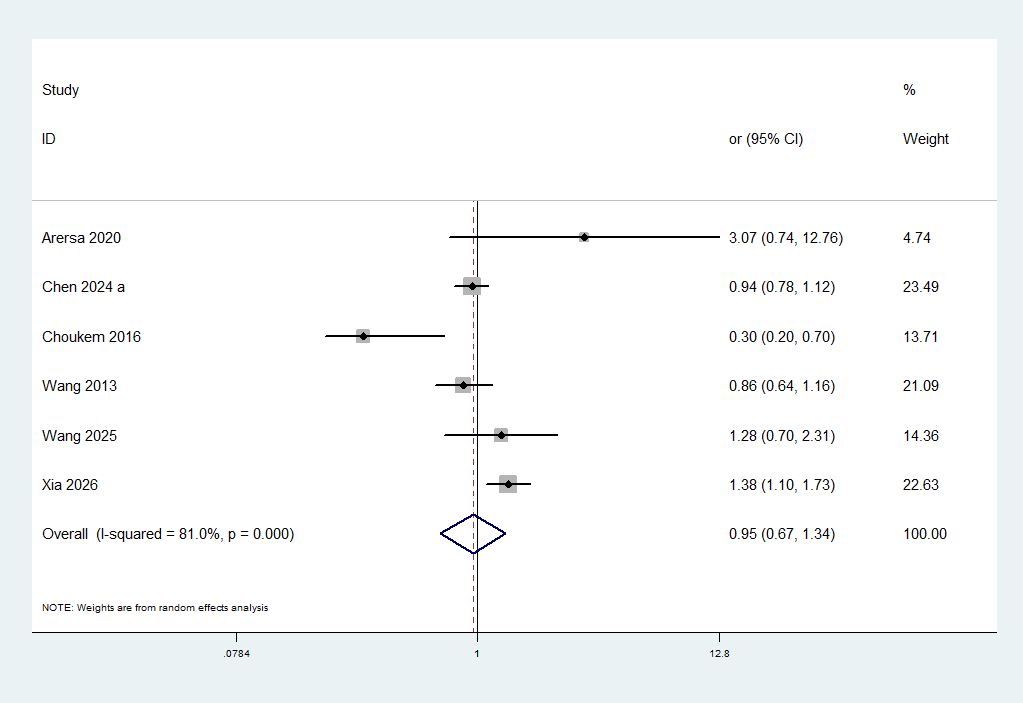
**

**Figure S51-1.** Forest plot of Alcohol Consumption as a risk factor for HUA

**
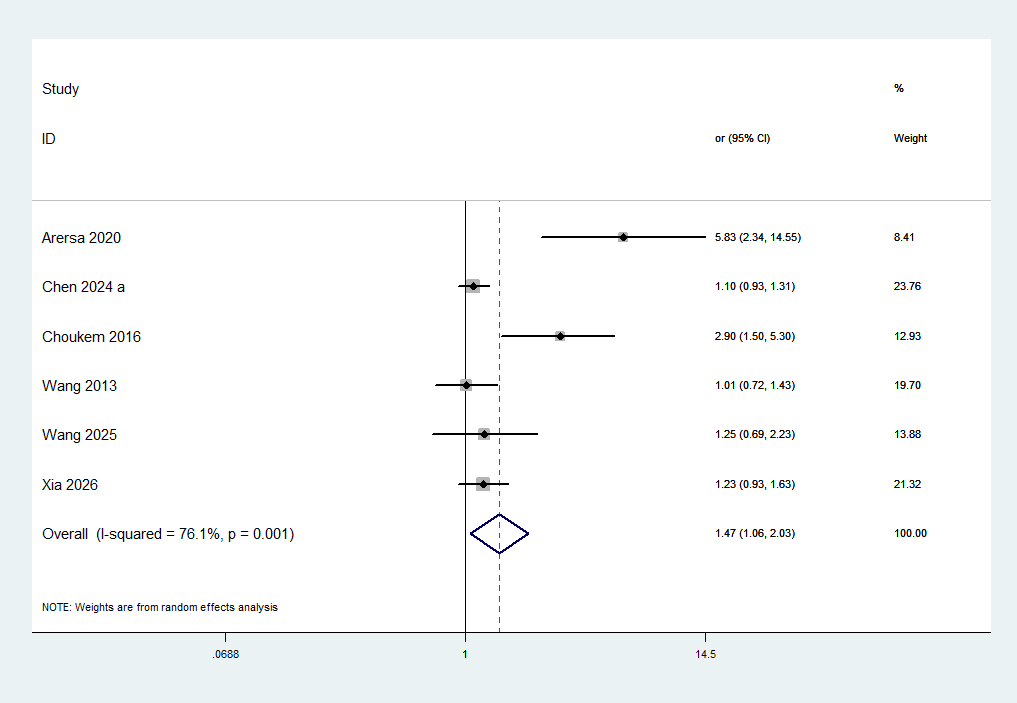
**

**Figure S51-2.** Sensitivity analysis of Alcohol Consumption as a risk factor for HUA

**
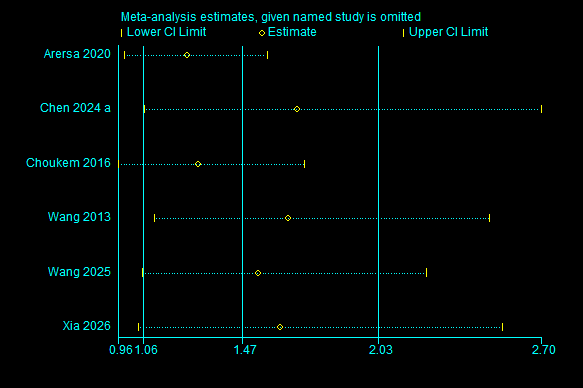
**

**Figure S52-1.** Forest plot of HbA1c as a risk factor for HUA

**
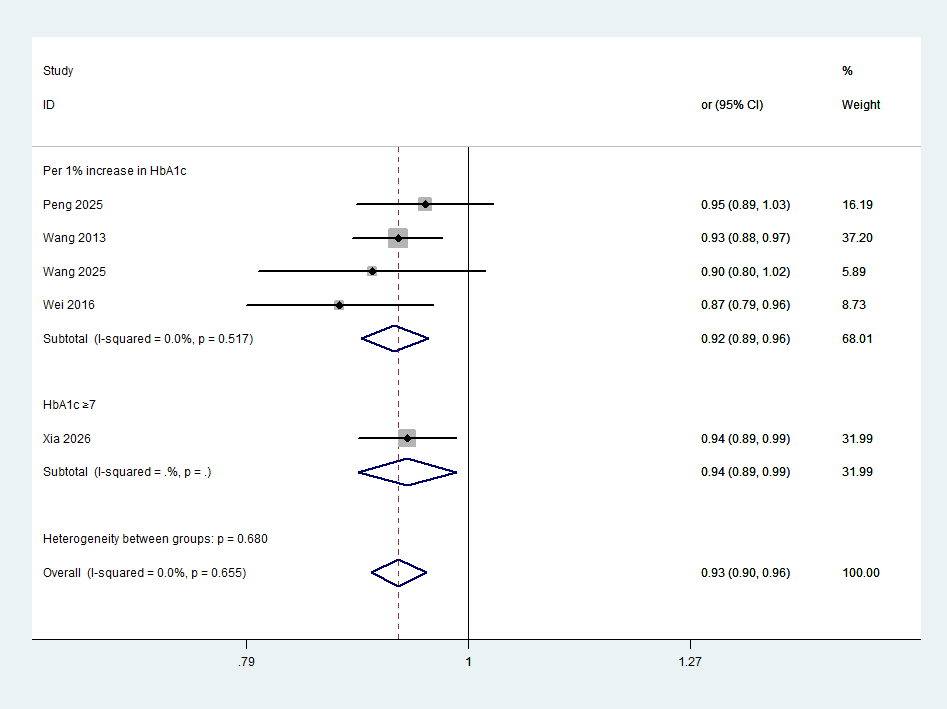
**

**Figure S52-2.** Sensitivity analysis of HbA1c as a risk factor for HUA

**
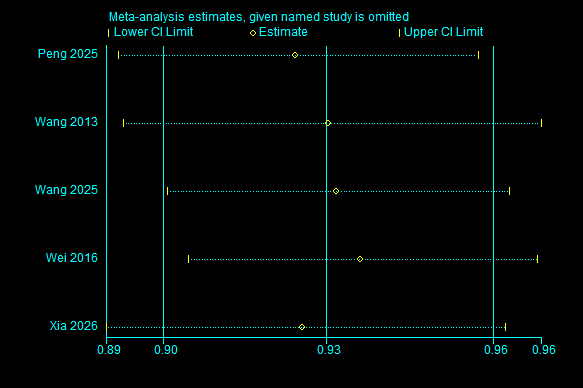
**

**Figure S53.** Forest plot of eGFR as a risk factor for HUA


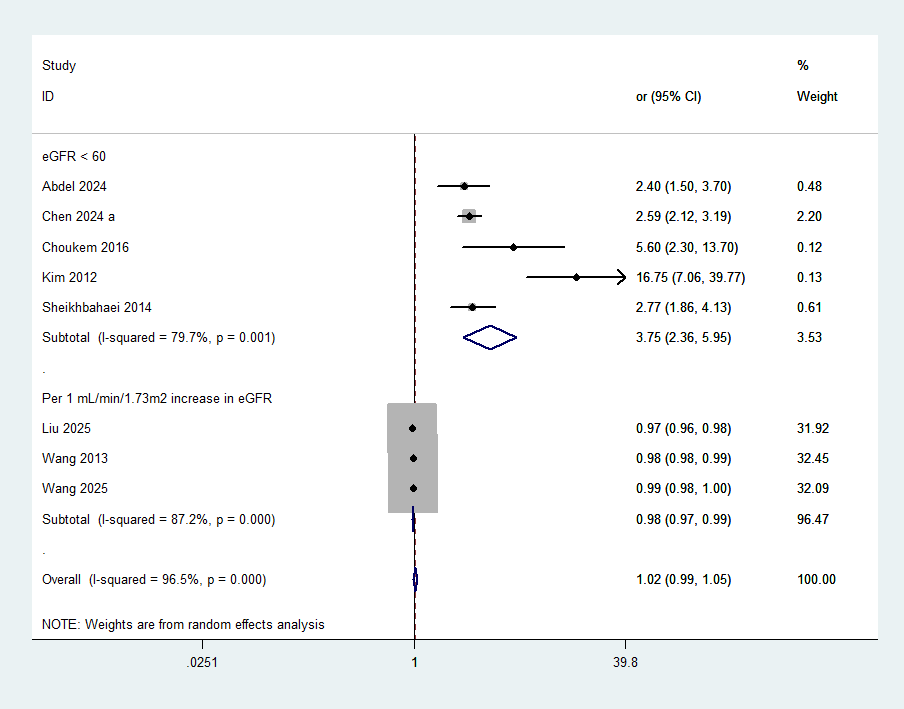


**Figure S54.** Forest plot of Crea as a risk factor for HUA

**
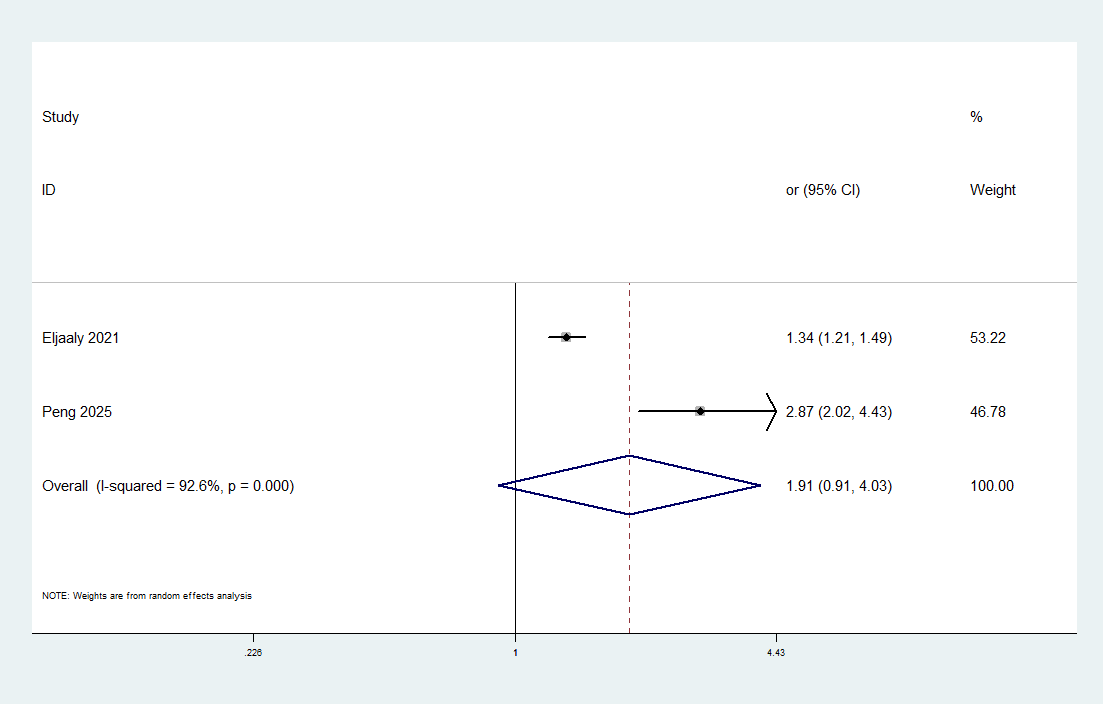
Figure S55.** Forest plot of Sex(Male) as a risk factor for Gout

**
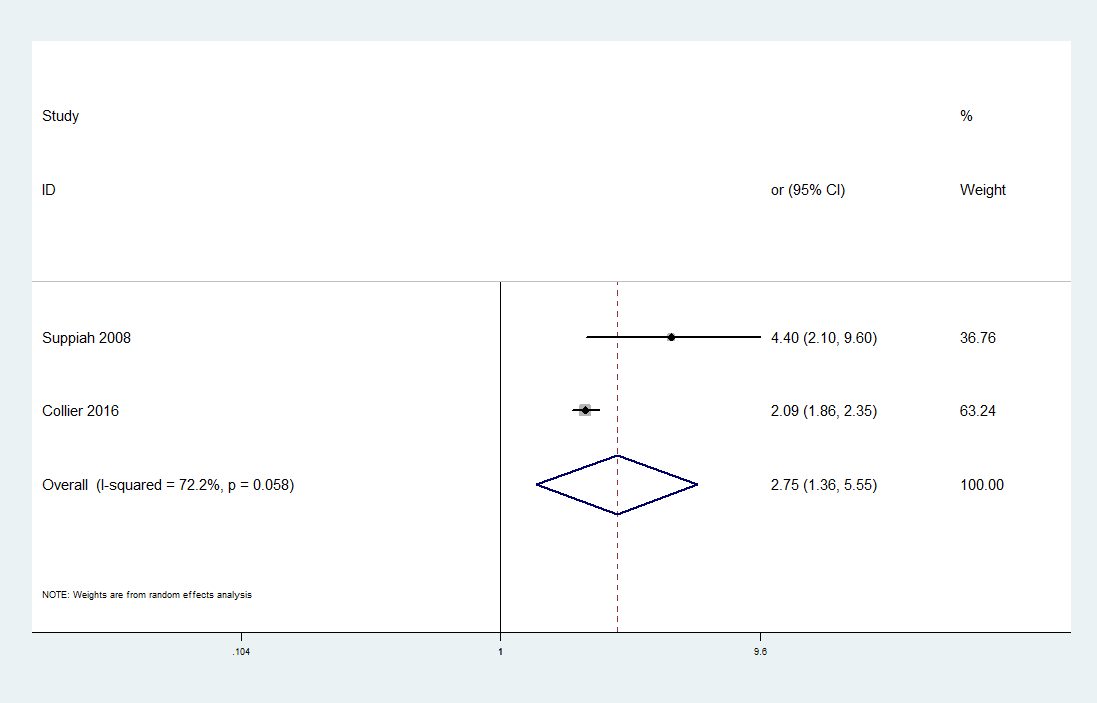
**

**Figure S56.** Forest plot of Hypertriglyceridemia as a risk factor for Gout

**
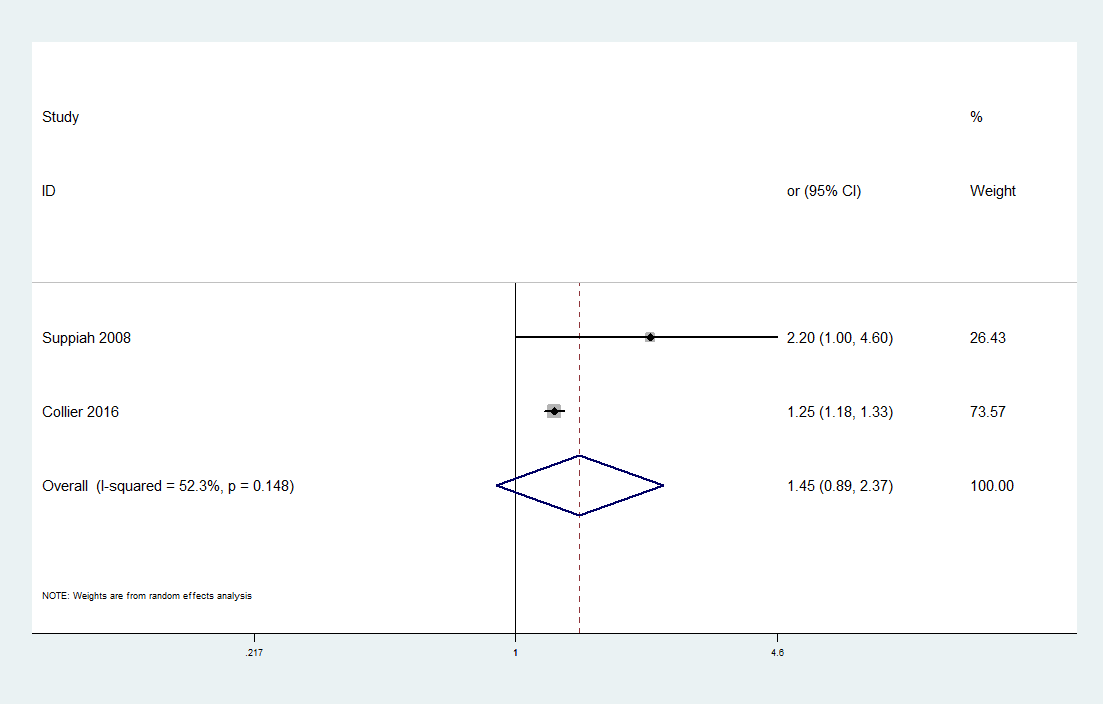
**
